# Supplementary material for: Mapping the Global Cancer Research Funding Landscape
Source: JNCI Cancer Spectr. 2019 Oct 7;3(4):pkz069. doi: 10.1093/jncics/pkz069 (PMC7049992; doi:10.1093/jncics/pkz069)
Supplement: pkz069_Supplementary_Data [file pkz069_supplementary_data.zip › SCHMUTZ_Cancer_Research_Funding_Mapping_Appendix_Revised.docx]

**Appendix A.**

**List of the cancer research funding sources identified in the study.**

*The list is also available from* [*https://www.iarc.fr/list-of-institutions-funding-cancer-research/*](https://www.iarc.fr/list-of-institutions-funding-cancer-research/)*. Institutions can request modifications in the list, following the instructions provided.*

*Note that organizations that have ceased to exist or merged are still listed as separate entities if they have been active during the 2008-2018 period (approx. 50 organizations).*

**Governmental organizations**

| National Agency for Scientific and Technological Promotion - ANPCyT, Argentina |
| --- |
| National Cancer Institute of Argentina, Argentina |
| National Scientific and Technical Research Council - CONICET, Argentina |
| ACT Health Research Office, Australia |
| Australian Agency For International Development, Australia |
| Australian Nuclear Science and Technology Organisation - ANSTO, Australia |
| Australian Research Council, Australia |
| Cancer Australia, Australia |
| Cancer Institute New South Wales, Australia |
| Commonwealth Scientific And Industrial Research Organisation - CSIRO, Australia |
| Department of Health of Australia, Australia |
| Department of Industry, Innovation and Science of Australia, Australia |
| Department of Veterans' Affairs of Australia, Australia |
| Foreign Investment Review Board, Australia |
| Government of Australia, Australia |
| Government of Queensland, Australia |
| Insurance Commission of Western Australia, Australia |
| National Health and Medical Research Council - NHMRC, Australia |
| National Library of Australia, Australia |
| Regional Government of South Australia, Australia |
| State of Victoria Government, Australia |
| Therapeutic Innovation Australia, Ltd., Australia |
| Victorian Cancer Agency, Australia |
| Austrian Academy of Sciences, Austria |
| Austrian National Bank, Austria |
| Austrian Research Promotion Agency - FFG, Austria |
| Austrian Science Fund, Austria |
| Federal Ministry for Transport, Innovation and Technology of Austria, Austria |
| Federal Ministry of Economy Family And Youth of Austria, Austria |
| Federal Ministry of Education of Austria, Austria |
| Federal Ministry of Health of Austria, Austria |
| Federal Ministry of Science, Research and Economy of Austria, Austria |
| Salzburg Provincial Government, Austria |
| Vienna Municipal Government, Austria |
| National Oncology Centre of Azerbaijan Republic, Azerbaijan |
| Belgian Federal Public Service Health, Food Chain Safety and Environment, Belgium |
| Belgian Federal Science Policy Office - BELSPO, Belgium |
| Flanders Innovation and Entrepreneurship, Belgium |
| Flemish Government, Belgium |
| Flemish Interuniversity Council - University Cooperation for Development / VLIR - UOS, Belgium |
| Government of Belgium, Belgium |
| Walloon Government, Belgium |
| Coordination For The Improvement of Higher Education Personnel - CAPES, Brazil |
| Funding Authority for Studies and Projects - FINEP, Brazil |
| Ministry of Health of Brazil, Brazil |
| National Council for Scientific and Technological Development - CNPq, Brazil |
| National Institute for Science and Technology on Cancer - INCT, Brazil |
| Ministry of Health of Bulgaria, Bulgaria |
| Alberta Innovates Health Solutions, Canada |
| BC Cancer Agency, Canada |
| British Columbia Cancer Agency, Canada |
| Canada Research Chairs Program, Canada |
| Canadian Institutes of Health Research - CIHR, Canada |
| Canadian Partnership Against Cancer, Canada |
| Canadian Stem Cell Network, Canada |
| Cancer Care Ontario, Canada |
| Cancercare Manitoba, Canada |
| Council of Ministers of Education of Canada, Canada |
| Fonds de recherche du Québec, Canada |
| Genome British Columbia, Canada |
| Genome Canada, Canada |
| Genome Quebec, Canada |
| Government of Canada, Canada |
| Government of Quebec, Canada |
| Health Canada, Canada |
| Manitoba Health Research Council, Canada |
| Manitoba Medical Service Foundation, Canada |
| Michael Smith Foundation for Health Research, Canada |
| National Research Council of Canada - CNRC-NRC, Canada |
| Natural Sciences and Engineering Research Council of Canada - NSERC, Canada |
| Nova Scotia Provincial Government - Nova Scotia Health Authority, Canada |
| Ontario Genomics, Canada |
| Ontario Provincial Government, Canada |
| Public Health Agency of Canada, Canada |
| Research Manitoba, Canada |
| Social Sciences and Humanities Research Council, Canada |
| Statistics Canada, Canada |
| The Alberta Cancer Foundation, Canada |
| WorkSafeBC, Canada |
| Ministry of Education of Chile, Chile |
| National Commission for Scientific and Technological Research - CONICYT, Chile |
| Anhui Provincial Government, China |
| Beijing Municipal Government, China |
| Changzhou Municipal Government, China |
| Chinese Academy of Agricultural Sciences, China |
| Chinese Academy of Medical Sciences - CAMS, China |
| Chinese Academy of Sciences - CAS, China |
| Chinese Government, China |
| Chongqing Municipal Government, China |
| Dalian Municipal Government, China |
| Dongguan Municipal Government, China |
| Fujian Provincial Government, China |
| Gansu Provincial Government, China |
| Government of Macau, China |
| Guangdong Provincial Government, China |
| Guangxi Provincial Government, China |
| Guangzhou Municipal Government, China |
| Guilin Municipal Government, China |
| Guizhou Provincial Government, China |
| Hainan Provincial Government, China |
| Hangzhou Municipal Government, China |
| Harbin Municipal Government, China |
| Hebei Provincial Government, China |
| Heilongjiang Provincial Government, China |
| Henan Provincial Government, China |
| Hong-Kong Municipal Government, China |
| Hubei Provincial Government, China |
| Hunan Provincial Government, China |
| Huzhou Municipal Government, China |
| Inner Mongolia Provincial Government, China |
| Jiangsu Provincial Government, China |
| Jiangxi Provincial Government, China |
| Jiaxing Municipal Government, China |
| Jilin Provincial Government, China |
| Jinan Municipal Government, China |
| Kunshan Municipal Government, China |
| Lanzhou Municipal Government, China |
| Liaoning Provincial Government, China |
| Liuzhou Municipal Government, China |
| Ministry of Agriculture of the People's Republic of China, China |
| Ministry of Education of the People's Republic of China, China |
| Ministry of Finance of the People's Republic of China, China |
| Ministry of Health of the People's Republic of China, China |
| Ministry of Human Resources and Social Security of the People's Republic of China, China |
| Ministry of Science and Technology of People's Republic of China, China |
| Ministry of Science and Technology of the People's Republic of China, China |
| Nanjing Municipal Government, China |
| Nantong Government, China |
| Nantong Municipal Government, China |
| National Natural Science Foundation of China, China |
| Ningbo Municipal Government, China |
| Pudong Municipal Government, China |
| Qingdao Municipal Government, China |
| Shaanxi Provincial Government, China |
| Shandong Provincial Government, China |
| Shanghai Municipal Government, China |
| Shenyang Municipal Government, China |
| Shenzhen Municipal Government, China |
| Sichuan Provincial Government, China |
| South China Sea Institute of Oceanology, China |
| Suzhou Municipal Government, China |
| Taizhou Municipal Government, China |
| Tianjin Municipal Government, China |
| Tibet Provincial Government, China |
| Wenzhou Municipal Government, China |
| Wuhan Municipal Government, China |
| Wuxi Municipal Government, China |
| Xiamen Municipal Government, China |
| Xinjiang Provincial Government, China |
| Xuzhou Municipal Government, China |
| Yangzhou Municipal Government, China |
| Yantai Municipal Government, China |
| Yunnan Provincial Government, China |
| Zhanjiang Municipal Government, China |
| Zhejiang Provincial Government, China |
| Zhenjiang Municipal Government, China |
| Administrative Department of Science, Technology And Innovation of Colombia, Colombia |
| National Cancer Institute of Colombia, Colombia |
| Ministry of Science, Education and Sports of the Republic of Croatia, Croatia |
| Ministry of Public Health of Cuba, Cuba |
| Cyprus Research Promotion Foundation, Cyprus |
| Academy of Sciences of the Czech Republic, Czech Republic |
| Czech Health Research Council, Czech Republic |
| Government of the Czech Republic, Czech Republic |
| Ministry of Education, Youth and Sport of the Czech Republic, Czech Republic |
| Ministry of Health of the Czech Republic, Czech Republic |
| Ministry of Industry and Trade of the Czech Republic, Czech Republic |
| Slovak Research and Development Agency - APVV, Czech Republic |
| Slovenian Research Agency - ARRS, Czech Republic |
| Capital Region of Denmark Government, Denmark |
| Central Denmark Regional Government, Denmark |
| Danish Agency For Science Technology And Innovation, Denmark |
| Danish Council For Independent Research, Denmark |
| Danish Council For Strategic Research, Denmark |
| Danish National Advanced Technology Foundation, Denmark |
| Ministry of Health of Denmark, Denmark |
| Regional Government of Southern Denmark, Denmark |
| Government of Egypt, Egypt |
| Ministry of Higher Education of Egypt, Egypt |
| National Cancer Institute of Cairo, Egypt |
| Enterprise Estonia, Estonia |
| Estonian Research Council, Estonia |
| Government of Estonia, Estonia |
| Ministry of Education and Research of Estonia, Estonia |
| Academy of Finland, Finland |
| Business Finland, Finland |
| Government of Finland, Finland |
| Ministry of Education and Culture of Finland, Finland |
| Ministry of Social Affairs and Health of Finland, Finland |
| Northern Savonia Regional Council, Finland |
| Agence De La Biomédecine - Biomedicine Agency, France |
| Agence Nationale De La Recherche - ANR, France |
| Agence Nationale De Recherches Sur Le Sida Et Les Hépatites Virales, France |
| Agence Nationale De Sécurité Sanitaire De L’Alimentation, De L’Environnement Et Du Travail - ANSES, France |
| Aquitaine Regional Council, France |
| Association Nationale de la Recherche et de la Technologie - ANRT, France |
| Bourgogne-France-Comté Regional Council, France |
| Cancéropôle Grand Est, France |
| Cancéropôle Grand Sud-Ouest, France |
| Cancéropôle Ile-De-France, France |
| Cancéropôle Lyon Auvergne Rhône-Alpes, France |
| Cancéropôle Nord-Ouest, France |
| Cancéropôle Paca, France |
| Centre National de la Recherche Scientifique - CNRS, France |
| Commissariat À L'Energie Atomique - CEA, France |
| Conseils Départementaux de France, France |
| Direction Générale De La Santé, France |
| Government of France, France |
| Government of France - Programme Investissements d'Avenir, France |
| Hauts-de-France Regional Council, France |
| Ile-de-France Regional Council, France |
| Indo French Centre for Promotion of Advanced Research, France |
| Institut de Recherche en Santé Publique - GIS-IReSP, France |
| Institut de recherche pour le Développement - IRD, France |
| Institut de veille sanitaire - InVS, France |
| Institut National de la Recherche Agronomique - INRA, France |
| Institut National de la Santé et de la Recherche Médicale - INSERM, France |
| Institut National du Cancer - INCa, France |
| Institut Universitaire de France, France |
| Limousin Regional Council, France |
| Métropole de Lyon, France |
| Ministry of Foreign Affairs and International Development of France, France |
| Ministry of Higher Education, Research and Innovation of France, France |
| Ministry of Social Affairs and Employment of France, France |
| Ministry of Social Affairs and Health of France, France |
| Nantes Municipal Government, France |
| Regional Council of Auvergne Rhône-Alpes, France |
| Regional council of Bretagne, France |
| Regional council of Centre, France |
| Regional Council of Hauts-de-France, France |
| Regional Council of Ile-de-France, France |
| Regional Council of Normandie, France |
| Regional council of Nouvelle-Aquitaine, France |
| Regional Council of Occitanie, France |
| Regional Council of Pays-de-la-Loire, France |
| Regional Council of Provence-Alpes-Côte-d'Azur, France |
| Santé publique France, France |
| Baden-Württemberg State Government, Germany |
| Federal Ministry for Family Affairs, Senior Citizens, Women and Youth of Germany, Germany |
| Federal Ministry for Health of Germany, Germany |
| Federal Ministry of Education and Research of Germany - BMBF, Germany |
| Federal Ministry of Labour and Social Affairs of Germany, Germany |
| German Academic Exchange Service - DAAD, Germany |
| German Federal Office for Radiation Protection, Germany |
| German Social Accident Insurance - DGUV, Germany |
| Government of Germany, Germany |
| Hamburg Government, Germany |
| Helmholtz Association of German Research Centers, Germany |
| Hessen State Government, Germany |
| Lower Saxony State Government, Germany |
| North Rhine-Westphalia State Government, Germany |
| Saxony State Government, Germany |
| State of Bavaria Government, Germany |
| General Secretariat for Research and Technology, Greece |
| Greek General Secretariat for Research and Technology, Greece |
| Greek Government, Greece |
| Ministry of Culture, Education and Religious Affairs of Greece, Greece |
| Ministry of Health of Greece, Greece |
| State Scholarships Foundation, Greece |
| Government of Hungary, Hungary |
| Hungarian Academy of Sciences, Hungary |
| Ministry of Human Capacities of Hungary, Hungary |
| Ministry of National Resources of Hungary, Hungary |
| National Research, Development and Innovation Office of Hungary, Hungary |
| National Research, Development and Innovation Office of Hungary - Hungarian Scientific Research Fund - OTKA, Hungary |
| The Icelandic Centre for Research - Rannis, Iceland |
| The University of Iceland Research Fund, Iceland |
| Council For Scientific And Industrial Research - CSIR, India |
| Government of India, India |
| Indian Council of Medical Research, India |
| Indian Department of Atomic Energy, India |
| Ministry of Electronics and Information Technology of India, India |
| Ministry of Health and Family Welfare of India, India |
| Ministry of Science and Technology of India, India |
| Minitry of Science and Technology of India, India |
| National Institute of Biomedical Genomics - NIBMG, India |
| University Grants Commission of India, India |
| Indonesian Institute of Sciences - LIPI, Indonesia |
| Irannian National Science Foundation, Iran |
| Ministry of Health and Medical Education of Iran, Iran |
| Government of Iraq, Iraq |
| Enterprise Ireland, Ireland |
| Health Research Board, Ireland |
| Healthy Ireland - Department of Health, Ireland |
| Irish Research Council, Ireland |
| Public Health Agency, Ireland |
| Government of Israel, Israel |
| Israel's Agency for International Development Cooperation - Mashav, Israel |
| Ministry of Health of Israel, Israel |
| Ministry of Science, Technology and Space of Israel, Israel |
| Agenzia Italiana del Farmaco, Italy |
| Italian National Institute of Health - ISS, Italy |
| Italian Space Agency - ASI, Italy |
| Lombardy Regional Government, Italy |
| Ministry of Economy and Finance of Italy, Italy |
| Ministry of Education, Universities and Research of Italy, Italy |
| Ministry of Foreign Affairs of Italy, Italy |
| Ministry of Health of Italy, Italy |
| Ministry of Health of Italy - National Centre for Disease Prevention and Control - CCM, Italy |
| National Institute for Social Security of Italy - INPS, Italy |
| National Research Council of Italy, Italy |
| Regional Government of Campania, Italy |
| Regional Government of Emilia-Romagna, Italy |
| Regional Government of Lazio, Italy |
| Regional Government of Liguria, Italy |
| Regional Government of Lombardy, Italy |
| Regional Government of Piedmont, Italy |
| Regional Government of Sardinia, Italy |
| Regional Government of Sicily, Italy |
| Regional Government of Tuscany, Italy |
| Regional Government of Veneto, Italy |
| Council for Science, Technology and Innovation - CSTI, Japan |
| Fukushima Prefectural Government, Japan |
| Government of Japan, Japan |
| Japan Agency for Medical Research and Development - AMED, Japan |
| Japan Science and Technology Agency - CREST, Japan |
| Japan Society for the Promotion of Science - JSPS, Japan |
| Ministry of Education, Culture, Sports, Science and Technology of Japan, Japan |
| Ministry of Health, Labour and Welfare of Japan, Japan |
| Ministry of the Environment of Japan, Japan |
| National Hospital Organization, Japan |
| New Energy and Industrial Technology Development Organization - NEDO, Japan |
| Shiga Prefectural Government, Japan |
| Yamanashi Government, Japan |
| Ministry of Education and Science of the Republic of Kazakhstan, Kazakhstan |
| National Cancer Institute of Kenya, Kenya |
| Latvian Council of Science, Latvia |
| National Council for Scientific Research of Lebanon, Lebanon |
| Lithuanian State Science and Studies Foundation, Lithuania |
| National Cancer Institute of Lithuania, Lithuania |
| Research Council of Lithuania, Lithuania |
| Ministry of Education of Malaysia, Malaysia |
| Ministry of Health of Malaysia, Malaysia |
| Ministry of Higher Education of Malaysia, Malaysia |
| Ministry of Science Technology and Innovation of Malaysia, Malaysia |
| Ministry of Science, Technology and Innovation of Malaysia, Malaysia |
| Ministry of Health, the Elderly and Community Care of Malta, Malta |
| Government of Mexico City, Mexico |
| Mexican Social Security Institute, Mexico |
| Mexico National Institute of Public Health, Mexico |
| National Cancer Institute of Mexico, Mexico |
| National Council for Science and Technology - CONACYT, Mexico |
| Agency for Innovation by Science and Technology - IWT, Netherlands |
| Dutch National Institute for Public Health and the Environment - RIVM, Netherlands |
| Dutch Technology Foundation - STW, Netherlands |
| Government of Rotterdam, Netherlands |
| Government of the Netherlands, Netherlands |
| Ministry of Health Welfare And Sport of the Netherlands, Netherlands |
| Ministry of the Interior and Kingdom Relations of the Netherlands, Netherlands |
| Netherlands Genomics Initiative, Netherlands |
| Royal Netherlands Academy of Arts and Sciences, Netherlands |
| Statistics Netherlands, Netherlands |
| The Netherlands Organisation for Health Research and Development - ZonMw, Netherlands |
| The Netherlands Organization for Scientific Research - NWO, Netherlands |
| Health Research Council of New Zealand, New Zealand |
| Central Norway Regional Health Authority, Norway |
| EEA Grants and Norway Grants, Norway |
| Ministry of Education and Research of Norway, Norway |
| Ministry of Health and Care Services of Norway, Norway |
| Northern Norway Regional Health Authority, Norway |
| Norwegian Agency for Development Cooperation, Norway |
| Norwegian Centre for International Cooperation in Education - SIU, Norway |
| Research Council of Norway, Norway |
| Southern and Eastern Norway Regional Health Authority, Norway |
| The Research Council of Norway, Norway |
| Western Norway Regional Health Authority, Norway |
| Government of Pakistan, Pakistan |
| Pakistan Atomic Energy Commission (PAEC), Pakistan |
| Ministry of Science and Higher Education of Poland, Poland |
| National Centre for Research and Development of Poland, Poland |
| National Committee for Scientific Research of Poland, Poland |
| National Science Centre of Poland, Poland |
| Foundation for Science and Technology - FCT, Portugal |
| Government of Portugal, Portugal |
| Executive Agency for Higher Education, Research, Development and Innovation Funding, Romania |
| Government of Romania, Romania |
| Ministry of National Education of Romania, Romania |
| Federal Agency for Scientific Organisations, Russia |
| Federal Agency for Scientific Organizations - FASO Russia, Russia |
| Government of Russia, Russia |
| Ministry of Science and Higher Education of the Russian Federation, Russia |
| Russian Academy of Sciences, Russia |
| Russian Foundation for Basic Research - RFRB, Russia |
| Russian Science Foundation - RSF, Russia |
| Government of Saudi Arabia, Saudi Arabia |
| King Abdulaziz City for Science and Technology - KACST, Saudi Arabia |
| Ministry of Health of Saudi Arabia, Saudi Arabia |
| Ministry of Education, Science and Technological Development of the Republic of Serbia, Serbia |
| Serbian Government, Serbia |
| Agency For Science Technology And Research of Singapore A Star, Singapore |
| Ministry of Education of Singapore, Singapore |
| Ministry of Health of Singapore, Singapore |
| National Medical Research Council of Singapore, Singapore |
| National Research Foundation of Singapore, Singapore |
| Ministry of Education, Science, Research and Sport of the Slovak Republic - Scientific Grant Agency, Slovakia |
| Scientific Grant Agency VEGA, Slovakia |
| Slovak Academy of Sciences, Slovakia |
| Ministry of Education, Science and Sport of the Republic of Slovenia, Slovenia |
| Ministry of Health of the Republic of Slovenia, Slovenia |
| Department of Science and Technology of South Africa, South Africa |
| Medical Research Council of South Africa, South Africa |
| National Research Foundation of South Africa, South Africa |
| Government of South Korea, South Korea |
| Korea Centers for Disease Control and Prevention, South Korea |
| Korea Health Industry Development Institute - KHIDI, South Korea |
| Korea Research Foundation, South Korea |
| Korea Science and Engineering Foundation, South Korea |
| Ministry of Agriculture, Food and Rural Affairs of South Korea, South Korea |
| Ministry of Education, Science and Technology of the Republic of Korea, South Korea |
| Ministry of Food and Drug Safety of the Republic of Korea, South Korea |
| Ministry of Health and Welfare of the Republic of Korea, South Korea |
| Ministry of Oceans and Fisheries of the Republic of Korea, South Korea |
| Ministry of Trade, Industry and Energy of the Republic of Korea, South Korea |
| National Research Foundation of Korea, South Korea |
| Nuclear Safety and Security Commission, South Korea |
| Seoul Metropolitan Government, South Korea |
| Albacete Municipal Government, Spain |
| Basque Country Government, Spain |
| Foundation for the Promotion of Applied Scientific Research and Technology in Asturias - FICYT, Spain |
| Government of Spain, Spain |
| Health Institute Carlos III, Spain |
| Madrid Municipal Government, Spain |
| Ministry of Economy, Industry and Competitiveness of Spain, Spain |
| Ministry of Education, Culture and Sport of Spain, Spain |
| Ministry of Health of Spain, Spain |
| Ministry of Health, Social Services and Equality of Spain, Spain |
| Ministry of Science and Innovation of Spain, Spain |
| Regional Government of Andalusia, Spain |
| Regional Government of Aragon, Spain |
| Regional Government of Asturias, Spain |
| Regional Government of Basque Country, Spain |
| Regional Government of Castile and Leon, Spain |
| Regional Government of Castile-La Mancha, Spain |
| Regional Government of Catalonia, Spain |
| Regional Government of Catalonia - Agency For Management of University of Research Grants - AGAUR, Spain |
| Regional Government of Galicia, Spain |
| Regional Government of Murcia, Spain |
| Regional Government of Navarre, Spain |
| Regional Government of Valencia, Spain |
| Spanish National Research Council - CSIC, Spain |
| Ministry of Health, Nutrition and Indigenous Medicine of Sri Lanka, Sri Lanka |
| Government of Sweden, Sweden |
| Örebro County Council, Sweden |
| Östergötland County, Sweden |
| Skåne Regional Council, Sweden |
| Stockholm Municipal Government, Sweden |
| Swedish Institute, Sweden |
| Swedish National Board of Health and Welfare, Sweden |
| Swedish Radiation Safety Authority, Sweden |
| Swedish Research Council, Sweden |
| Swedish Research Council for Health, Working Life and Welfare - FORTE, Sweden |
| The Swedish Research Council for Sustainable Development - Formas, Sweden |
| Uppsala Regional Council, Sweden |
| Västerbotten Regional Council, Sweden |
| Västra Götaland Regional Council, Sweden |
| Vinnova, Sweden |
| Federal Department of Economic Affairs, Education and Research, Switzerland |
| Government Council of the Canton of Zurich, Switzerland |
| Swiss Federal Office of Public Health, Switzerland |
| Swiss State Secretariat for Education, Research and Innovation - SERI, Switzerland |
| Government of Taiwan, Taiwan |
| Ministry of Economic Affairs of Taiwan, Taiwan |
| Ministry of Education of Taiwan, Taiwan |
| Ministry of Health and Welfare of Taiwan, Taiwan |
| Ministry of Science and Technology of Taiwan, Taiwan |
| National Health Research Institutes of Taiwan, Taiwan |
| Government of Thailand, Thailand |
| Ministry of Education of Thailand, Thailand |
| National Cancer Institute of Thailand, Thailand |
| National Research Council of Thailand, Thailand |
| National Science and Technology Development Agency - NSTDA, Thailand |
| Thailand National Research Universities, Thailand |
| Thailand Research Fund, Thailand |
| Ministry of Higher Education and Scientific Research of Tunisia, Tunisia |
| Ministry of Health of Turkey, Turkey |
| Scientific and Technological Research Council of Turkey - TÜBITAK, Turkey |
| Turkish Academy of Sciences, Turkey |
| National Academy of Sciences of Ukraine, Ukraine |
| Biotechnology And Biological Sciences Research Council, United Kingdom |
| Department of International Development, United Kingdom |
| Economic and Social Research Council, United Kingdom |
| Engineering and Physical Sciences Research Council, United Kingdom |
| Food Standards Agency, United Kingdom |
| Governent of Scotland, United Kingdom |
| Health and Care Research Wales, United Kingdom |
| Higher Education Funding Council for England, United Kingdom |
| Natural Environment Research Council, United Kingdom |
| Public Health England, United Kingdom |
| Research Councils UK, United Kingdom |
| Scottish Funding Council, United Kingdom |
| Scottish Government Health Directorates - Chief Scientist Office, United Kingdom |
| The Academy of Medical Sciences, United Kingdom |
| UK Department of Health, United Kingdom |
| UK Health and Safety Executive, United Kingdom |
| UK Medical Research Council, United Kingdom |
| UK National Health Service - NHS, United Kingdom |
| UK National Institute for Health Research, United Kingdom |
| UK National Institute for Health Research - NIHR, United Kingdom |
| UK Office for Nuclear Regulation - ONR, United Kingdom |
| Welsh Government, United Kingdom |
| National Agency for Research and Innovation in Uruguay - ANII, Uruguay |
| Agency for Healthcare Research and Quality, USA |
| Alabama State Government, USA |
| Arizona Government, USA |
| California Institute For Regenerative Medicine, USA |
| Center of Disease Control and Prevention - National Institute for Occupational Safety and Health - NIOSH, USA |
| Centers For Disease Control And Prevention - CDC, USA |
| Connecticut Government, USA |
| Defense Advanced Research Projects Agency - DARPA, USA |
| Florida State Government, USA |
| Food and Drug Administration - FDA, USA |
| Government of Colorado State, USA |
| Government of Illinois, USA |
| Government of Kansas State, USA |
| Government of the State of Louisiana, USA |
| Hawaii State Government, USA |
| Health Resources and Services Administration - HRSA, USA |
| Maryland State Government, USA |
| Minnesota State Government, USA |
| National Academy of Sciences of Hungary, USA |
| National Aeronautics and Space Administration - NASA, USA |
| National Institute on Drug Abuse - NIDA, USA |
| National Institutes of Health - Eunice Kennedy Shriver National Institute of Child Health and Human Development - NICHD, USA |
| National Institutes of Health - John E. Fogarty International Center, USA |
| National Institutes of Health - National Cancer Institute - NCI, USA |
| National Institutes of Health - National Cencer for Biotechnology Information, USA |
| National Institutes of Health - National Center for Advancing Translational Sciences - NCATS, USA |
| National Institutes of Health - National Center for Complementary and Integrative Health - NCCAM, USA |
| National Institutes of Health - National Center for Research Resources - NCRR, USA |
| National Institutes of Health - National Eye Institute - NEI, USA |
| National Institutes of Health - National Heart, Lung, and Blood Institute - NHLBI, USA |
| National Institutes of Health - National Human Genome Research Institute - NHGRI, USA |
| National Institutes of Health - National Institute of Aging - NIA, USA |
| National Institutes of Health - National Institute of Allergy and Infectious Diseases - NIAID, USA |
| National Institutes of Health - National Institute of Arthritis and Musculoskeletal and Skin Diseases - NIAMS, USA |
| National Institutes of Health - National Institute of Biomedical Imaging and Bioengineering - NIBIB, USA |
| National Institutes of Health - National Institute of Dental and Craniofacial Research - NIDCR, USA |
| National Institutes of Health - National Institute of Diabetes and Digestive and Kidney Diseases - NIDDK, USA |
| National Institutes of Health - National Institute of Environmental Health Sciences - NIEHS, USA |
| National Institutes of Health - National Institute of General Medical Sciences - NIGMS, USA |
| National Institutes of Health - National Institute of Mental Health - NIMH, USA |
| National Institutes of Health - National Institute of Neurological Disorders and Stroke - NINDS, USA |
| National Institutes of Health - National Institute of Nursing Research - NINR, USA |
| National Institutes of Health - National Institute on Alcohol Abuse and Alcoholism, USA |
| National Institutes of Health - National Institute on Alcohol Abuse and Alcoholism - NIAAA, USA |
| National Institutes of Health - National Institute on Deafness and Other Communication Disorders - NIDCD, USA |
| National Institutes of Health - National Institute on Minority Health and Health Disparities - NIMHD, USA |
| National Institutes of Health - National Library of Medicine, USA |
| National Institutes of Health - NIH, USA |
| National Institutes of Health - Office of Dietary Supplement - ODS, USA |
| National Institutes of Health - Office of Research on Women's Health - ORWH, USA |
| National Palliative Care Research Center, USA |
| Nebraska State Government, USA |
| New-York State Government, USA |
| North Carolina State Government, USA |
| Oklahoma Center for the Advancement of Science and Technology, USA |
| Pennsylvania State Government, USA |
| State of Arkansas Government, USA |
| State of California Government, USA |
| State of California Government - California Breast Cancer Research Program, USA |
| State of California Government - California Tobacco Related Disease Research Program, USA |
| State of Colorado Government, USA |
| State of Connecticut Government, USA |
| State of Florida Government, USA |
| State of Illinois Government, USA |
| State of Iowa Government, USA |
| State of Maryland Government, USA |
| State of New Jersey Government, USA |
| State of New Mexico Government, USA |
| State of Tennessee Government, USA |
| State of Texas Government, USA |
| State of Utah Government, USA |
| State of Washington Government, USA |
| State of West Virginia Government, USA |
| Texas AgriLife Research, USA |
| United States Air Force Materiel Command, USA |
| United States Postal Service - USPS, USA |
| US Agency for International Development, USA |
| US Department of Agriculture, USA |
| US Department of Defense - Congressionally Directed Medical Research Programs - CDMRP, USA |
| US Department of Defense - DOD, USA |
| US Department of Defense - Office of Naval Research - ONR, USA |
| US Department of Defense - United States Army, USA |
| US Department of Education, USA |
| US Department of Energy, USA |
| US Department of Energy - Office of Science, USA |
| US Department of Health and Human Services - HHS, USA |
| US Department of State - Bureau of Educational and Cultural Affairs, USA |
| US Department of Veterans Affairs, USA |
| US National Science Foundation - NSF, USA |
| National Foundation for Science and Technology of Vietnam - Nafosted, Vietnam |

**International/Intergovernmental organizations**

| International Atomic Energy Agency, Austria |
| --- |
| Biobanking And Biomolecular Resources Research Infrastructure - BBMRI-ERIC, Belgium |
| European Commission - Consumers, Health, Agriculture and Food Executive Agency, Belgium |
| European Commission - Education and Culture, Belgium |
| European Commission - Employment, Social Affairs and Inclusion, Belgium |
| European Commission - European Partnership for Action Against Cancer, Belgium |
| European Commission - European Regional Development Fund - FEDER, Belgium |
| European Commission - European Social Fund, Belgium |
| European Commission - Health and Food Safety, Belgium |
| European Commission - International Cooperation and Development - COST, Belgium |
| European Commission - Joint Research Centre, Belgium |
| European Commission - Research and Innovation, Belgium |
| European Commission - Research Executive Agency, Belgium |
| European Commission - TRANSCAN - ERA-Net, Belgium |
| European Research Council, Belgium |
| World Health Organization - Regional Office for Africa, Congo |
| World Health Organization - Regional Office for Europe, Denmark |
| World Health Organization - Regional Office for the Eastern Mediterranean, Egypt |
| European Clinical Research Infrastructure Network - ECRIN, France |
| International Agency for Research on Cancer - IARC, France |
| International Human Frontier Science Program Organization, France |
| European Molecular Biology Organization - EMBO, Germany |
| World Health Organization - Regional Office for South East Asia, India |
| European Commission - European Food Safety Authority, Italy |
| International Centre for Genetic Engineering and Biotechnology, Italy |
| African Development Bank Group, Ivory Coast |
| Arab Fund For Economic And Social Development, Kuwait |
| NordForsk, Norway |
| Asian Development Bank, Philippines |
| World Health Organization - Regional Office for the Western Pacific, Philippines |
| Arab Gulf Program For Development, Saudi Arabia |
| Islamic Development Bank, Saudi Arabia |
| Joint UN Programme on HIV/AIDS, Switzerland |
| World Health Organization - Headquaters, Switzerland |
| East, Central and Southern Africa Health Community, Tanzania |
| Science and Technology Center in Ukraine - STCU, Ukraine |
| European Commission - Innovative Medicines Initiative, United Kingdom |
| Inter-American Development Bank, USA |
| United Nations - United Nations Educational, Scientific and Cultural Organization, USA |
| United Nations - United Nations Population Fund, USA |
| World Bank, USA |
| World Health Organization - Regional Office for the Americas, USA |

**Not-for-profit**

| Afghan Society Against Cancer, Afghanistan |
| --- |
| Crèdit Andorrà Foundation, Andorra |
| A. J. Roemmers Foundation, Argentina |
| Bunge y Born Foundation, Argentina |
| Florencio Fiorini Foundation, Argentina |
| Liga Argentina de Lucha Contra el Cáncer, Argentina |
| René Baron Foundation, Argentina |
| SALES Foundation, Argentina |
| Anthony Rothe Memorial Trust, Australia |
| ANZAC Research Institute - ANZAC Health and Medical Research Foundation, Australia |
| Arrow Bone Marrow Transplant Foundation, Australia |
| auDA Foundation, Australia |
| Austin Medical Research Foundation, Australia |
| Australasian Gastro-Intestinal Trials Group, Australia |
| Australasian Leukaemia and Lymphoma Group, Australia |
| Australasian Sarcoma Study Group, Australia |
| Australia and New Zealand Melanoma Trials Group, Australia |
| Australia New Zealand Gynaecological Oncology Group, Australia |
| Australian and New Zealand Children's Haematology and Oncology Group, Australia |
| Australian and New Zealand Urogenital and Prostate Cancer Trials Group, Australia |
| Australian Cancer Research Foundation, Australia |
| Australian Dental Research Foundation, Australia |
| Australian Lions Childhood Cancer Research Foundation, Australia |
| Australian Rotary Health, Australia |
| Avner Pancreatic Cancer Foundation, Australia |
| Beyond Blue Foundation, Australia |
| Brainchild Foundation, Australia |
| Breast Cancer Trials, Australia |
| Bupa Health Foundation, Australia |
| Cancer Council Act, Australia |
| Cancer Council Australia, Australia |
| Cancer Council New South Wales, Australia |
| Cancer Council Northern Territory, Australia |
| Cancer Council Queensland, Australia |
| Cancer Council South Australia, Australia |
| Cancer Council Tasmania, Australia |
| Cancer Council Victoria, Australia |
| Cancer Council Western Australia, Australia |
| Cancer Nurses Society of Australia, Australia |
| CanTeen, Australia |
| Cass Foundation, Australia |
| Charlies Foundation for Research, Australia |
| Children’s Hospital Foundation, Australia |
| Children's Cancer Foundation, Australia |
| Children's Leukaemia and Cancer Research Foundation, Australia |
| Clinical Oncology Society of Australia, Australia |
| Colonial Foundation, Australia |
| Cooperative Trials Group for Neuro-Oncology, Australia |
| Cure Brain Cancer Foundation, Australia |
| Cure Cancer Australia, Australia |
| Cure The Future, Australia |
| Diabetes Australia Research Program, Australia |
| Epworth Medical Foundation, Australia |
| Equity Trustees, Australia |
| Fay Fuller Foundation, Australia |
| Fight Cancer Foundation, Australia |
| Flannery Family Foundation, Australia |
| Foundation for Alcohol Research and Education, Australia |
| Garvan Research Foundation, Australia |
| Helen MacPherson Smith Trust, Australia |
| Hunter Cancer Research Alliance, Australia |
| Huntsman Cancer Institute - Huntsman Cancer Foundation, Australia |
| Ian Potter Foundation, Australia |
| Jack and Robert Smorgon Families Foundation - ESCOR, Australia |
| Keith Boden Fellowship, Australia |
| Kidney Health Australia, Australia |
| Kids with Cancer Foundation, Australia |
| Kolling Foundation, Australia |
| Leukaemia Foundation of Australia, Australia |
| Leukemia Foundation, Australia |
| Lishman Health Foundation, Australia |
| Little Heroes Foundation, Australia |
| Lorenzo and Pamela Galli Charitable Trust, Australia |
| Lung Foundation Australia, Australia |
| Lymphoma Australia, Australia |
| Macquarie Group Foundation, Australia |
| Medical Oncology of Group of Australia, Australia |
| Minderoo Foundation, Australia |
| Movember Foundation, Australia |
| Multiple Sclerosis Research Australia, Australia |
| Myeloma Foundation of Australia Inc., Australia |
| National Breast Cancer Foundation, Australia |
| National Foundation for Medical Research and Innovation, Australia |
| Olivia Newton-John Cancer Research Institute, Australia |
| Ovarian Cancer Australia, Australia |
| PA Research Foundation, Australia |
| Pancare Foundation, Australia |
| Patricia Helen Guest fellowship, Australia |
| Peter MacCallum Cancer Foundation, Australia |
| Petre Foundation, Australia |
| Ponting Foundation, Australia |
| Prostate Cancer Foundation of Australia, Australia |
| Rainbows for Kate Foundation, Australia |
| Raine Medical Research Foundation, Australia |
| Research for Life - Wellington Medical Research Foundation, Australia |
| Royal Adelaide Hospital Research Fund, Australia |
| Royal Australasian College of Physicians, Australia |
| Royal Australasian College of Surgeons, Australia |
| Royal Australian and New Zealand College of Radiologists, Australia |
| Royal Brisbane and Women's Hospital Foundation, Australia |
| Royal Hobart Hospital Research Foundation, Australia |
| Royal Perth Hospital Medical Research Foundation, Australia |
| Sidney Myer Fund and The Myer Foundation, Australia |
| Sir Edward Dunlop Medical Research Foundation, Australia |
| Skin and Cancer Foundation Inc., Australia |
| Sydney Breast Cancer Foundation, Australia |
| Sydney Children's Hospital Foundation, Australia |
| Sydney Medical School Foundation, Australia |
| Tasmania's Clifford Craig Medical Research Trust, Australia |
| Telethon Kids Institute, Australia |
| Telstra Foundation, Australia |
| The Alfred Foundation, Australia |
| The Friends of the Mater Foundation, Australia |
| The Hospital Research Foundation, Australia |
| The Kids' Cancer Project, Australia |
| The Mostyn Family Foundation, Australia |
| The Pratt Foundation, Australia |
| The Ride To Conquer Cancer, Australia |
| The Royal Children's Hospital Foundation, Australia |
| The Royal Melbourne Hospital Foundation, Australia |
| The Royal Melbourne Hospital Neuroscience Foundation, Australia |
| The Royal Women's Hospital Foundation, Australia |
| The University of Syndney - Melanoma Foundation, Australia |
| The Weekend to End Women's Cancers, Australia |
| Trans-Tasman Radiation Oncology Group, Australia |
| Urological Society of Australia and New Zealand, Australia |
| Victorian Health Promotion Foundation - VicHealth, Australia |
| Victorian Prostate Cancer Research Consortium, Australia |
| Wesley Medical Research, Australia |
| Westmead Institute for Medical Research, Australia |
| Austrian Cancer Aid, Austria |
| Cancer Epigenetics Society, Austria |
| European Academy of Allergy and Clinical Immunology - EAACI, Austria |
| Fellinger Cancer Research - Fellinger Krebsforschung, Austria |
| Medical Research Fund Tyrol - MFF Tirol, Austria |
| The Herzfelder Family Foundation, Austria |
| United European Gastroenterology - UEG, Austria |
| Bahrain Cancer Society, Bahrain |
| Eminence, Bangladesh |
| Obayedullah-Ferdousi Foundation Cancer Hospital and Research Institute - POFF, Bangladesh |
| Oncology Club Bangladesh, Bangladesh |
| Anticancerfund, Belgium |
| Belgian Foundation Against Cancer, Belgium |
| Children's Cancer Fund, Belgium |
| European Breast Cancer Council, Belgium |
| European Cancer Organisation - ECCO, Belgium |
| European Federation of Pharmaceutical Industries and Associations - EFPIA, Belgium |
| European Organisation for Research and Treatment of Cancer - EORTC, Belgium |
| European Society for Radiotherapy and Oncology - ESTRO, Belgium |
| European Society of Surgical Oncology - ESSO, Belgium |
| Flemish League against Cancer, Belgium |
| Fondation Saint-Luc, Belgium |
| Fonds de la Recherche Scientifique - FRS-FNRS, Belgium |
| Fonds Erasme pour la recherche medicale, Belgium |
| Fonds Joseph Maisin, Belgium |
| Fonds Léon Fredericq, Belgium |
| Fonds Yvonne Boël, Belgium |
| King Baudouin Foundation, Belgium |
| Les Amis de l'Institut Bordet, Belgium |
| Limburgs Cancer Foundation, Belgium |
| Mobile and Wireless Forum, Belgium |
| Myny-Vanderpoorten Foundation, Belgium |
| Olivia Hendrickx Research Fund, Belgium |
| Research Foundation Flanders - FWO, Belgium |
| Stand up Against Cancer - Kom op tegen Kanker, Belgium |
| The European Hematology Association, Belgium |
| Brazilian Cancer Foundation, Brazil |
| Brazilian Lymphoma and Leukaemia Association - ABRALE, Brazil |
| Brazilian Society of Surgical Oncology - BSSO, Brazil |
| Farmabrasilis, Brazil |
| Latin American Cooperative Oncology Group - LACOG, Brazil |
| Paraná State Foundation, Brazil |
| State of Minas Gerais Research Foundation - FAPEMIG, Brazil |
| State of Rio de Janeiro State Research Foundation - FAPERJ, Brazil |
| State of RS State Research Foundation - FAPERGS, Brazil |
| State of Sao Paulo Research Foundation - FAPESP, Brazil |
| Action contre le Cancer Infantile au Burkina Faso, Burkina Faso |
| Alliance Burundaise Contre Le Cancer, Burundi |
| 7 Days In May Foundation, Canada |
| Alberta Children's Hospital Foundation, Canada |
| Alberta Heritage Foundation For Medical Research, Canada |
| Allard Foundation, Canada |
| BC Cancer Foundation, Canada |
| Bladder Cancer Canada, Canada |
| Brain Canada, Canada |
| Brain Tumour Foundation of Canada, Canada |
| Breast Cancer Society of Canada, Canada |
| British Columbia Cancer Foundation, Canada |
| B-Strong, Canada |
| Canada Foundation For Innovation, Canada |
| Canadian Association of Radiation Oncology, Canada |
| Canadian Breast Cancer Foundation, Canada |
| Canadian Breast Cancer Research Alliance, Canada |
| Canadian Cancer Research Society, Canada |
| Canadian Cancer Society, Canada |
| Canadian Dermatology Foundation, Canada |
| Canadian Federation for Sexual Health, Canada |
| Canadian Liver Foundation, Canada |
| Canadian Tumour Repository Network, Canada |
| Canadian Wireless Telecommunications Association, Canada |
| Cancercare Manitoba Foundation, Canada |
| Carcinoid Neuroendocrine Tumour Society of Canada, Canada |
| Cedars Cancer Foundation, Canada |
| Childhood Cancer Canada Foundation, Canada |
| Children's Cancer And Blood Disorder - C17, Canada |
| CHU Sainte-Justine Foundation, Canada |
| Colorectal Cancer Canada, Canada |
| Cure Foundation, Canada |
| Dalhousie Medical Research Foundation, Canada |
| Diabetes Canada - Canadian Diabetes Association, Canada |
| Emmanuelle Gattuso Foundation, Canada |
| Fondation Armand-Frappier, Canada |
| Garron Family Foundation, Canada |
| Guzzo Environment-Cancer Research Chair, Canada |
| Hamilton Health Sciences Foundation, Canada |
| Health Research Foundation, Canada |
| Heart and Stroke Foundation of Canada, Canada |
| Hope and Cope, Canada |
| International Society of Nurses in Cancer Care - ISNCC, Canada |
| Izaak Walton Killam Memorial Scholarship, Canada |
| Joseph and Silvana Melara Cancer Research Fund, Canada |
| Kids Cancer Care, Canada |
| Killam Trusts, Canada |
| Krembil Foundation, Canada |
| Leukemia and Lymphoma Society of Canada, Canada |
| Lotte and John Hecht Memorial Foundation, Canada |
| Lymphoma Canada, Canada |
| Mitacs Canada, Canada |
| Myeloma Canada, Canada |
| Northern Cancer Foundation, Canada |
| Nova Scotia Health Research Foundation, Canada |
| Ottawa Regional Cancer Foundation, Canada |
| Ovarian Cancer Canada, Canada |
| Pancreatic Cancer Canada, Canada |
| Pediatric Oncology Group of Ontario - POGO, Canada |
| Princess Margaret Cancer Foundation, Canada |
| Prostate Cancer Canada, Canada |
| Prostate Cancer Fight Foundation, Canada |
| Royal College of Physicians and Surgeons of Canada, Canada |
| Run For Ovarian Cancer, Canada |
| SickKids Foundation, Canada |
| Teresa Cascioli Charitable Foundation, Canada |
| The Cole Foundation, Canada |
| The James Fund, Canada |
| The Kidney Foundation of Canada, Canada |
| The Montreal Children's Hospital Foundation, Canada |
| The Montreal General Hospital Foundation, Canada |
| The Ottawa Hospital Foundation, Canada |
| The Quebec Breast Cancer Foundation, Canada |
| The Rudolph P. Bratty Family Foundation, Canada |
| The Slaight Family Foundation, Canada |
| The Terry Fox Foundation, Canada |
| Université de Montréal Foundation, Canada |
| VGH and UBC Hospital Foundation, Canada |
| Young Adult Cancer Canada, Canada |
| Arturo López Pérez Foundation, Chile |
| Albert Hung Foundation, China |
| Asian Fund For Cancer Research, China |
| Cancer Chile Foundation, China |
| Cancer Foundation of China, China |
| China Foundation for Hepatitis Prevention and Control, China |
| China Health Promotion Foundation, China |
| China International Medical Foundation, China |
| China Medical Board, China |
| China Postdoctoral Science Foundation, China |
| Chinese Anti-Cancer Association, China |
| Chinese Medical Association, China |
| Chinese Society of Clinical Oncology - CSCO, China |
| Chinese Society of Neuro-oncology, China |
| Dr Ellen Li Charitable Foundation, China |
| Fok Ying Tong Education Foundation, China |
| Fong Shu Fook Tong Foundation and Fong's Family Foundation, China |
| Henan Anti-Cancer Association, China |
| Hong Kong Anti-Cancer Society, China |
| Hong-Kong Cancer Fund, China |
| Hong-Kong Hereditary Breast Cancer Family Registry, China |
| Hong-Kong Jockey Club Charities Trust, China |
| K.C. Wong Magna Fund in Ningbo University, China |
| KC Wong Education Foundation, China |
| Lee Shiu Family Foundation Limited, China |
| Li Ka Shing Foundation, China |
| PKU Lam Chung-nin Fund for Systems Biomedicine, China |
| S. K. Yee Medical Foundation, China |
| Shanghai Charity Foundation, China |
| Wong Check She Charitable Foundation, China |
| Wu Jie Ping Medical Foundation, China |
| Colombian League Against Cancer, Colombia |
| Foundation for Clinical and Applied Cancer Research - FICMAC, Colombia |
| Agir Ensemble, Congo |
| Croatian Science Foundation, Croatia |
| Cuban Society of Oncology, Radiotherapy and Nuclear Medicine, Cuba |
| Prague League Against Cancer, Czech Republic |
| The Czech Science Foundation, Czech Republic |
| The Kellner Family Foundation, Czech Republic |
| Aage And Johanne Louis Hansen Fund, Denmark |
| Aarhus University Research Foundation, Denmark |
| Aase And Ejnar Danielsen Foundation, Denmark |
| Agnes And Poul Friis Fund, Denmark |
| AP Moller Foundation, Denmark |
| Arvid Nilssons Fond, Denmark |
| Augustinus Foundation, Denmark |
| Carlsberg Foundation, Denmark |
| Dagmar Marshall Fund, Denmark |
| Danish Cancer Society, Denmark |
| Danish Children's Cancer Association, Denmark |
| Danish Diabetes Academy, Denmark |
| Danish National Research Foundation, Denmark |
| E. and M. Wedell-Wedellsborgs Foundation, Denmark |
| Harbo Fund, Denmark |
| Herlev Hospital Foundation, Denmark |
| I.M. Daehnfeldt Foundation, Denmark |
| IMK General Fund, Denmark |
| Innovation Fund Denmark, Denmark |
| Jacob and Olga Madsen Foundation, Denmark |
| Johan and Lise Boserup Fund, Denmark |
| John and Birthe Meyer Foundation, Denmark |
| Karen A. Tolstrup Foundation, Denmark |
| Karen Elise Jensen's Foundation, Denmark |
| Kirsten and Freddy Johansen Foundation, Denmark |
| Krista and Viggo Petersen Foundation, Denmark |
| Marie and Børge Kroghs Foundation, Denmark |
| Neye Foundation, Denmark |
| Novo Nordisk Foundation, Denmark |
| Otto Christensen Foundation, Denmark |
| Sophus Jacobsen and wife Astrid Jacobsen's Foundation, Denmark |
| Svend Andersen Foundation, Denmark |
| Synoptik Foundation, Denmark |
| The A.P. Møller and Chastine Mc-Kinney Møller Foundation, Denmark |
| The Augustinus Foundation, Denmark |
| The Danish Heart Foundation, Denmark |
| The Gangsted Foundation, Denmark |
| The Health Foundation - Helsefonden, Denmark |
| The Hede Nielsen Family Foundation, Denmark |
| The Lundbeck Foundation, Denmark |
| The Obel Family Foundation, Denmark |
| The VELUX Foundations - Villum Foundation, Denmark |
| The Walter and O. Kristiane Christensen Fund, Denmark |
| Tryg Foundation, Denmark |
| Riisfort Foundation, Denmark |
| Galo Plaza Lasso Foundation, Ecuador |
| Salvadoran Association for the Prevention of Cancer - ASAPRECAN, El Salvador |
| Biomedicum Helsinki Foundation, Finland |
| Cancer Society of Finland, Finland |
| Emil Aaltonen Foundation, Finland |
| Finnish Anti-Tuberculosis Association, Finland |
| Finnish Cancer Society, Finland |
| Finnish Cultural Foundation, Finland |
| Finnish Dental Society Apollonia, Finland |
| Finnish Foundation for Cardiovascular Research, Finland |
| Finnish Medical Foundation, Finland |
| Finnish Work Environment Fund, Finland |
| Finnish-Norwegian Medical Foundation, Finland |
| Foundation for Pediatric Research , Finland |
| Heikki, Aino and Aarne Korhonen Foundation, Finland |
| Ida Montinin Foundation, Finland |
| Instrumentarium Science Foundation, Finland |
| Jalmari and Rauha Ahokas Foundation, Finland |
| Jane and Aatos Erkko Foundation, Finland |
| K. Albin Johanssons Foundation, Finland |
| Magnus Ehrnrooth Foundation, Finland |
| Mary and Georg C. Ehrnrooths Foundation, Finland |
| Maud Kuistila Memorial Foundation, Finland |
| Orion-Farmos Research Foundation, Finland |
| Otto A. Malm Foundation, Finland |
| Paavo Koistinen Foundation, Finland |
| Päivikki and Sakari Sohlberg Foundation, Finland |
| Ruth and Nils-Erik Stenbäck Foundation, Finland |
| Seppo Nieminen Fund, Finland |
| Signe and Ane Gyllenberg Foundation, Finland |
| Sigrid Jusélius Foundation, Finland |
| The Finnish Medical Society Duodecim, Finland |
| The Paulo Foundation, Finland |
| The Reino Lahtikari Foundation, Finland |
| Thelma Mäkikyrö Foundation, Finland |
| Tuberculosis Foundation Tampere, Finland |
| Turku University Foundation, Finland |
| Väre Foundation for Pediatric Cancer Research, Finland |
| Yrjö Jahnsson Foundation, Finland |
| Afrocancer, France |
| Alliance Mondiale contre le Cancer - ALIAM, France |
| Alliance pour les Sciences de la Vie et de la Santé - Institut Thématique Multi-Organisme Cancer - ITMO Cancer, France |
| Association Anne De Bretagne Genetique, France |
| Association Cent Pour Sang La Vie, France |
| Association DAM'S, France |
| Association Française Contre Les Myopathies - AFM, France |
| Association Française D'Urologie, France |
| Association Guillaume Espoir, France |
| Association Hubert Gouin - Enfance and Cancer, France |
| Association Laurette Fugain, France |
| Association pour la Recherche sur les Tumeurs Cérébrales - ARTC, France |
| Association pour la Recherche sur les Tumeurs de la Prostate, France |
| Association Vaincre le mélanome, France |
| Bettencourt Schueller Foundation, France |
| Enfants Cancers Santé, France |
| Fédération Francophone de Cancérologie Digestive, France |
| Fédération GEFLUC, France |
| FONCER contre le cancer, France |
| Fondation Allianz, France |
| Fondation Bullukian, France |
| Fondation de France, France |
| Fondation de l'avenir, France |
| Fondation Francphone pour la Recheche sur le Diabète, France |
| Fondation Innovations en Infectiologie - FINOVI, France |
| Fondation Leducq, France |
| Fondation Maladies Rares, France |
| Fondation Martine Midy, France |
| Fondation Medic, France |
| Fondation Mérieux, France |
| Fondation MGen pour la Santé Publique, France |
| Fondation pour l’Université de Lyon, France |
| Fondation pour la Recherche en alcoologie, France |
| Fondation pour la Recherche Médicale, France |
| Fondation pour la Recherche sur le Cancer - ARC, France |
| Fondation René Touraine, France |
| Fondation Toulouse Cancer Santé, France |
| Fondation Tourre, France |
| Fonds AXA pour la recherche, France |
| Fonds de Recherche en Santé Respiratoire, France |
| Foster Research in Molecular Biology, France |
| France Lymphome Espoir, France |
| Groupe Pasteur Mutualité, France |
| Institut Olga Triballat, France |
| Institute Danone, France |
| L’Oréal Foundation, France |
| La Fondation du souffle, France |
| La Ligue Nationale contre le Cancer - French League Against Cancer - LNCC, France |
| Le Cancer du Sein, Parlons-en!, France |
| LEEM Recherche, France |
| Les Bagouz' à Manon, France |
| L'étoile de Martin, France |
| Open Health Institute, France |
| Sanofi Espoir Foundation, France |
| Sidaction, France |
| Société Française de Dermatologie, France |
| Société française de lutte contre les cancers et les leucémies de l'enfant et de l'adolescent - SFCE, France |
| Société Française de Nutrition, France |
| Sociéte Française d'hématologie - SFH, France |
| Société Nationale Française de Gastro-Entérologie, France |
| The L'Oréal-UNESCO For Women in Science Initiative, France |
| The Lymphoma Study Association, France |
| Association Cassandra, France |
| Alexander Von Humboldt Foundation, Germany |
| B. Braun Foundation, Germany |
| Baden-Württemberg Foundation , Germany |
| Banss Foundation, Germany |
| Bayimmunet, Germany |
| Bettina Bräu Foundation, Germany |
| Boehringen Ingelheim Fonds, Germany |
| Childhood Cancer Association in Hannover, Germany |
| Childhood Cancer Initiative - KKI, Germany |
| Cura Placida - Children's Cancer Research Foundation, Germany |
| Daimler and Benz Foundation, Germany |
| Dieter Schlag Foundation, Germany |
| Dietmar Hopp Foundation, Germany |
| Dr Hella-Bühler-Foundation, Germany |
| Dr Ingrid zu Solms Foundation, Germany |
| Dr Mildred Scheel Foundation For Cancer Research, Germany |
| Dr Robert Pfleger Foundation, Germany |
| Dr Senckenberg Foundation, Germany |
| Dr Werner Jackstädt Foundation, Germany |
| Effective Altruism Foundation, Germany |
| Else Kröner-Fresenius Foundation, Germany |
| Erich and Gertrud Roggenbuck Foundation, Germany |
| Ernst Jung Foundation, Germany |
| European Foundation for the Study of Diabetes, Germany |
| Falk Foundation, Germany |
| Frankfurt Children with Cancer Foundation, Germany |
| Friedrich Baur Foundation, Germany |
| Friends of the Hannover Medical School, Germany |
| Fritz Bender Foundation, Germany |
| Fritz-Thyssen Foundation, Germany |
| Gänseblümchen-Voerde Foundation, Germany |
| German Academic Scholarship Foundation, Germany |
| German Cancer Aid - Deutsche Krebshilfe, Germany |
| German Cancer Society, Germany |
| German Childhood Cancer Foundation - Deutsche Kinderkrebsstiftung, Germany |
| German Research Foundation - DFG, Germany |
| German Society for Allergology and Clinical Immunology - DGAKI, Germany |
| German Society of Urology - DGU, Germany |
| German-Israeli Foundation for Scientific Research and Development, Germany |
| German-Israeli Helmholtz Research School in Cancer Biology, Germany |
| Hanns A. Pielenz Foundation, Germany |
| Hans Sauer Foundation, Germany |
| Hertie Foundation, Germany |
| Hiege Foundation Against Skin Cancer, Germany |
| Jürgen Manchot Foundation, Germany |
| Kind Philipp Foundation for Pediatric Oncology Research, Germany |
| Krebsallianz GmbH, Germany |
| Lower Saxonian Cancer Society, Germany |
| Madeleine Schickedanz Children's Cancer Foundation, Germany |
| Margarete Bonifer Foundation, Germany |
| Marlies Schwegler Foundation, Germany |
| Max Planck Society, Germany |
| Mehdorn Family Foundation, Germany |
| Monika Kutzner Foundation, Germany |
| Raising for effective giving, Germany |
| Reinhard Frank Foundation, Germany |
| Robert Bosch Foundations, Germany |
| Rudolf Bartling Foundation, Germany |
| Stefan Morsch Foundation, Germany |
| Sybille-Hahne Foundation, Germany |
| Volkswagen Foundation Germany, Germany |
| Von Behring-Röntgen Foundation, Germany |
| Walter Schulz Foundation, Germany |
| Wilhelm Sander Foundation, Germany |
| Wilhelm Vaillant Foundation, Germany |
| African Cancer Organisation, Ghana |
| Breast Care International, Ghana |
| Cancer Society of Ghana, Ghana |
| Ahlen Foundation, Greece |
| Bodossaki Foundation, Greece |
| Cretan Association For Biomedical Research - CABR, Greece |
| Hellenic Cooperative Oncology Group, Greece |
| Hellenic Health Foundation, Greece |
| Hellenic Society of Medical Oncology - HeSMO, Greece |
| Hellenic Society of Oncology, Greece |
| Joseph and Esther Gani Foundation, Greece |
| Stavros Niarchos Foundation, Greece |
| Guatemalan League Against Cancer, Guatemala |
| Hungarian League Against Cancer, Hungary |
| National Institute of Oncology, Hungary |
| The Foundation for Cancer Research Szeged, Hungary |
| Icelandic Cancer Society, Iceland |
| Walking for Breast Cancer Research, Iceland |
| Biocon Foundation, India |
| Biotechnology Industry Research Assistance Council, India |
| Cancer Foundation of India, India |
| Cancer Patients Aid Association, India |
| Hyderabad Eye Research Foundation, India |
| Indian Cancer Society, India |
| Infosys Foundation, India |
| Rajiv Gandhi Cancer Institute and Research Centre, India |
| Saroj Gupta Cancer Centre and Research Institute, India |
| Villoo Poonawalla charitable foundation, India |
| Indonesian Cancer Foundation, Indonesia |
| Breakthrough Cancer Research, Ireland |
| Breast Cancer Research, Ireland |
| Cancer Clinical Research Trust, Ireland |
| Cancer Focus Northern Ireland, Ireland |
| Children's Medical Research Foundation, Ireland |
| Friends of the Cancer Center - Belfast City Hospital, Ireland |
| Irish Cancer Society, Ireland |
| Mid-Western Cancer Foundation, Ireland |
| Northeast Cancer Research and Education Trust, Ireland |
| Saint John of God Foundation, Ireland |
| Science Foundation Ireland, Ireland |
| The Children’s Leukaemia Research Project, Ireland |
| The Hope Foundation, Ireland |
| The Noyes Brain Tumor Foundation, Ireland |
| Adelis Foundation, Israel |
| Alhayat Association for Cancer Control, Israel |
| American Physicians Fellowship For Medicine In Israel, Israel |
| Environment and Health Fund of Israel, Israel |
| Israel Cancer Association, Israel |
| Israel Cancer Research Fund, Israel |
| Israel Science Foundation, Israel |
| Jacob and Gitla Zukier Medical Fund Oncology Fellowship Program, Israel |
| Patient's Friend's Society of Jerusalem, Israel |
| Alliance Against Cancer, Italy |
| Angela Serra Association For Cancer Research, Italy |
| Association against cancer of Umbria - AUCC, Italy |
| Associazione Iblea Per La Ricerca Epidemiologica - AIRE-ONLUS, Italy |
| Associazione Italiana Malati di Cancro Parenti e Amici - AIMAC, Italy |
| Associazione Romana Ricerca Dermatologica - ARRD, Italy |
| Associazione Volontari Italiani Del Sangue, Italy |
| Augusto Association for Life, Italy |
| Banca Del Monte Di Lombardia Foundation, Italy |
| Banco di Napoli Foundation, Italy |
| Barilla Center For Food and Nutrition Foundation, Italy |
| Bianca Garavaglia Association, Italy |
| Carife Foundation, Italy |
| Carige Foundation, Italy |
| Cariparo Foundation, Italy |
| Cariplo Foundation, Italy |
| Carisbo Bologna Foundation, Italy |
| Cassa di Risparmio di Calabria e di Lucania Foundation, Italy |
| Cassa di Risparmio di Modena Foundation, Italy |
| Cassa di Risparmio di Perugia Foundation, Italy |
| CRT Foundation, Italy |
| Edo Ed Elvo Tempia Valenta Foundation, Italy |
| Fondazione Cassa di Risparmio di Verona, Italy |
| Fondazione Città della Speranza, Italy |
| Fondazione del Monte di Bologna e Ravenna, Italy |
| Fondazione Italiana Linfomi Onlus, Italy |
| Fondazione Melanoma Onlus, Italy |
| Foundation Cassa Di Risparmio di Firenze, Italy |
| G.B.Morgagni Mediterranean Foundation, Italy |
| Guido Berlucchi Foundation, Italy |
| Il Fondo di Gio Onlus, Italy |
| Italian Association against Leukemia, Lymphoma and Myeloma - AIL, Italy |
| Italian Association for Brain Cancer - AITC, Italy |
| Italian Association for Cancer Research - AIRC, Italy |
| Italian League for the Fight Against Cancer - LILT, Italy |
| Italian Neuroblastoma Association, Italy |
| Molinette Research Foundation, Italy |
| Nadia Valsecchi Foundation, Italy |
| Oncologia Niguarda Onlus Foundation, Italy |
| Pediatric Oncology and Neuroblastoma Association - OPEN, Italy |
| Piemontese Foundation for Research on Cancer, Italy |
| Roma Foundation, Italy |
| San Paolo Foundation - Compagnia di San Paolo, Italy |
| Sardinian Foundation, Italy |
| Telethon Foundation, Italy |
| The Federico and Elvia Faggin Foundation, Italy |
| Umberto Veronesi Foundation, Italy |
| Ursula and Giorgio Cytron Foundation, Italy |
| Voluntary Association for the Promotion of Cancer Research - AVOPORIT, Italy |
| Akiyama Life Science Foundation, Japan |
| Cell Science Research Foundation, Japan |
| Children's Cancer Association of Japan, Japan |
| Daiichi Sankyo Foundation of Life Science, Japan |
| Daiwa Securities Health Foundation, Japan |
| Foundation for Biomedical Research and Innovation - FBRI, Japan |
| Foundation for Promotion of Cancer Research, Japan |
| Friends of Leukemia Research Fund, Japan |
| Fukuoka Foundation for Sound Health Cancer Research Fund, Japan |
| Fukuoka Industry, Science and Technology Foundation, Japan |
| Hyogo Science and Technology Association, Japan |
| Ichiro Kanehara Foundation, Japan |
| Inoue Enryo Memorial Foundation, Japan |
| Japan Brain Foundation, Japan |
| Japan Cancer Society, Japan |
| Japan China Medical Association, Japan |
| Japan Foundation for Applied Enzymology, Japan |
| Japan Health Sciences Foundation, Japan |
| Japan Hospice Palliative Care Foundation, Japan |
| Japan Leukemia Research Fund, Japan |
| Japan Lung Cancer Society, Japan |
| Japan Medical Association, Japan |
| Japan Research Foundation for Clinical Pharmacology, Japan |
| Japan Society of Clinical Oncology, Japan |
| Japan Society of Gynecologic Oncology, Japan |
| Japanese Breast Cancer Society, Japan |
| Japanese Foundation for Multidisciplinary Cancer Treatment, Japan |
| Japanese Society for Cancer of the Colon and Rectum, Japan |
| Japanese Society of Gastroenterology, Japan |
| Kanae Foundation for the Promotion of Medical Science, Japan |
| Kanagawa Health Foundation, Japan |
| Kanzawa Medical Research Foundation, Japan |
| Kato Memorial Bioscience Foundation, Japan |
| Katsuzo and Kiyo Aoshima Memorial Funds, Japan |
| Kidani Memorial Fund, Japan |
| Kobayashi Foundation for Cancer Research, Japan |
| Kodama Memorial Fund for Medical Research, Japan |
| Kowa Life Science Foundation, Japan |
| Kurozumi Medical Foundation, Japan |
| Mami Mizutani Foundation, Japan |
| Meiji Yasuda Foundation of Health and Welfare, Japan |
| Mishima Kaiun Memorial Foundation, Japan |
| Mitsui Sumitomo Insurance Welfare Foundation, Japan |
| Mizutani Foundation for Glycoscience, Japan |
| Mochida Memorial Foundation for Medical and Pharmaceutical Research, Japan |
| Nippon Foundation, Japan |
| OITA Cancer Research Foundation, Japan |
| Ono Medical Research Foundation, Japan |
| Osaka Medical Research Foundation for Incurable Diseases, Japan |
| Pancreas Research Foundation of Japan, Japan |
| Princess Takamatsu Cancer Research Fund, Japan |
| Public Health Research Foundation, Japan |
| Sagawa Cancer Research Promotion Public Interest Foundation, Japan |
| Sapporo Jikeikai Tomoiki Foundation, Japan |
| Sasaki Foundation, Japan |
| Senri Life Science Foundation, Japan |
| Senshin Medical Research Foundation, Japan |
| Suhara Memorial Foundation, Japan |
| Sumitomo Foundation, Japan |
| Suzuken Memorial Foundation, Japan |
| Takeda Science Foundation, Japan |
| Terumo Foundation for Life Sciences and Arts, Japan |
| The Futaba Electronics Memorial Foundation, Japan |
| The Hokkoku Cancer Foundation, Japan |
| The Japanese Foundation For Research and Promotion of Endoscopy, Japan |
| The Japanese Urological Association, Japan |
| The Naito Foundation, Japan |
| The Nakatomi Foundation, Japan |
| The Tokyo Biochemical Research Foundation, Japan |
| The Toyota Foundation, Japan |
| The Uehara Memorial Foundation, Japan |
| Vehicle Racing Commemorative Foundation, Japan |
| Wesco Scientific Promotion Foundation, Japan |
| Yamada Science Foundation, Japan |
| Yokohama Foundation for Advancement of Medical Science, Japan |
| Yokoyama Foundation for Clinical Pharmacology, Japan |
| King Hussein Cancer Foundation, Jordan |
| Clinical Research Foundation Eeu, Latvia |
| Lebanese Cancer Society, Lebanon |
| Adolf H Lundin Charitable Foundation, Liechtenstein |
| Action Lions Vaincre Le Cancer Association, Luxembourg |
| Association Een Häerz fir kriibskrank Kanner Asbl, Luxembourg |
| Fondation Cancer, Luxembourg |
| Fondation Recherche Cancer et Sang, Luxembourg |
| Kriibskrank Kanner Foundation, Luxembourg |
| Luxembourg National Research Fund, Luxembourg |
| Recherches Scientifiques Luxembourg, Luxembourg |
| Televie, Luxembourg |
| Akbaraly Foundation, Madagascar |
| Women Coalition Against Cancer in Malawi - WOCACA, Malawi |
| Cancer Research Malaysia, Malaysia |
| National Cancer Council of Malaysia - MAKNA, Malaysia |
| Cancer Society of Maldives, Maldives |
| Action For Breast Cancer Foundation, Malta |
| Carlos Slim Foundation, Mexico |
| Claudio X Gonzalez Family Foundation, Mexico |
| Mexican Society of Oncology, Mexico |
| Tómatelo a Pecho, A.C., Mexico |
| National Cancer Council of Mongolia, Mongolia |
| Lalla Salma Foundation - Cancer Prevention and Treatment, Morocco |
| Cancer Society Nepal, Nepal |
| Abe Bonnema Foundation, Netherlands |
| Biltema Foundation, Netherlands |
| Chanrone Foundation, Netherlands |
| Cornelis Visser Foundation, Netherlands |
| De Drie Lichten Foundation, Netherlands |
| Dutch Digestive Foundation, Netherlands |
| Dutch Heart Foundation, Netherlands |
| Dutch Pink Ribbon Foundation, Netherlands |
| Erasmus Trust Fund, Netherlands |
| European Association of Urology, Netherlands |
| European Waldenström Macroglobulinemia Network - EWMnetwork, Netherlands |
| Groningen Melanoma and Sarcoma Foundation, Netherlands |
| Health Foundation Limburg, Netherlands |
| Jan Kornelis de Cock Foundation, Netherlands |
| Landsteiner Foundation for Blood Transfusion Research, Netherlands |
| Lisa Waller Hayes - Living With Hope Foundation, Netherlands |
| Maurits and Anna de Kock Foundation, Netherlands |
| Niels Stensen Fellowship, Netherlands |
| Nijbakker-Morra Foundation, Netherlands |
| NutsOhra Foundation, Netherlands |
| Rene Vogels Foundation, Netherlands |
| Semmy Foundation, Netherlands |
| Stichting af Jochnick Foundation, Netherlands |
| Synovial Sarcoma Research Foundation, Netherlands |
| The Dutch Cancer Society - KWF, Netherlands |
| The KiKa Foundation, Netherlands |
| Think-Pink, Netherlands |
| Top Institute Pharma - TI Pharma, Netherlands |
| University Medical Center Utrecht - Friends of the UMC Utrecht Foundation, Netherlands |
| Van Stekelenburg Family, Netherlands |
| Vanderes Foundation, Netherlands |
| Villa Joep Foundation, Netherlands |
| Auckland Cancer Society, New Zealand |
| Auckland Medical Research Foundation, New Zealand |
| Breast Cancer Foundation of New Zealand, New Zealand |
| Cancer Research Trust NZ, New Zealand |
| Cancer Society of New Zealand, New Zealand |
| Four Winds Foundation Ltd., New Zealand |
| Marijanna Kumerich Family, New Zealand |
| Movimiento Contra el Cáncer Foundation, Nicaragua |
| Access to Basic (Medical) Care Foundation, Nigeria |
| Breast Cancer Association of Nigeria - BRECAN, Nigeria |
| Dangote Foundation, Nigeria |
| Ego Bekee Cancer Foundation, Nigeria |
| Foundation for Carcinoma of the Prostate Transatlantic, Nigeria |
| Lifetouch Africa, Nigeria |
| Nigerian Cancer Society, Nigeria |
| Sebeccly Cancer Care and Support Centre, Nigeria |
| Society of Oncology and Cancer Research of Nigeria, Nigeria |
| The Dorcas Cancer Foundation, Nigeria |
| Astri and Birger Torsteds Foundation, Norway |
| Bergen Research Foundation, Norway |
| Folke Hermansen Foundation, Norway |
| Inger and John Fredriksen Ovarian Cancer Research Foundation, Norway |
| Kristian Gerhard Jebsen Foundation, Norway |
| Nordic Cancer Union, Norway |
| Norwegian Cancer Society, Norway |
| Norwegian Foundation for Health and Rehabilitation, Norway |
| Norwegian Women's Public Health Association, Norway |
| Oddrun Mjåland Foundation for Cancer Research, Norway |
| The Norwegian Radium Hospital Foundation, Norway |
| Oman Cancer Association, Oman |
| Shaukat Khanum Memorial Cancer Hospital and Research Centre, Pakistan |
| Centro Paraguayo de Estudios de Población - CEPEP, Paraguay |
| Peruvian Cancer Foundation, Peru |
| Peruvian League Against Cancer, Peru |
| Foundation for Polish Science, Poland |
| Polish Anti-Cancer Committee, Poland |
| Polpharma Scientific Foundation, Poland |
| Calouste Gulbenkian Foundation, Portugal |
| Champalimaud Foundation, Portugal |
| Grünenthal Foundation, Portugal |
| Portuguese Association Oncology Nurse - AEOP, Portugal |
| Portuguese Cancer League, Portugal |
| Qatar Cancer Society, Qatar |
| Qatar Foundation, Qatar |
| Qatar National Research Fund, Qatar |
| Equal Right to Life, Russia |
| Prince Mohammed bin Salman bin Abdulaziz Foundation - MiSK, Saudi Arabia |
| Saudi Cancer Society, Saudi Arabia |
| Zahra Breast Cancer Association, Saudi Arabia |
| Leukemia and Lymphoma Foundation, Singapore |
| Singapore Cancer Society, Singapore |
| Singapore Millennium Foundation, Singapore |
| SingHealth Foundation, Singapore |
| The Skin Cancer Institute, Singapore |
| Slovak League Against Cancer, Slovakia |
| Cancer Association of South Africa, South Africa |
| Zoleka Mandela Foundation, South Africa |
| Korea Breast Cancer Society, South Korea |
| Korean Cancer Association, South Korea |
| SK Telecom Research Fund, South Korea |
| Alfonso Martín Escudero Foundation, Spain |
| Alicia Pueyo Fund, Spain |
| Banco Bilbao Vizcaya Argentaria Foundation, Spain |
| Basque Foundation for Health Research and Innovation - BIOEF, Spain |
| BBVA Foundation, Spain |
| Botin Foundation, Spain |
| Caja Navarra Foundation, Spain |
| CajaCanarias Foundation, Spain |
| Cajastur Foundation, Spain |
| Catalan Institution for Research and Advanced Studies - ICREA, Spain |
| Cellex Foundation, Spain |
| Childhood Cancer Association of Madrid - ASION, Spain |
| Foundation for Applied Medical Research - FIMA, Spain |
| Íñigo Álvarez de Toledo Renal Foundation, Spain |
| Inocente Inocente Foundation, Spain |
| José Carreras Leukaemia Foundation, Spain |
| José Luis Castaño Foundation, Spain |
| La Caixa Banking Foundation, Spain |
| La Marató de TV3 Foundation, Spain |
| La Sonrisa de Alex Foundation, Spain |
| Marques de Valdecilla Foundation, Spain |
| Mutua Madrileña Foundation, Spain |
| Olga Torres Foundation, Spain |
| Pablo Ugarte Association, Spain |
| Pro CNIC Foundation, Spain |
| Ramón Areces Foundation, Spain |
| Spanish Association Against Cancer - AECC, Spain |
| Spanish Association for Breast Cancer - FECMA, Spain |
| Spanish Ovarian Cancer Research Group - GEICO, Spain |
| Spanish Society of Haematology - SEHH, Spain |
| Spanish Society of Medical Oncology - SEOM, Spain |
| Valencia Oncology Institute Foundation, Spain |
| Vall Hebron Institute of Oncology, Spain |
| Ake Wiberg Foundation, Sweden |
| Albert Pahlsson Research Foundation, Sweden |
| Alex and Eva Wallström Foundation, Sweden |
| Alfred Österlunds Foundation, Sweden |
| Anna And Edwin Bergers Foundation, Sweden |
| Anna-Lisa And Bror Björnsson Foundation, Sweden |
| Assar Gabrielssons Fond, Sweden |
| Axel Linders Foundation, Sweden |
| Bengt Ihre Research Fellowship, Sweden |
| Berta Kamprad Foundation, Sweden |
| Berth Von Kantzows Foundation, Sweden |
| Cancer And Allergy Foundation, Sweden |
| Cancer Research Foundation of Northern Sweden, Sweden |
| Cancer Research Funds of Radiumhemmet, Sweden |
| Cancer Society in Stockholm, Sweden |
| Clas Groschinskys Memorial Fund, Sweden |
| Einar and Inga Nilsson Foundation, Sweden |
| Einar Willumsen Foundation, Sweden |
| Erik, Karin, and Gösta Selanders Foundation, Sweden |
| Erna and Victor Hasselblad Foundation, Sweden |
| Ernhold Lundströms Foundation, Sweden |
| Göran Gustafsson Foundation, Sweden |
| Gothenburg Medical Society, Sweden |
| Gunnar Nilsson Cancer Foundation, Sweden |
| Gyllenstierna Krapperup´s Foundation, Sweden |
| Harald and Greta Jeansson Foundation, Sweden |
| Helleday Foundation, Sweden |
| Hjalmar Svensson Foundation, Sweden |
| Hulda and Conrad Mossfelt Foundation, Sweden |
| IngaBritt and Arne Lundberg's Research Foundation, Sweden |
| Jane and Dan Olssons Foundation, Sweden |
| Kaplan Cancer Research Fund, Sweden |
| Karolinska Institute Foundation, Sweden |
| King Gustaf V and Queen Victoria's Foundation of Freemasons, Sweden |
| King Gustaf V Jubilee Clinic Research Foundation, Sweden |
| Knut and Alice Wallenberg Foundation, Sweden |
| Lars Hierta Memorial Foundation, Sweden |
| Magnus Bergvalls Foundation, Sweden |
| Marianne and Marcus Wallenberg Foundation, Sweden |
| Märit and Hans Rausing Fund, Sweden |
| Märta and Gunnar V. Philipsons Foundation, Sweden |
| Martin Rinds Foundation, Sweden |
| Mary Béves Foundation for Childhood Cancer Research , Sweden |
| Nilsson-Ehle Endowment, Sweden |
| Olle Engkvist Byggmästare Foundation, Sweden |
| Ollie and Elof Ericsson Foundation, Sweden |
| Per-Eric and Ulla Schyberg Foundation, Sweden |
| Ragnar Söderberg Foundation, Sweden |
| Royal Physiographic Society of Lund, Sweden |
| Royal Swedish Academy of Sciences, Sweden |
| Sahlgrenska University Hospitals Research Foundations, Sweden |
| Sigurd and Elsa Golje Memorial Fund, Sweden |
| Skåne University Hospital Foundation, Sweden |
| Sten A. Olsson Foundation for Research and Culture, Sweden |
| Sweden–America Foundation, Sweden |
| Swedish Association of Local Authorities and Regions - SKL, Sweden |
| Swedish Brain Foundation, Sweden |
| Swedish Breast Cancer Association - BRO, Sweden |
| Swedish Cancer Society, Sweden |
| Swedish Childhood Cancer Foundation - Barncancer Foundation, Sweden |
| Swedish Cultural Foundation in Finland, Sweden |
| Swedish Diabetes Association, Sweden |
| Swedish Foundation for Strategic Research - SSF, Sweden |
| Swedish Pain Relief Foundation, Sweden |
| Swedish Rheumatism Association, Sweden |
| Swedish Society for Medical Research, Sweden |
| Swedish Society of Medicine, Sweden |
| The Crafoord Foundation, Sweden |
| The Erling-Persson Family Foundation, Sweden |
| The Foundation for Clinical Cancer Research in Jönköping, Sweden |
| The Kamprad Family Foundation, Sweden |
| The Swedish Heart-Lung Foundation, Sweden |
| The Swedish Knowledge Foundation, Sweden |
| The Swedish Laryng Foundation, Sweden |
| Tore Nilsons Foundation, Sweden |
| Torsten Söderberg Foundation, Sweden |
| Welander Finsen Foundation, Sweden |
| Wilhelm and Martina Lundgren Foundation, Sweden |
| Charles Rodolphe Brupbacher Foundation, Switzerland |
| Childhood Cancer Switzerland, Switzerland |
| Domarena Foundation, Switzerland |
| Ernst Göhner Foundation, Switzerland |
| European Society for Medical Oncology - ESMO, Switzerland |
| Fondation Philanthropia, Switzerland |
| Fondation Pierre Mercier pour la science, Switzerland |
| Fondation pour la Lutte contre le Cancer, Switzerland |
| Fondation pour la lutte contre le cancer et pour des recherches médico-biologiques, Switzerland |
| Foundation for Childhood Cancer Research Switzerland, Switzerland |
| Foundation for the Institute of Oncology Research, Switzerland |
| Foundation Nelia and Amadeo Barletta, Switzerland |
| GAVI Vaccine Alliance, Switzerland |
| Global Fund to Fight AIDS, Tuberculosis and Malaria, Switzerland |
| Gottfried and Julia Bangerter-Rhyner Foundation, Switzerland |
| Helmut Horten Foundation, Switzerland |
| Institut National pour l'Epidémiologie et l'Enregistrement du Cancer - NICER, Switzerland |
| Institut universitaire romand de Santé au Travail, Switzerland |
| International Breast Cancer Study Group, Switzerland |
| International Society for Geriatric Oncology - SIOG, Switzerland |
| ISREC Foundation, Switzerland |
| Kurt and Senta Herrmann Foundation, Switzerland |
| MEDIC Foundation, Switzerland |
| Nestlé Foundation, Switzerland |
| Novartis Foundation, Switzerland |
| Nuovo-Soldati Foundation, Switzerland |
| Rising Tide Foundation for Clinical Cancer Research, Switzerland |
| Sassella Foundation, Switzerland |
| Society in Science - The Branco Weiss Fellowship, Switzerland |
| Swiss Bridge, Switzerland |
| Swiss Cancer League, Switzerland |
| Swiss Cancer League - Swiss Cancer Research, Switzerland |
| Swiss Cancer Research Foundation, Switzerland |
| Swiss Children's Cancer Aid, Switzerland |
| Swiss Group for Clinical Cancer Research - SAKK, Switzerland |
| Swiss National Science Foundation - SNF, Switzerland |
| Swiss Paediatric Oncology Group - SPOG, Switzerland |
| Swiss Research Foundation for Electricity and Mobile Communication, Switzerland |
| Union for International Cancer Control - UICC, Switzerland |
| Union Suisse contre le Cancer - Oncosuisse, Switzerland |
| Walter L. und Johanna Wolf Foundation, Switzerland |
| Werner and Hedy Berger-Janser Foundation for the Study of Cancer, Switzerland |
| Werner Siemens Foundation, Switzerland |
| Chong Hin Loon Memorial Cancer, Taiwan |
| Li-Yang Sheen Medical Education Memorial Foundation, Taiwan |
| Szu-Yuan Research Foundation of Internal Medicine, Taiwan |
| Taiwan Brain Disease Foundation, Taiwan |
| Taiwan Clinical Oncology Research Foundation, Taiwan |
| Taiwan Liver Research Foundation, Taiwan |
| Teh-Tzer Study Group for Human Medical Research Foundation, Taiwan |
| Tseng-Lien Lin Foundation, Taiwan |
| African Palliative Care Association, Tanzania |
| Health Lifestyle Foundation, Tanzania |
| The Thai Society of Hematology, Thailand |
| Tobias Foundation, Trinidad and Tobago |
| Turkish Association for Cancer Research and Control , Turkey |
| Turkish Society for Radiation Oncology, Turkey |
| Turkish Society of Medical Oncology, Turkey |
| Urooncology Society in Turkey, Turkey |
| Estella Foundation Uganda, Uganda |
| Khalifa Bin Zayed Al Nahyan Foundation, United Arab Emirates |
| Abbie's Army, United Kingdom |
| Action Against Cancer, United Kingdom |
| Action Medical Research, United Kingdom |
| Addenbrookes Charitable Trust, United Kingdom |
| Alzheimer's Research UK, United Kingdom |
| Arthritis Research Uk, United Kingdom |
| Asthma UK, United Kingdom |
| Barts Charity, United Kingdom |
| Beatson Cancer Charity, United Kingdom |
| Beckett Foundation, United Kingdom |
| Bloodwise, United Kingdom |
| Blue Skye Thinking, United Kingdom |
| Bob Champion Cancer Trust, United Kingdom |
| Bobby Moore Fund, United Kingdom |
| Bone Cancer Research Trust, United Kingdom |
| Bournemouth Leukaemia Fund, United Kingdom |
| Bowel Disease Research Foundation, United Kingdom |
| Brain Tumour Charity, United Kingdom |
| Brain Tumour Research, United Kingdom |
| Breast Cancer Hope, United Kingdom |
| Breast Cancer Now, United Kingdom |
| British Heart Foundation, United Kingdom |
| British Lung Foundation, United Kingdom |
| British Skin Foundation, United Kingdom |
| Cancer And Polio Research Fund, United Kingdom |
| Cancer Research Uk - CRUK, United Kingdom |
| Cancer Research Wales, United Kingdom |
| Cancer52, United Kingdom |
| Candlelighters Trust, United Kingdom |
| Children With Cancer UK, United Kingdom |
| Children's Investment Fund Foundation, United Kingdom |
| Chris Lucas Trust, United Kingdom |
| Chris Rokos Fellowship in Evolution and Cancer, United Kingdom |
| CLIC Sargent, United Kingdom |
| Crawdaddy Foundation, United Kingdom |
| Cris Cancer Foundation, United Kingdom |
| Crohn’S And Colitis Uk, United Kingdom |
| D. J. Fielding Medical Research Trust, United Kingdom |
| Dimbleby Cancer Care, United Kingdom |
| Federation of European Biochemical Societies, United Kingdom |
| Fight for Sight, United Kingdom |
| Friends of ANCHOR, United Kingdom |
| Garfield Weston Fondation, United Kingdom |
| Genesis Research Trust, United Kingdom |
| Global Alliance for Chronic Diseases, United Kingdom |
| Great Ormond Street Hospital Children's Charity, United Kingdom |
| GSM Association, United Kingdom |
| Guy's and St Thomas' Charity, United Kingdom |
| Healthcare Quality Improvement Partnership Ltd., United Kingdom |
| Hodge Foundation, United Kingdom |
| International Society of Antimicrobial Chemotherapy, United Kingdom |
| John and Lucille van Geest Foundation, United Kingdom |
| Kidney Cancer Scotland, United Kingdom |
| Kidney Research UK, United Kingdom |
| Lady Tata Memorial Trust, United Kingdom |
| Leeds Teaching Hospitals Charitable Foundation, United Kingdom |
| Lymphoma Research Trust, United Kingdom |
| Macmillan Cancer Support, United Kingdom |
| Masonic Charitable Foundation, United Kingdom |
| Medical Research Scotland, United Kingdom |
| Myeloma UK, United Kingdom |
| NC3RS, United Kingdom |
| Neuroblastoma UK, United Kingdom |
| Newcastle Hospitals NHS Charity, United Kingdom |
| North of England Children's Cancer Research Fund, United Kingdom |
| North West Cancer Research, United Kingdom |
| Nottingham Hospitals Charity, United Kingdom |
| Oracle Cancer Trust, United Kingdom |
| Orchid Cancer Appeal, United Kingdom |
| Ovarian Cancer Action, United Kingdom |
| Pancreatic Cancer Research Fund, United Kingdom |
| Pancreatic Cancer UK, United Kingdom |
| Pelican Cancer Foundation, United Kingdom |
| Peter Stebbings Memorial Charity, United Kingdom |
| Prevent Breast Cancer, United Kingdom |
| Prostate Action, United Kingdom |
| Prostate Cancer Research Centre, United Kingdom |
| Prostate Cancer UK, United Kingdom |
| Raymond And Beverly Sackler Foundation, United Kingdom |
| Renal Cancer Research Fund, United Kingdom |
| Ronald and Rita McAulay Foundation, United Kingdom |
| Rosetrees Trust, United Kingdom |
| Roy Castle Lung Cancer Foundation, United Kingdom |
| Royal College of Radiologists, United Kingdom |
| Royal College of Surgeons of Edinburgh, United Kingdom |
| Royal College of Surgeons of England, United Kingdom |
| Royal Society of England, United Kingdom |
| Samantha Dickson Brain Tumour Trust, United Kingdom |
| Sarcoma UK, United Kingdom |
| Sean Crummey Foundation, United Kingdom |
| Sheffield Hospitals Charity, United Kingdom |
| St Peter's Trust, United Kingdom |
| St Vincent's Clinic Foundation, United Kingdom |
| Stroke Association, United Kingdom |
| Target Ovarian Cancer Charity, United Kingdom |
| Teenage Cancer Trust, United Kingdom |
| Tenovus Cancer Care, United Kingdom |
| The Brain Tumour Charity, United Kingdom |
| The Christie NHS Foundation Trust, United Kingdom |
| The City Bridge Trust, United Kingdom |
| The European Association for Cancer Research, United Kingdom |
| The Eve Appeal, United Kingdom |
| The Health Foundation, United Kingdom |
| The Howat Foundation, United Kingdom |
| The Jane Gibson Charitable Trust Fund, United Kingdom |
| The JGW Patterson Foundation, United Kingdom |
| The Kay Kendall Leukaemia Fund, United Kingdom |
| The Kirby Laing Foundation, United Kingdom |
| The Leverhulme Trust, United Kingdom |
| The Lyla Nsouli Foundation, United Kingdom |
| The Pathological Society of Great Britain and Ireland, United Kingdom |
| The Royal Society, United Kingdom |
| The Urology Foundation, United Kingdom |
| The Wolfson Foundation, United Kingdom |
| Tuberculosis Sclerosis Association, United Kingdom |
| Wellbeing of Women, United Kingdom |
| Wellcome Trust, United Kingdom |
| Wessex Cancer Trust, United Kingdom |
| Wessex Medical Research, United Kingdom |
| Weston Park Hospital Cancer Charity, United Kingdom |
| World Cancer Research Fund International - WCRF, United Kingdom |
| World Child Cancer UK, United Kingdom |
| Worldwide Cancer Research, United Kingdom |
| Yorkshire Cancer Research, United Kingdom |
| A Kids' Brain Tumor Cure Foundation - Paediatric Low-Grade Astrocytomas Foundation, USA |
| A Race Against Breast Cancer, USA |
| A Sister's Hope, USA |
| Aaron and Martha Schecter Private Foundation, USA |
| Aarons Neuroblastoma Cancer Foundation, USA |
| Abraham & Gizella Berger Foundation for Cancer Research, USA |
| Accelerate Brain Cancer Cure Foundation, USA |
| Adele McKinnon Research Fund for Breast Cancer-Related Lymphedema, USA |
| Adenoid Cystic Carcinoma Research Foundation, USA |
| Adler Foundation Inc, USA |
| Adrienne Helis Malvin Medical Research Foundation, USA |
| Advanced Medical Research Foundation, USA |
| Advances in Neuroblastoma Research Association, USA |
| Advancing A Healthier Wisconsin Endowment, USA |
| African Organisation for Research and Training in Cancer - AORTIC, USA |
| Agnes Brown Duggan Endowment, USA |
| Aid for Cancer Research, USA |
| Aim At Melanoma Foundation, USA |
| Aim With Immunotherapy, USA |
| AKTIV Against Cancer, USA |
| Al Copeland Foundation, USA |
| Alan B Brown Chair In Molecular Genomics, USA |
| Alan B. Slifka Foundation, USA |
| Albert And Mary Lasker Foundation, USA |
| Albertsons Companies Foundation, USA |
| Alexander And Margaret Stewart Trust, USA |
| Alexander M. and June L. Maisin Foundation, USA |
| Alex's Army Foundation, USA |
| Alex's Lemonade Stand Foundation, USA |
| Alfred P. Sloan Foundation, USA |
| Alfred Taubman Medical Research Institute, USA |
| Aline W. and L.S. Skaggs Foundation, USA |
| Aline W. and L.S. Skaggs Foundation - Skaggs Institute for Chemical Biology, USA |
| Allen Foundation, USA |
| Alliance For Cancer Gene Therapy Foundation, USA |
| Alliance For Clinical Trials In Oncology, USA |
| Alliance of Cardiovascular Researchers, USA |
| Allison's Hope Foundation, USA |
| Alma Toorock Memorial for Cancer Research Inc., USA |
| Alternative Cancer Research Fund, USA |
| Aly Wolff Foundation, USA |
| Alzheimer's Association, USA |
| Amanda Styles Cirelli Foundation for Cancer Research Inc., USA |
| Amc Cancer Research Center, USA |
| American Association For Cancer Research, USA |
| American Association for Dental Research - IADR, USA |
| American Association of Blood Banks - AABB, USA |
| American Association of Immunologists, USA |
| American Asthma Foundation, USA |
| American Brain Tumor Association, USA |
| American Cancer Fund, USA |
| American Cancer Research Center and Foundation - ACRCF, USA |
| American Cancer Society, Inc., USA |
| American College of Gastroenterology, USA |
| American College of Surgeons, USA |
| American Diabetes Association, USA |
| American Federation For Aging Research, USA |
| American Friends of Rambam Medical Center, USA |
| American Gastroenterological Association, USA |
| American Heart Association, USA |
| American Institute For Cancer Research - AICR, USA |
| American Lebanese Syrian Associated Charities - ALSAC, USA |
| American Lung Association, USA |
| American Melanoma Foundation, USA |
| American Psychosocial Oncology Society Inc., USA |
| American Skin Association, USA |
| American Society For Radiation Oncology, USA |
| American Society of Breast Disease, USA |
| American Society of Clinical Oncology - ASCO, USA |
| American Society of Colon And Rectal Surgeons Foundation, USA |
| American Society of Hematology, USA |
| American Society of Pediatric Hematology/Oncology, USA |
| American Society of Preventive Oncology Inc., USA |
| American Thyroid Association - ATA, USA |
| American-Italian Cancer Foundation, USA |
| Andrew McDonough B+ Foundation, USA |
| Andrew Sabin Family Foundation, USA |
| Andy Caress Melanoma Foundation, USA |
| Andy Derr Foundation for Kidney Cancer Research, USA |
| Angiosarcoma Awareness Inc., USA |
| Ann Rife Cox Chair In Gynecology, USA |
| Anna Needs Neuroblastoma Answers Inc., USA |
| Anne Rita Monahan Foundation, USA |
| Anne T. and Robert M. Bass Endowed Faculty Scholarship in Pediatric Cancer and Blood Diseases, USA |
| Annenberg Foundation, USA |
| Anthony Bullock III Foundation, USA |
| Anthony C Hunter Melanoma Foundation, USA |
| Anthony Rizzo Family Foundation, USA |
| Anton B. Burg Foundation, USA |
| Aplastic Anemia And MDS International Foundation, USA |
| Arnold And Mabel Beckman Foundation, USA |
| Arnold S Leonard Cancer Research Fund, USA |
| Art of The Brain, USA |
| Arthur B & Marion V Myers Scta Cancer Research Fund, USA |
| Arthur R Cores Esophageal Cancer Research Foundation, USA |
| Artz Cure Sarcoma Foundation Inc., USA |
| Associates For Breast And Prostate Cancer Studies, USA |
| Association for Research of Childhood Cancer, USA |
| Association of Chinese Americans in Cancer Research, USA |
| Association of Oncology Social Work Inc., USA |
| Astellas USA Foundation, USA |
| Astrazeneca Healthcare Foundation, USA |
| Atanas Ilitch Osteosarcoma Foundation, USA |
| Atlanta Cancer Research and Education Foundation, USA |
| Audie and Kathy White Cancer Research Foundation Inc., USA |
| Augustine L Munoz Cancer Research Fund, USA |
| Avon Breast Cancer Crusade, USA |
| Avon Foundation For Women, USA |
| B*CURED, USA |
| Bald Beauties Project, USA |
| Bank of America Charitable Foundation, USA |
| Barbara Isackson Lung Cancer Research Fund, USA |
| Barbara K. Lipman Lymphoma Research Fund, USA |
| Basic Cancer Research Foundation, USA |
| Bcc Open for Cancer Research, USA |
| Be a Piece of the Solution, USA |
| Be the Difference Foundation, USA |
| Be The Match Foundation, USA |
| Bear Necessities Foundation, USA |
| Beatsarcoma Inc., USA |
| Beckman Coulter Foundation, USA |
| Ben Towne Foundation, USA |
| Bennett Family Foundation, USA |
| Betts Family Fund, USA |
| Betty Anne Asche Murray Distinguished Professorship, USA |
| Betty Hise Trust for Cancer Research, USA |
| Betty Minsk Foundation for Melanoma Research, USA |
| Big Ten Cancer Research Consortium Foundation Inc., USA |
| Bill And Melinda Gates Foundation, USA |
| Bill Rose Memorial Fundraiser for Melanoma Inc., USA |
| Bill Walter III Melanoma Research Fund Inc., USA |
| Blackout Melanoma, USA |
| Bladder Cancer Research Foundation, USA |
| Blast Glioblastoma, USA |
| Blavatnik Family Foundation, USA |
| Bloomberg Philantropies, USA |
| Blue Cross And Blue Shield Association, USA |
| Borstein Family Foundation, USA |
| Bosarge Family Foundation, USA |
| Botwinick Wolfensohn Foundation, USA |
| Bradley Omartin Melanoma Foundation, USA |
| Brady Cancer Research Institute the Hahnemann Medical College Hospital, USA |
| Brain and Behavior Research Foundation, USA |
| Brain Science Foundation, USA |
| Brain Tumor Funders Collaborative, USA |
| Breast Cancer Alliance, USA |
| Breast Cancer Care and Research Fund, USA |
| Breast Cancer Research and Assistance Fund, USA |
| Breast Cancer Research Foundation, USA |
| Brees Foundation for Breast Cancer Research, USA |
| Brenda B Macdonald Melanoma Foundation, USA |
| Bret Laczynski Brain Cancer Research Fund, USA |
| Brett Armin Sarcoma Foundation, USA |
| Brian Hays Melanoma Cancer Foundation Inc., USA |
| Brian J Levitas Lymphoma Foundation, USA |
| Brian MacIsaac Sarcoma Foundation, USA |
| Brian Piccolo Cancer Research Fund Inc., USA |
| BrightFocus Foundation, USA |
| Bristol-Myers Squibb Foundation, USA |
| Brittany Pasqual Pediatric Oncology Research Foundation, USA |
| Broach Foundation for Brain Cancer Research, USA |
| B-Strong Foundation, USA |
| Buckeyes Fore Cancer Research, USA |
| Buffone Family Gastrointestinal Cancer Research Fund, USA |
| Burroughs Wellcome Fund, USA |
| Butterfly Fund of East Tennessee Foundation, USA |
| California Health Care Foundation, USA |
| California Oncology Research Institute Inc., USA |
| California Walnut Commission, USA |
| Canary Foundation, USA |
| Cancer Aid and Research Foundation, USA |
| Cancer Free Kids Pediatric Cancer Research Alliance, USA |
| Cancer Immunotherapy Research Institute, USA |
| Cancer League of Colorado, USA |
| Cancer Research & Education Fund Inc., USA |
| Cancer Research & Treatment Fund Inc., USA |
| Cancer Research and Assistance Foundation Inc., USA |
| Cancer Research and Awareness, USA |
| Cancer Research and Biostatistics, USA |
| Cancer Research and Life Foundation, USA |
| Cancer Research Center, USA |
| Cancer Research Foundation, USA |
| Cancer Research Foundation of North Texas, USA |
| Cancer Research Institute - CRI, USA |
| Cancer Research Institute of West Tennessee, USA |
| Cancer Research Wellness Institute, USA |
| Cancer Vaccine and Cancer Immunotherapy Foundation Inc. - CVCI, USA |
| Caporella Family, USA |
| Carol M. Baldwin Breast Cancer Research Fund, Inc., USA |
| Carson Sarcoma Foundation, USA |
| Casey Z Porter Foundation for Brain Cancer Research, USA |
| Catholic Medical Center Research Foundation, USA |
| Causes For A Cure, USA |
| Celma Mastry Ovarian Cancer Foundation, USA |
| Centers For Disease Control And Prevention Foundation, USA |
| Chapel Hill Breast Cancer Research Foundation, USA |
| Charif Souki Cancer Research Fund, USA |
| Charles and Ann Johnson Foundation, USA |
| Charles and Patricia Heidelberger Foundation for Cancer Research, USA |
| Charles Rogers Gene Therapy Fund, USA |
| Charlotte Geyer Foundation, USA |
| Chemotherapy Foundation, USA |
| Cheryl Lockton Williams Endowment, USA |
| Chicago Bears - Bears Care, USA |
| Chicago Blood and Cancer Foundation, USA |
| Childhood Brain Tumor Foundation, USA |
| Children S Cancer Foundation, USA |
| Children S Tumor Foundation, USA |
| Children's Brain Tumor Foundation, USA |
| Children's Cancer and Blood Foundation, USA |
| Children's Cancer Research Fund, USA |
| Children's Leukemia Research Association, USA |
| Children's Neuroblastoma Cancer Foundation, USA |
| Children's Oncology Group Foundation, USA |
| Chris Robinson Memorial Fund for Cancer Research, USA |
| Cindy Rosencrans Fund for Triple Negative Breast Cancer, USA |
| Circle of Hope for Cancer Research Inc., USA |
| Claudia von Schilling Foundation for Breast Cancer Research, USA |
| Clayton Foundation For Research, USA |
| Clinton Family Foundation, USA |
| Clinton Foundation, USA |
| Clinton Health Access Initiative, USA |
| CLL Global Research Foundation, USA |
| Coca Cola Foundation, USA |
| Cody's Crew Foundation, USA |
| Colette Coyne Memorial Melanoma Foundation Inc., USA |
| Collaborative Ependymoma Research Network (CERN) Foundation, USA |
| Colleen's Dream Foundation, USA |
| College of American Pathologists Foundation, USA |
| Colorado Melanoma Foundation Inc., USA |
| Commonwealth Foundation For Cancer Research, USA |
| Commonwealth Foundation for Cancer Research Foundation, USA |
| Community Cancer Research Foundation Inc., USA |
| Compassionate Oncology Research Foundation, USA |
| Concern Foundation, USA |
| Congressional Black Caucus Foundation, USA |
| Connecticut Cancer Foundation, USA |
| Connective Tissue Oncology Society, USA |
| Connie And Craig Kimberley, USA |
| Connor's Cure, USA |
| Conquer Cancer Foundation, USA |
| Conrad Hilton Foundation, USA |
| Cookies for Kids' Cancer - Childhood Cancer Foundation, USA |
| Cowboys for Cancer Research Inc., USA |
| Craig H. Neilsen Foundation, USA |
| CRDF Global, USA |
| Creators Canvas Cancer Research Foundation, USA |
| Cridlan Fund, USA |
| Crohn's and Colitis Foundation of America, USA |
| Cure Cancer Foundation, USA |
| Cure Childhood Cancer, USA |
| CureSearch for Children's Cancer, USA |
| Cutaneous Lymphoma Foundation, USA |
| Cycle For Survival, USA |
| Daisy Foundation, USA |
| Dallas Family, USA |
| Damon Runyon Cancer Research Foundation, USA |
| Dana Foundation, USA |
| David And Cynthia Chapin, USA |
| David And Lucile Packard Foundation, USA |
| David and Patricia Giuliani Family Foundation, USA |
| David H. Koch Charitable Foundation, USA |
| David M. Rubenstein Foundation, USA |
| De Rosa Foundation for Colon Cancer Research and Prevention, USA |
| DeBartolo Family Foundation, USA |
| Deborah Bunn Alley Ovarian Cancer Research Foundation, USA |
| Deborah J McKenna Foundation for Cancer Research and Treatment Tr, USA |
| Degregorio Family Foundation, USA |
| Derek Jahn Beat Sarcoma Foundation, USA |
| Dermatology Foundation, USA |
| DeSanti Family Foundation, USA |
| Destroy Pancreatic Cancer Research and Care Foundation Inc., USA |
| Dhont Family Foundation, USA |
| Diana Helis Henry Medical Research Foundation, Inc., USA |
| Diney Goldsmith Breast Cancer Research and Education Foundation, USA |
| DIPG Collaborative, USA |
| Direct Relief, USA |
| Djerassi Cancer Research Foundation, USA |
| Donald P. Goldstein, MD, Trophoblastic Tumor Registry Endowment, USA |
| Donna and Jesse Garber Award for Cancer Research, USA |
| Doreen J Putrah Cancer Research Foundation, USA |
| Doris Duke Charitable Foundation, USA |
| DoubleStrand Foundation, USA |
| Dougherty Foundation, USA |
| Dr Amos Norman Pancreatic Cancer Research Foundation Inc., USA |
| Dr Josef Steiner Cancer Foundation, USA |
| Dr Kenneth B. McCredie Chair in Clinical Leukemia Research Endowment, USA |
| Dr Marnie Rose Foundation, USA |
| Dr Miriam and Sheldon G. Adelson Medical Research Foundation, USA |
| Dr Ralph And Marian Falk Medical Research Trust, USA |
| Dr Rath Research Institute, USA |
| Dr Robert Vigen Memorial Fund, USA |
| Drs Ji and Li Family Cancer Research Foundation, USA |
| Drs Martin and Dorothy Spatz Charitable Foundation, USA |
| Dunkin Donuts Rising Star Award, USA |
| Dwoskin Family Foundation, USA |
| Dyett Family Trophoblastic Disease Research And Registry Endowment, USA |
| E. Eric Muirhead Chair of Excellence in Pathology, USA |
| Early Overstreet Cancer Research Foundation, USA |
| Edward P. Evans Foundation, USA |
| Eileen D. Ludwig Endowed Fund for Thoracic Oncology Research, USA |
| Eileen Stein Jacoby Fund, USA |
| El Paso Foundation for Melanoma Research, USA |
| Elaine H. Snyder Cancer Research Trust, USA |
| Eleanor Roosevelt Institute for Cancer Research A/K/A Florence Sab, USA |
| Eli Lilly and Company Foundation, USA |
| Elise Anderson Neuroblastoma Research Fund, USA |
| Ellie Kavalieros DIPG Research Fund, USA |
| Elsa U. Pardee Foundation, USA |
| Emerald Foundation, USA |
| Emily Beazley's Kures for Kids, USA |
| Emma Jordon Kidz Fighting Cancer Foundation, USA |
| Ensign Cancer Research Foundation, USA |
| Ensign Endowment for Gynecologic Cancer Research, USA |
| Entertainment Industry Foundation, USA |
| Ernest Cockrell Jr. Distinguished Endowed Chair, USA |
| Estate of C. G. Johnson, USA |
| Estate of Norman Mancini, USA |
| Estate of Robert Griffiths, USA |
| Eugene and Connie Corasanti Lymphoma Research Fund, USA |
| Ewings Sarcoma Research Foundation, USA |
| Expect Miracles Foundation, USA |
| Eyad Karkoutly Lymphoma-Leukemia Research Foundation, USA |
| Eye Tumor Research Foundation, USA |
| F. M. Kirby Foundation, USA |
| Family Health International 360, USA |
| Fanconi Anemia Research Fund, USA |
| Fannie and John Hertz Foundation, USA |
| Farmer Family Foundation, USA |
| Farrah Fawcett Foundation, USA |
| Fashion Footwear Association of New York, USA |
| Ferrin Randall Zeitlin Foundation for Sarcoma Research, USA |
| Fidelity Foundation, USA |
| Fifth District Ahepa Cancer Research Foundation Inc., USA |
| Fighting Infectious Diseases in Emerging Countries - FIDEC, USA |
| Find the Cause Breast Cancer Foundation, USA |
| Flight Attendant Medical Research Institute - FAMRI, USA |
| Florida Breast Cancer Foundation, USA |
| Florida Society of Clinical Oncology Inc., USA |
| Ford Foundation, USA |
| Foreman Foundation, USA |
| Foundation Fighting Blindness, USA |
| Foundation for Applied Research in Gastrointestinal Oncology Fargo, USA |
| Foundation for Cancer Research and Education - FCRE, USA |
| Foundation for Research in Cell Biology Cancer and Cardiology, USA |
| Foundation for the Carolinas, USA |
| Foundation for Women's Cancer, USA |
| Foundation of UMDNJ, USA |
| Foundation to Cure Melanoma and Other Diseases, USA |
| Frank McGraw Memorial Chair in Cancer Research, USA |
| Fraternal Order of Eagles, USA |
| Fred C. and Katherine B. Andersen Foundation, USA |
| Fred L. Hartley Family Foundation, USA |
| Free To Breathe, USA |
| Friends 4 Cancer Research Inc., USA |
| Friends at Dana Farber Cancer Institute, USA |
| Friends for an Earlier Breast Cancer Test, USA |
| Frontier Science Foundation, USA |
| Fullerton College Foundation, USA |
| Fund A Cure For Pancreatic Cancer, USA |
| Fund For Ophthalmic Knowledge, Inc., USA |
| G. Harold and Y. Leila Mathers Charitable Foundation, USA |
| Gabrielles Angel Foundation for Cancer Research Inc., USA |
| Garcia-Corsini Family Fund, USA |
| Gary Kirby Charitable Fund for Cancer Research, USA |
| Gateway for Cancer Research, USA |
| Gattegno and Wechsler funds, USA |
| General Electric Foundation, USA |
| Generations Cancer Foundation, USA |
| Geoffrey Beene Foundation, USA |
| George M. Eisenberg Foundation for Charities, USA |
| George W. Bush Presidential Center, USA |
| George Whipple Professorship Endowment, USA |
| Georgia Cancer Coalition, USA |
| Georgia Center for Oncology Research and Education Inc., USA |
| Georgia Research Alliance, USA |
| Geriatric Oncology Consortium Inc., USA |
| Gerry Foundation, USA |
| Gerstner Family Foundation, USA |
| Gilder Foundation, USA |
| Giles W. and Elise G. Mead Foundation, USA |
| Gillson Longenbaugh Foundation, USA |
| GIST Cancer Research Fund, USA |
| Giulio D'Angio Endowed Chair in Neuroblastoma Research, USA |
| Give 18 Fore Cancer Research, USA |
| Givehope Pancreatic Cancer Research and Awareness Alliance, USA |
| Glenn Foundation for Medical Research, USA |
| Global Cancer Institute, USA |
| Global Oncology Inc., USA |
| Global Prostate Cancer Research Foundation Inc., USA |
| Gloria's Girls Foundation, USA |
| Godfrey Family Fund in memory of Fiona Penelope, USA |
| Gold In September (G9) Foundation, USA |
| Gold4Kids Cancer Foundation of Tulsa, USA |
| Goldhirsh Foundation, USA |
| Goldhirsh-Yellin Foundation, USA |
| Goldman Sachs Gives, USA |
| Goldwin Foundation, USA |
| Golfers Against Cancer, USA |
| Gordon and Betty Moore Foundation, USA |
| Grandmas Cancer Research Fund, USA |
| Greater Kansas City Community Foundation, USA |
| Greenwall Foundation, USA |
| Gregory Foundation for Cancer Research Inc., USA |
| Griffin's Guardians, USA |
| Gross-Loh Family Fund for Lung Cancer Research, USA |
| Grow for Life Breast Cancer Research and Education Foundation, USA |
| Gundersen Medical Foundation, USA |
| Gynecologic Cancer Research Foundation Inc., USA |
| H. Austin and Florence R.S. Kaye Foundation, USA |
| Hainan Cihang Foundation, USA |
| Hanna Family Research Chair in Surgical Oncology, USA |
| Harold and Virginia Lash Trusts, USA |
| Harry J. Lloyd Charitable Trust, USA |
| Health Research Inc., USA |
| Healthnetwork Foundation, USA |
| Healthwise, USA |
| Hearst Foundation, USA |
| Helen L. Kay Charitable Trust, USA |
| Helen Moss Breast Cancer Research Foundation, USA |
| Helen Rey Breast Cancer Research Fund, USA |
| Helmsley Charitable Trust, USA |
| Hematology Oncology Pharmacy Association Inc., USA |
| Henry J. Predolin Foundation, USA |
| Henry M. and Stella M. Hoenig Endowed Chair, USA |
| Henry M. Jackson Foundation, USA |
| HERA Women's Cancer Foundation, USA |
| Hercules Foundation, USA |
| Higgins Family Foundation, USA |
| Hillman Family Foundations, USA |
| Hippocratic Cancer Research Foundation Inc., USA |
| Hirshberg Foundation for Pancreatic Cancer Research, USA |
| Hitchcock Foundation, USA |
| Hoag Hospital Foundation, USA |
| Hodson Trust, USA |
| Hoosier Cancer Research Network Inc., USA |
| Hope Funds for Cancer Research, USA |
| Hope Promise the Charlie Glass Sarcoma Research Foundation, USA |
| Hope Street Kids, USA |
| Howard Hughes Medical Institute, USA |
| Hugh Simons in honor of Frank and Anne Simons, USA |
| Hyundai Hope On Wheels, USA |
| I Care I Cure Childhood Cancer Foundation, USA |
| Ians Friends Foundation, USA |
| Illinois Oncology Research Association, USA |
| Independent Cancer Research Foundation Inc., USA |
| Indo-American Cancer Association, USA |
| Inflammatory Breast Cancer Research Foundation, USA |
| Ingeborg Osterchrist Oncology Endowment Inc., USA |
| Innovative Advanced Cancer Research Foundation Inc., USA |
| Institute for Integrative Cancer Research and Education Inc., USA |
| Institute for Myeloma and Bone Cancer Research, USA |
| Intermountain Healthcare Foundation, USA |
| International Association for the Study of Lung Cancer - IASLC, USA |
| International Cardioncology Society of North America Inc., USA |
| International Lymphoma Radiation Oncology Foundation, USA |
| International Myeloma Foundation, USA |
| International Psycho-Oncology Society Inc., USA |
| International Society for Cutaneous Lymphomas, USA |
| International Society for Preventive Oncology Inc., USA |
| International Society of Gastrointestinal Oncology, USA |
| International Thymic Malignancy Interest Group - ITMIG, USA |
| Iowa Oncology Research Association, USA |
| Iris Foundation for Uterine Cancer Research Awareness, USA |
| Irma T. Hirschl Trust, USA |
| Irwin and Joan Jacobs Fund, USA |
| J&D Cancer Research and Treatment Development Foundation, USA |
| Jabboury Foundation for Cancer Research Inc., USA |
| Jack H Marston II Cancer Research Fund A Not for Profit Corp, USA |
| Jacqueline Seroussi Memorial Foundation for Cancer Research, USA |
| James E. Kearney Foundation, USA |
| James J. Leibman and Rita S. Leibman Endowment Fund for Cancer Research, USA |
| James Paul Sutton Medical Research Fund, USA |
| James S. McDonnell Foundation, USA |
| Jamie Rabinowitch-Davis Foundation for Melanoma Research Inc., USA |
| Janet Burros Memorial Foundation, USA |
| Janet Orelli Foundation for Breast Cancer Research, USA |
| Janice McArdle Cancer Research Foundation Inc., USA |
| Jared Branfman Sunflowers for Life Fund for Pediatric Brain and Spinal Cancer Research, USA |
| Jasper L. and Jack Denton Wilson Charitable Foundation, USA |
| Javacia W Harrison Respect Academy and Cancer Research Foundation, USA |
| Jean Perkins Foundation, USA |
| Jean Shanks Foundation, USA |
| Jeanne F. Shelby Scholarship Fund, USA |
| Jeff Butler Sarcoma Foundation, USA |
| Jeff Gordon Children's Foundation, USA |
| Jefferson Neblett Foundation for Pancreatic Cancer Research, USA |
| Jeffrey And Karen Peterson Family Foundation, USA |
| Jeffrey Pride Foundation for Pediatric Cancer Research, USA |
| Jeh Foundation for Pancreatic Cancer Research, USA |
| Jennifer Clanton Leptomeningeal Cancer Research Foundation, USA |
| Jennifer Hunter Yates Sarcoma Foundation, USA |
| Jewish General Hospital Foundation, USA |
| Jhpiego Corporation, USA |
| Jim and Christy Everest Endowed Chair in Cancer Developmental Therapeutics, USA |
| Joanna M Nicolay Melanoma Foundation Inc., USA |
| Joanna M. Nicolay Melanoma Foundation, USA |
| Joey and Mary Furfari Cancer Research Fund, USA |
| Joey Ramone Foundation for Lymphoma Research Inc., USA |
| John Estrella Foundation for Cancer Research, USA |
| John F Fortney Chartiable Pancreatic Cancer Research Group, USA |
| John P Hanson Foundation for Cancer Research Inc., USA |
| John S. Dunn Foundation, USA |
| John Templeton Foundation, USA |
| John Wawrynovic Leukemia Research Scholar Endowment, USA |
| Jonas Philanthropies, USA |
| Jonathan and Susan Wener Fund, USA |
| Jordan and Kyra Memorial Foundation, USA |
| Joseph A Laporta Neuroblastoma Cancer Foundation, USA |
| Joseph C. Monastra Foundation for Pancreatic Cancer Research, USA |
| Joseph D. Boyle Memorial Fund, USA |
| Joseph Lentz Fund for Pediatric Brain Cancer Research, USA |
| Joseph S. and Diane H. Steinberg Charitable Trust, USA |
| Josh Gottheil Memorial Fund for Lymphoma Research, USA |
| Judi A. Rees Ovarian Cancer Research Fund, USA |
| Jurgen Sager and Transocean Melanoma Research Fund, USA |
| Juvenile Diabetes Research Foundation - JDRF, USA |
| Kadet Cancer Research Foundation, USA |
| Kaleidoscope of Hope Ovarian Cancer Foundation, USA |
| Kanzius Cancer Research Foundation, USA |
| Karen Wyckoff Rein in Sarcoma, USA |
| Karin Grunebaum Cancer Research Foundation, USA |
| Kate Verdon Spisak Foundation for Melanoma Awareness & Research Inc., USA |
| Kathy Duffey Fogarty Fund, USA |
| Kavanagh Family Foundation, USA |
| Kavli Foundation, USA |
| Kazan McClain Partners' Foundation, USA |
| Keeling Family Foundation, USA |
| Kent Cancer Trust, USA |
| Kentuckiana Cancer Research Foundation Inc., USA |
| Kerri Castello Cancer Research Foundation Inc., USA |
| Kettering Radiation Oncology Foundation, USA |
| Kevin Mullin Memorial Fund, USA |
| Kevin's Fund, USA |
| Kicks4chris Foundation for Cancer Research, USA |
| Kidney Cancer Research Alliance, USA |
| Kids Cancer Alliance, USA |
| Kids Walk for Kids with Cancer, USA |
| Kim Walt Memorial Breast Cancer Research Fund, USA |
| King & Jennings National Synovial Sarcoma, USA |
| Kk-125 Ovarian Cancer Research Foundation, USA |
| Kochhar Cancer Research Foundation, USA |
| Koss National Triple Negative Breast Cancer Research Foundation, USA |
| L.E.W. Carty Charitable Fund, USA |
| Ladies Leukemia League, Inc. of the Gulf South Region, USA |
| Lake Champlain Cancer Research Organization, USA |
| Larry Burkett Cancer Research Foundation Inc., USA |
| Laura and John Arnold Foundation, USA |
| Laura Hinz Copeland Ewing Sarcoma Foundation Inc., USA |
| Lauren Ann Levy Foundation for Lymphoma & Cancer Research, USA |
| Leah's Happy Hearts!, USA |
| Lederach Cancer Research Inc., USA |
| Lee National Denim Day, USA |
| Lefkofsky Family Foundation, USA |
| Leo and Anne Albert Charitable Trust, USA |
| Leslie F Schwartz Pancreatic Cancer Research Foundation, USA |
| Leukemia and Lymphoma Society, USA |
| Leukemia Research Foundation, USA |
| Leukemia Research Foundation of Delaware, USA |
| Lewis Family Foundation, USA |
| Lilly Endowment, USA |
| Linda Horowitz Cancer Research Foundation, USA |
| Linda J Verville Cancer Research Foundation Inc., USA |
| Live Like Katie Foundation, USA |
| Livestrong Foundation, USA |
| Lmsarcoma Direct Research Foundation, USA |
| Lokanayagi Adikesavan Cancer Research Foundation, USA |
| Lolo's Angels, Inc., USA |
| Lon V Smith Foundation, USA |
| Longaberger's Horizon of Hope, USA |
| Lori Ann Breast Cancer Foundation, USA |
| Lori Groetken Foundation for Pancreatic Cancer Research Nfp, USA |
| Lou Malnati's Cancer Research Fund, USA |
| Louise Belley and Richard Schnarr Fund, USA |
| Louisiana Cancer Foundation, USA |
| Love Your Melon, USA |
| Lucius Wing Endowed Chair in Cancer Research and Therapy, USA |
| Ludwig Institute for Cancer Research, USA |
| Lung Cancer Research Council Inc., USA |
| LUNGevity Foundation, USA |
| Lustgarten Foundation for Pancreatic Cancer Research, USA |
| Lyda Hill Foundation, USA |
| Lymphoma Foundation of America, USA |
| Lymphoma Research Foundation, USA |
| Lynne Cohen Foundation for Ovarian Cancer Research, USA |
| M. Adnan Hamed Family, USA |
| Mackay Foundation for Cancer Research, USA |
| Macy Easom Cancer Research Foundation Inc., USA |
| Maine Cancer Foundation, USA |
| Maizie Holton Foundation for Lung Cancer Research, USA |
| Making Headway Foundation, USA |
| Manhattan Institute for Cancer Research, USA |
| Marc Apodaca Jr Childrens Glioma Cancer Foundation, USA |
| Marc Jacobs' Skin Cancer Awareness Campaign, USA |
| March of Dimes, USA |
| Margaret Harvey Schering Trust for Cancer Research, USA |
| Margaret Q. Landenberger Research Foundation, USA |
| Margaret W. Elkins Endowed Research Fund, USA |
| Margie and Robert E. Petersen Foundation, USA |
| Maria Frangella Foundation for Colon Cancer Research Inc., USA |
| Marianne Dinofrio Pancreatic Cancer Research Foundation, USA |
| Marie C Petrilli Cancer Research & Treatment Memorial Fund, USA |
| Marie H Lundin Memorial Test Tr FBO Cancer Research, USA |
| Marie-Josée and Henry R. Kravis Foundation, USA |
| Marilyn Augur Family Foundation, USA |
| Mario Lemieux Foundation, USA |
| Marissa Pogal-Sussman Mesenchymal Chondrosarcoma Research Foundation, USA |
| Marit Peterson Melanoma Research Fund, USA |
| Mark Encin Foundation for Melanoma Research, USA |
| Mary Blazer Fund for Ge Cancer Research, USA |
| Mary Jayne Casillo Foundation for Cancer Research and Awareness Inc., USA |
| Mary Jean Mitchell Green Foundation, USA |
| Mary K. Chapman Foundation, USA |
| Mary Kay Foundation, USA |
| Mary M. Halinski Pancreatic Cancer Research Fund, USA |
| Mary Weaver Foundation for Cancer Research, USA |
| Mason Chandler Allen Memorial Foundation, USA |
| Mater Foundation, USA |
| Matthew Lehrman Osteosarcoma Fund Inc., USA |
| Max Burdette Fibrolamellar Cancer Research Foundation, USA |
| Max of A Million Dreams Foundation for Cancer Research, USA |
| McIntyre Family Fund for Neuroendocrine Tumor Research, USA |
| McKenna Claire Foundation, USA |
| Melanoma Action Coalition Inc., USA |
| Melanoma Hope Network Inc., USA |
| Melanoma International Foundation, USA |
| Melanoma Know More, USA |
| Melanoma Miles for Mike, USA |
| Melanoma Research Alliance, USA |
| Melanoma Research Foundation, USA |
| Melanoma Research Foundation Breakthrough Consortium, USA |
| Melissa K Bambino Melanoma Foundation, USA |
| Melody Shafie Foundation for Neuroblastoma, USA |
| Melville Charitable Trust, USA |
| Merck Company Foundation, USA |
| Merck Family Fund, USA |
| Merediths Mission for Melanoma Inc., USA |
| Mesothelioma Applied Research Foundation, USA |
| Metastasis Research Society, USA |
| METAvivor Research and Support Inc., USA |
| Methuselah Foundation, USA |
| Meyer And Ida Gordon Foundation, USA |
| Mezin-Koats Colon Cancer Research, USA |
| Mia Ware Foundation for Cancer Research and Education, USA |
| Miami Foundation for Cancer Research Inc., USA |
| Michael and Karyn Goldstein Cancer Research Fund, USA |
| Michael and Susan Dell Foundation, USA |
| Michelle Paternoster Foundation for Sarcoma Research, USA |
| Michigan Melanoma Foundation, USA |
| Midwest Athletes Against Childhood Cancer - MACC Fund, USA |
| Midwest Melanoma Partnership, USA |
| Mike Geltrude Foundation, USA |
| Mildred V. Strouss Endowed Chair in Translational Research in Pediatric Oncology, USA |
| Miles Against Melanoma, USA |
| Miles Against Melanoma Oklahoma Foundation, USA |
| Milken Family Foundation, USA |
| Miller Family Foundation, USA |
| Minneapolis Medical Research Foundation, USA |
| Minnesota Colorectal Cancer Research Foundation, USA |
| Minnesota Masonic Charities, USA |
| Minnesota Medical Foundation, USA |
| Minnesota Ovarian Cancer Alliance, USA |
| Miranda D Beck Pediatric Cancer Research Foundation, USA |
| Mississippi Oncology Society Inc., USA |
| Mitsubishi Corporation Foundation for the Americas, USA |
| MLS Works, USA |
| Moffitt Cancer Center Foundation, USA |
| Mondelez International Foundation, USA |
| Morgan Adams Foundation, USA |
| Morningside Foundation, USA |
| Morris and Horowitz Families Endowed Professorship, USA |
| Mount Zion Health Fund, USA |
| Moving for Melanoma of Delaware Inc., USA |
| Multiple Myeloma Research Foundation, USA |
| Mulva Family Foundation, USA |
| Murphy Family Fund for Neuroendocrine Tumor Research, USA |
| Muscular Dystrophy Association, USA |
| Musella Foundation For Brain Tumor Research and Information, USA |
| Musicians Against Childhood Cancer - MACC, USA |
| Myra Shaw Cancer Research Fund Inc., USA |
| Mzb Center for Cancer Research Inc., USA |
| N8 Foundation, USA |
| Nadia's Gift Foundation, USA |
| Nancy and Stephen Grand Fund, USA |
| Nancy C. Cully Endowment for Leukemia Research, USA |
| Nancy Owens Breast Cancer Foundation, USA |
| National Brain Tumor Society, USA |
| National Cancer Center, Inc., USA |
| National Cancer Research Foundation Ltd., USA |
| National Colorectal Cancer Research Alliance, USA |
| National Comprehensive Cancer Network - NCCN, USA |
| National Foundation for Cancer Research - NFCR, USA |
| National Immunotherapy Cancer Research Foundation Inc., USA |
| National Leiomyosarcoma Foundation, USA |
| National Multiple Sclerosis Society, USA |
| National Organization for Rare Disorders - NORD, USA |
| National Ovarian Cancer Coalition, USA |
| National Pediatric Cancer Foundation, USA |
| National Philanthropic Trust, USA |
| National Youth Science Foundation, USA |
| Native American Cancer Research Corporation, USA |
| Natlie A Cole-Reagins Education and Cancer Research Foundation, USA |
| Nebraska Foundation for Cancer Research, USA |
| Nejat International Childhood Cancer Research Society, USA |
| Nemours Foundation, USA |
| Neuroblastoma Childrens Cancer Society, USA |
| Neuroendocrine Tumor Research Foundation, USA |
| Nevada Cancer Research Foundation Inc., USA |
| New Fund Institute for Collaborative Cancer Research Inc., USA |
| New Jersey Health Foundation, USA |
| New Mexico Society of Clinical Oncology, USA |
| New York Stem Cell Foundation, USA |
| Newman Family Foundation, Inc., USA |
| Nick Currey Fund, USA |
| Nick Reamer Osteosarcoma Foundation, USA |
| Nickols Miers Lundy Cancer Research Fund, USA |
| Nicole Meloche Memorial Breast Cancer Fund, USA |
| NRG Oncology Foundation, USA |
| Nw Sarcoma Foundation A Non-Profit Corporation, USA |
| Oak Foundation, USA |
| Ocala Royal Dames for Cancer Research Inc., USA |
| Ocala Royal Dames For Cancer Research, Inc., USA |
| Ocular Melanoma Foundation, USA |
| Ofelia Cancer Research Fund, USA |
| Ohio Cancer Research, USA |
| Ohio Lions Eye Research Foundation, USA |
| OHSU Foundation, USA |
| Oncology Consultants Cancer Research Fund Inc., USA |
| Oncology Foundation of Maryland and the District of Columbia Inc., USA |
| Oncology Nursing Foundation, USA |
| Oncology Nursing Society Foundation, USA |
| One-in-Six Fund, USA |
| Open Society Foundations, USA |
| Ora Lee Smith Cancer Research Foundation, USA |
| Oral and Maxillofacial Surgery Foundation - OMS Foundation, USA |
| Oregon Cancer Foundation, USA |
| Orion Research Foundation, USA |
| Osteoporosis and Breast Cancer Research Center, USA |
| Osteosarcoma Collaborative Inc., USA |
| Osteosarcoma Institute, USA |
| Otto Heinrich Warburg Cancer Research Foundation Inc., USA |
| Outpacing Melanoma Foundation, USA |
| Ovarian Cancer Alliance of Arizona, USA |
| Ovarian Cancer Research Fund Alliance, USA |
| Ovarian Cancer Research Inc., USA |
| Oxnard Foundation, USA |
| Pacific Otolaryngology Foundation, USA |
| Pacific Pediatric Neuro-Oncology Consortium Foundati, USA |
| Pacific Shores Hematology Oncology Foundation, USA |
| Painting the Town Gold, USA |
| Pancreatic Cancer Action Network, USA |
| Pancreatic Cancer Research Enterprise America, USA |
| Pano Koumantaros Cancer Research Fund Inc., USA |
| Papanicolaou Corps for Cancer Research, Inc., USA |
| Pappas Family Research Fund for Pancreatic Cancer, USA |
| Pastor Marlon Greggory Ritter Sr Foundation for Cancer Research, USA |
| Patel Memorial Breast Cancer Endowment Fund, USA |
| PATH, USA |
| Paul and Mary Haas Chair in Genetics, USA |
| Paul Esposito Foundation for Bile Duct & Liver Cancer Research, USA |
| Paul Nabil Bustany Memorial Fund for Synovial Sarcoma Research, USA |
| Paula and Russell Agrusa Fund for Colorectal Cancer Research, USA |
| Paula Takacs Foundation for Sarcoma Research, USA |
| Pauline Altman-Goldstein Foundation, USA |
| Pedals for Pediatrics, USA |
| Pediatric Brain Tumor Foundation, USA |
| Pediatric Cancer Foundation, USA |
| Pediatric Cancer Research Foundation, USA |
| Peggy Spiegler Melanoma Research Foundation, USA |
| Pelotonia, USA |
| Penn State Chocolate Tour, USA |
| Penny F Garrett Sarcoma Foundation, USA |
| Perry S. Levy Fund for Gastrointestinal Cancer Research, USA |
| Pershing Square Sohn Cancer Research Alliance, USA |
| Peter Latos Prostate Cancer Foundation, USA |
| Peter Michael Foundation, USA |
| Peter R. Leavitt Family Fund for GI Oncology Research, USA |
| Peter Skelton Sarcoma Research Foundation, USA |
| Pfizer Foundation, USA |
| Phalan Thoracic Gene Therapy Fund, USA |
| Phase One Foundation, USA |
| Phi Beta Psi Sorority, USA |
| Philip A Bryant Melanoma Foundation Inc., USA |
| Philippe Foundation Inc., USA |
| PhRMA Foundation, USA |
| Pine Tree Apple Tennis Classic, USA |
| Pink Ribbon International, USA |
| Pittsburgh Melanoma Foundation, USA |
| Polka Dot Mama Melanoma Foundation, USA |
| Presbyterian Health Foundation, USA |
| Press On Fund, USA |
| Prevent Cancer Foundation, USA |
| Preventive Oncology International, USA |
| Prezyna Research Oncology Initiative, USA |
| Primary Children's Hospital Foundation, USA |
| Professor Walter Morris-Hale Distinguished Chair in Urologic Oncology at Brigham and Women's Hospital, USA |
| Promises for Purple, USA |
| Prospect Creek Foundation, USA |
| Prostate Cancer Foundation, USA |
| Prostate Cancer Research and Education Foundation, USA |
| Prostate Cancer Research Institute, USA |
| Public Health Institute, USA |
| Pulenzas Cancer Research Fund, USA |
| Purple Promise Foundation to End Melanoma, USA |
| QuadW Foundation, USA |
| Queen of Hearts Foundation, USA |
| R Taub Cancer Research Fund Inc., USA |
| Race to Cure Lymphoma Incorporated, USA |
| Radiation Oncology Accelerated Research, USA |
| Radiation Oncology Institute, USA |
| Radiological Society of North America - RNSA, USA |
| Rally Foundation for Childhood Cancer Research, USA |
| Ralph C. Marcove Cancer Research Foundation, Inc., USA |
| Randy Shaver Cancer Research and Community Fund, USA |
| Rappaport Family Foundation, USA |
| Rare Cancer Research Foundation, USA |
| Ravi and Naina Patel Foundation, USA |
| Reed Gastrointestional Oncology Research Foundation, USA |
| Regis Foundation for Breast Cancer Research, USA |
| Research to Prevent Blindness, USA |
| Rexanna's Foundation for Fighting Lung Cancer, USA |
| Reza and Georgianna Khatib Endowed Chair in Skull Base Tumor Surgery, USA |
| Richard M. Lucas Cancer Foundation, USA |
| Richard M. Schulze Family Foundation, USA |
| Richard Spencer Lewis Memorial Foundation, USA |
| Riley Children's Foundation, USA |
| Ring Screw Textron Endowed Chair of Pediatric Cancer Research, USA |
| Rita Allen Foundation, USA |
| Rivkin Center for Ovarian Cancer Research, USA |
| Robert and Kate Niehaus Foundation, USA |
| Robert J Seifer Cancer Research Foundation, USA |
| Robert J. Kleberg, Jr. and Helen C. Kleberg Foundation, USA |
| Robert K. Steel Family Foundation, USA |
| Robert L Fine Cancer Research Laboratory Foundation Inc., USA |
| Robert M Hoffman Foundation for Cancer Research, USA |
| Robert Magnin Newman Endowed Chair in Neuro-oncology, USA |
| Robert O. and Annamae Orr Family Foundation, USA |
| Robert T. Hale Sr. and Judith B. Hale Fund for Pancreatic Cancer Research, USA |
| Robert W. Umbach Cancer Foundation, Inc., USA |
| Robert Wood Johnson Foundation, USA |
| Robertson Foundation, USA |
| Robin Page/Lebor Foundation, USA |
| Rochester Melanoma Action Group Inc., USA |
| Rock River Cancer Research Foundation Inc., USA |
| Rockefeller Foundation, USA |
| Roderick D. MacDonald Research Fund, USA |
| Rolfe Pancreatic Cancer Foundation, USA |
| Rombauer Pancreatic Cancer Research Fund, USA |
| Ronnie James Dio Stand Up and Shout Cancer Fund, USA |
| Rory David Deutsch Foundation, USA |
| Rosenberg Integrated Cancer Research Institute Inc., USA |
| Roswell Park Alliance Foundation, USA |
| Rotary International, USA |
| Roy J. Carver Charitable Trust, USA |
| Royal Dames of Cancer Research Inc., USA |
| Ruby Family Foundation, USA |
| Rudolf B. Becker Foundation, USA |
| Russell Hill Cancer Foundation, USA |
| Ruth N. White Research Fellowship in Gynecologic Oncology, USA |
| Ruth Sager Memorial Fund, USA |
| S D Ireland Cancer Research Fund Inc., USA |
| Salgi Esophageal Cancer Research Foundation, USA |
| Sally M. Kingsbury Sarcoma Research Foundation, USA |
| Sally Snowman Survivorship Fellowship, USA |
| Salvatore Ferrara II Esophageal Cancer Research Fund, USA |
| Sammy's Superheroes Foundation, USA |
| Samuel Waxman Cancer Research Foundation, USA |
| Samueli Foundation, USA |
| San Diego Cancer Research Institute, USA |
| San Diego Professionals Against Cancer, USA |
| Sandy Rollman Ovarian Cancer Foundation, USA |
| Sara Brown Musselman Fund for Serous Ovarian Cancer Research, USA |
| Sarcoma Alliance for Research Through Collaboration - SARC, USA |
| Sarcoma Foundation of America - SFA, USA |
| Sarcoma Help from the Liddy Shriver Sarcoma Initiative, USA |
| Sarcoma Research Foundation, USA |
| Sarcoma-Oma, USA |
| Schowalter Foundation, USA |
| Schwedler Family Foundation, USA |
| Scoliosis Research Society, USA |
| Scott Forbes and Gina Ventre Fund, USA |
| Scott Hamilton CARES Foundation, USA |
| Scotts Seafood Ucd Cancer Research Fund, USA |
| Sdh Cancer Research Advocates Inc., USA |
| Sealy and Smith Foundation, USA |
| Sean Brady Foundation for Cancer Research and Treatment, USA |
| Sentinel Node Oncology Foundation, USA |
| Sharon Crowley Martin Memorial Fund for Melanoma Research, USA |
| Sharon Strauss Parker Lymphoma Research Foundation Inc., USA |
| Shear Family Foundation, USA |
| Shelby Rae Tengg Foundation for Heart and Cancer Research, USA |
| Sheryl Brody Cancer Research Foundation, USA |
| Shivers Cancer Foundation, USA |
| Shurl and Kay Curci Foundation, USA |
| Sidney Kimmel Foundation, USA |
| Sierra Rayn Childrens Neuroblastoma Foundation Inc., USA |
| Sigma Beta Sorority, Inc., USA |
| Silicon Valley Community Foundation, USA |
| Simone Bender Cancer Research Fund Inc., USA |
| Simons Foundation, USA |
| Singleton Family Foundation, USA |
| Sino-American Cancer Foundation , USA |
| Skirball Foundation, USA |
| Sky Foundation, USA |
| Skyler Rain Barber Neuroblastoma Foundation, USA |
| Slay Sarcoma Research Initiative, USA |
| Small Cell Ovarian Cancer Foundation, USA |
| Smashing Walnuts, USA |
| Smiles for Lymphoma Inc., USA |
| Smoking Research Foundation, USA |
| Soccer For Hope Foundation, USA |
| Society for Immunotherapy of Cancer - SITC, USA |
| Society for Integrative Oncology Inc., USA |
| Society for Melanoma Research Ltd., USA |
| Society for Neuro-Oncology, USA |
| Society for Translational Oncology Inc., USA |
| Society of Gynecologic Oncology - SGO, USA |
| Society of Surgical Oncology, USA |
| Society of Urologic Oncology, USA |
| Society of Urologic Oncology Clinical Trials Consortium, USA |
| Sohn Conference Foundation, USA |
| Sol Goldman Charitable Trust, USA |
| Sondra and Stephen Hardis Chair in Oncology Research, USA |
| Sonya A. Sinicki Foundation for Cancer Research, Inc., USA |
| Soonmar Cancer Research Inc., USA |
| Souls of the Beach Foundation for Appendix Cancer Research Inc., USA |
| Soupy for Loopy Foundation, Inc., USA |
| South Florida Veterans Affairs Foundation for Research and Education, USA |
| Southeastern Brain Tumor Foundation, USA |
| Southern Teal Gynecological Oncology Research and Education Foundation, USA |
| Southwest Oncology Group - SWOG, USA |
| Speedway Children's Charities, USA |
| Sperling Family Charitable Foundation, USA |
| St Baldricks Foundation, USA |
| Stading-Younger Cancer Research Foundation, USA |
| Stand Up To Cancer, USA |
| Stanley D. Lewis and Virginia S. Lewis Endowed Chair in Brain Tumor Research, USA |
| Steelman Family Foundation, USA |
| Stefanie Spielman Fund for Breast Cancer Research, USA |
| Step Up for Sarcoma, USA |
| Stephen L. and Lavinia Boyd Fund for Leukemia Research, USA |
| Steve Van Andel Foundation, USA |
| Steven G Aya Cancer Research Fund, USA |
| Steven Gordon Family Foundation, USA |
| Stick It to Sarcoma Corp, USA |
| Stop Cancer Foundation , USA |
| Suns Cancer Research Foundation, USA |
| Susan and Peter Solomon Divisional Genomics Program, USA |
| Susan Fazio Foundation for Melanoma Research, USA |
| Susan G. Komen Breast Cancer Foundation, USA |
| Susan Spooner Family Lung Cancer Research Fund, USA |
| Swim across America, USA |
| Syracuse Cancer Research Institute Inc., USA |
| T.J. Martell Foundation, USA |
| Tabar Breast Cancer Research and Education Foundation, USA |
| Tara Miller Melanoma Foundation, USA |
| Tawny K Dahring Foundation for Cancer Research, USA |
| T-Cell Leukemia Lymphoma Foundation, USA |
| Team Jack Foundation, USA |
| Team Luke Versus Neuroblastoma A Nj Nonprofit Corporation, USA |
| Team Ryan Charitable Foundation for Pediatric Cancer Research, USA |
| Teed Off at Cancer, USA |
| Terri Brodeur Breast Cancer Foundation, USA |
| Texas Oncology Foundation Inc., USA |
| The William and Flora Hewlett Foundation, USA |
| The Abney Foundation, USA |
| The Abraham J. and Phyllis Katz Foundation, USA |
| The Alexander Family Foundation, USA |
| The Alvin H. Baum Family Fund, USA |
| The Assisi Foundation of Memphis, USA |
| The Atlantic Philanthropies, USA |
| The Baker Street Foundation, USA |
| The Beez Foundation, USA |
| The Ben and Catherine Ivy Foundation, USA |
| The Bonnie J. Addario Lung Cancer Foundation, USA |
| The Breast Cancer Research Foundation of Alabama, USA |
| The Brewster Education Foundation, USA |
| The Cancer Institute Foundation, Inc., USA |
| The Carol Gollob Foundation for Breast Cancer Research, USA |
| The ChadTough Foundation, USA |
| The Children's Cancer Foundation, Inc., USA |
| The Cholangiocarcinoma Foundation, USA |
| The Cisco Foundation, USA |
| The Claudia Cohen Cancer Research Fund, USA |
| The Clearity Foundation, USA |
| The Cleveland Foundation, USA |
| The Coleman Foundation, USA |
| the Colt Foundation, USA |
| The Combat for a Cure Foundation, USA |
| The Cristian Rivera Foundation, USA |
| The Cure It Foundation, USA |
| The Cure Starts Now Foundation, USA |
| The David S. and Karen A. Shapira Foundation, USA |
| The Dennis Szefel Memorial Golf Tournament, USA |
| The Derfner Foundation, USA |
| The Devlin Fund for Cancer Research, USA |
| The Dorian J. Murray Foundation, USA |
| The Eli and Edythe Broad Foundation, USA |
| The Elias Foundation, USA |
| The Elias, Genevieve and Georgianna Atol Charitable Trust, USA |
| The Ernest B Albat and Rose A Albat Foundation for Cancer Research, USA |
| The Eshelman Foundation, USA |
| The EVAN Foundation, USA |
| The Eye Cancer Foundation, USA |
| The Fletcher Jones Foundation, USA |
| The Flora and Stuart Mason Lung Cancer Research Fund, USA |
| The Foundation for Barnes-Jewish Hospital, USA |
| The Frank A. Campini Foundation, USA |
| The Franklin Foundation for Innovation, USA |
| The Frazier Family Foundation , USA |
| The Fund for Blood and Cancer Research Inc., USA |
| The Garrett B. Smith Foundation, USA |
| The Geaton and Joann Decesaris Family Foundation, Inc., USA |
| The Gloria Borges WunderGlo Foundation, USA |
| The Greenberg Breast Cancer Research Foundation Inc., USA |
| The H.N. and Frances C. Berger Foundation, USA |
| The Harold C. Daily and Mary L. Daily Living Trust - The Harold C. and Mary L. Daily Endowment Fellowship, USA |
| The Harry Frank Guggenheim Foundation, USA |
| The Hartwell Foundation, USA |
| The Hastings Fund, USA |
| The Heed Ophthalmic Foundation, USA |
| The Hildegardo E. and Olga M. Flores Foundation, USA |
| The Homer Flower Gene Therapy Fund, USA |
| The Honorable Tina Brozman Foundation, USA |
| The Hope Foundation, USA |
| The Hormel Foundation, USA |
| The Immunotherapy Foundation, USA |
| The Jack and Dorothy Byrne Foundation, USA |
| The Jacquie L Goldman Brain Cancer Research Foundation, USA |
| The Jake Wetchler Foundation, USA |
| The Jason and Priscilla Hiley Fund, USA |
| The Joanne and John Dallepezze Foundation, USA |
| The Joey Fabus Childhood Cancer Foundation, USA |
| The John A. Hartford Foundation, USA |
| The Joseph C Monastra Foundation for Pancreatic Cancer Research, USA |
| The Julie Fund, USA |
| The Kansas University Endowment Association, USA |
| The Kenneth Stanton Fund, USA |
| The Kristen Ann Carr Fund, USA |
| The LAM Foundation, USA |
| The Laurel Foundation, USA |
| The Lawrence Ellison Foundation, USA |
| The Leon Levine Foundation, USA |
| The Leonard and Madlyn Abramson Family Cancer Research Institute, USA |
| The Lhakpa Gelu Foundation, USA |
| The Life Raft Group, USA |
| The Lily's Garden Endowment in Childhood Cancer, USA |
| The Lincy Foundation, USA |
| The Litman Family Fund for Cancer Research, USA |
| The Lori and Alan S. Zekelman Fund, USA |
| The Louis Feil Charitable Lead Annuity Trust, USA |
| The Lung Cancer Research Foundation, USA |
| The Lymphoma Foundation, USA |
| The Lynn Sage Foundation, USA |
| The M.S. Hershey Foundation - Four Diamonds Fund, USA |
| The Marcus Niziak Childhood Brain Tumor Fund, USA |
| The Marisa Fund, USA |
| The Mark Foundation for Cancer Research US Ltd., USA |
| The Matthew Larson Foundation, USA |
| The McCombs Foundation, USA |
| The Meyer Steinberg Foundation for Lung Cancer Research, Inc., USA |
| The Mike Slive Foundation for Prostate Cancer Research, USA |
| The Milburn Foundation, USA |
| The Mulva Family Foundation, USA |
| The National Coalition for Cancer Research, USA |
| The National Pancreas Foundation, USA |
| The Neuroblastoma Children's Cancer Society, USA |
| The New York Community Trust, USA |
| The Obesity Society, USA |
| The Oral Cancer Foundation, USA |
| The Pablove Foundation, USA |
| The Parker Foundation, USA |
| The Parker Foundation - Parker Institute for Cancer Immunotherapy, USA |
| The Patrick C. Walsh Prostate Cancer Research Fund, USA |
| The Pew Charitable Trusts, USA |
| The Philadelphia Foundation, USA |
| The Phoenix Friends of the University of Arizona Cancer Center, USA |
| The Pittsburgh Foundation, USA |
| The Plastic Surgery Foundation, USA |
| The Race Against Breast Cancer, USA |
| The Rebecca Susan Buffett Foundation, USA |
| The Ressler Family Foundation, USA |
| The RGK Foundation, USA |
| The Robert A. Welke Cancer Research Foundation, USA |
| The Robert and Deborah First Fund, USA |
| The Robert and Janice McNair Foundation, USA |
| The Rutledge Foundation, USA |
| The Sally Edelman and Harry Gardner Cancer Research Foundation Inc., USA |
| The Samuel Szabo Foundation, USA |
| The Sands Family Foundation, Inc., USA |
| The Scarlett Fund, USA |
| The Seany Foundation, USA |
| The Seaver Institute, USA |
| The Selikoff Fund for Environmental & Occupational Cancer Research Inc, USA |
| The Seraph Foundation, USA |
| The Skin Cancer Foundation, USA |
| The Skip Viragh Foundation, Inc., USA |
| The Society of Memorial Sloan Kettering Cancer Center, USA |
| The Sontag Foundation, USA |
| The St. Louis Men's Group Against Cancer, USA |
| The Starr Foundation, USA |
| The Stephen Gottlieb Memorial Fund for Cancer Research Inc., USA |
| The Super Jake Foundation, USA |
| The Susan Thompson Buffet Foundation, USA |
| The Thompson Family Foundation, USA |
| The Ty Louis Campbell Foundation, USA |
| The University of Miami Cancer Research Center, USA |
| The University of Oulu Scholarship Foundation, USA |
| The Virginia and D. K. Ludwig Fund for Cancer Research, USA |
| The W.K. Kellogg Foundation, USA |
| The Walter S. and Lucienne B. Driskill Foundation, USA |
| The Welch Foundation, USA |
| The Wenner-Gren Foundation, USA |
| The William C. Liedtke, Jr. Chair in Cancer Research, USA |
| The WWWW (Quad W) Foundation, USA |
| The Zarrow Family Foundations, USA |
| The Zell Family Foundation, USA |
| Theodora B. Betz Foundation, USA |
| ThinkCure! , USA |
| This Close for Cancer Research, USA |
| Thomas and Carol Cracchiolo Foundation, USA |
| Thomas M Hohman Memorial Cancer Research Fund, USA |
| Thrasher Research Fund, USA |
| Tim Nesvig Lymphoma Fellowship and Research Fund, USA |
| Timothy Aycock Melanoma Research Foundation, USA |
| Ting Tsung and Wei Fong Chao Foundation, USA |
| Tippins Foundation, USA |
| Tommy Detesco Fund for Brain Cancer Research, USA |
| Tony and Renee Marlon Charitable Foundation, USA |
| Tonys Prostate Cancer Research, USA |
| Tough Like Ike, USA |
| Tower Cancer Research Foundation, USA |
| Tracy Jo Wilson Ovarian Cancer Foundation, USA |
| Translational Research in Oncology US Inc., USA |
| Tsuruo Takashi Memorial Fund, USA |
| Tuberous Sclerosis Alliance, USA |
| Ufcw Leukemia Lymphoma Charity Golf Foundation, USA |
| United Breast Cancer Research Foundation, USA |
| United Cancer Research Institute, USA |
| United States Cutaneous Lymphoma Consortium, USA |
| United States-Israel Binational Science Foundation, USA |
| Uniting Against Lung Cancer, USA |
| University of Alberta Hospital Foundation, USA |
| University of Chicago Cancer Research Foundation, USA |
| University of Hawaii Foundation, USA |
| University of Minnesota - Minnesota Agricultural Experiment Station, USA |
| University of Minnesota Foundation, USA |
| University of Nebraska Foundation, USA |
| University of South Carolina Upstate Capital Development Foundation, USA |
| University of Tennessee West Institute for Cancer Research, USA |
| Unorthodox Philanthropy, USA |
| Unravel Pediatric Cancer, USA |
| Urological Research Foundation, USA |
| Urology Care Foundation, USA |
| V Foundation for Cancer Research, USA |
| Val Skinner Foundation, USA |
| Valda and Robert Svendsen Foundation, USA |
| Van Andel Research Institute - VARI, USA |
| Van Stephenson Memorial Cancer Research Fund Inc., USA |
| Vattikuti Foundation, USA |
| Verelst Foundation for Endometrial Cancer, USA |
| Vhl Cancer Research Foundation, USA |
| Viral Cancer Research Foundation, USA |
| Voices Against Brain Cancer, USA |
| W.M. Keck Foundation, USA |
| W.W. Smith Charitable Trust, USA |
| Walker Cancer Research Institute Inc., USA |
| Walking with Jane, USA |
| Wallace H. Coulter Foundation, USA |
| Wallace Research Foundation, USA |
| Walther Cancer Foundation, USA |
| Walton Family Foundation, USA |
| Wanda Bilec Foundation for Pancreatic Cancer Research, USA |
| Warren Brown Family Foundation, USA |
| Warren Cancer Research Foundation, USA |
| Washington Research Foundation, USA |
| Wayland's Warriors, USA |
| Wayne Fusaro Memorial Pancreatic Cancer Research Fund, USA |
| Weinman Foundation Fund, USA |
| Wendy English Cancer Research Foundation Ltd., USA |
| Wendy Feuer Fund for the Prevention and Treatment of Ovarian Cancer, USA |
| Wendy Walk for Sarcoma Research and Education, USA |
| Wendy Will Case Cancer Fund, Inc., USA |
| Werner & Lucie Picard Memorial Cancer Research Fund, USA |
| Werner and Lucie Picard Memorial Cancer Research Fund, USA |
| Wesley Coyle Memorial Fund, USA |
| Weston Havens Foundation, USA |
| Wheels for Prostate Cancer Research Inc., USA |
| Whittier Community Foundation, USA |
| William and Ella Owens Medical Research Foundation, USA |
| William Guy Forbeck Research Foundation, USA |
| William Lawrence and Blanche Hughes Foundation, USA |
| Wings of Hope for Pancreatic Cancer Research, USA |
| Wings Over Leukemia and Lymphoma Inc., USA |
| Wipe Out Kids Cancer, USA |
| Wiseman Cancer Research Foundation, USA |
| Women's Cancer Research Fund, USA |
| Wood Family Foundation, USA |
| Wood Hudson Cancer Research Laboratory Inc., USA |
| Young Texans Against Cancer, USA |
| Zelda Dorin Tetenbaum Memorial Fund, USA |
| Zero Club Research Fund, USA |
| Ziering Family Foundation in memory of Sigi Ziering, USA |
| Ziggy Marley George Foundation for Cancer Research and Treatment, USA |
| Anti-Cancer Society of Venezuela, Venezuela |
| Venezuelan Breast Cancer Research and Education Foundation, Venezuela  **Private for-profit**   \| Humanigen, Australia \| \| --- \| \| ImpediMed Limited, Australia \| \| Kazia Therapeutics, Australia \| \| Mesoblast Ltd, Australia \| \| Perpetual Ltd., Australia \| \| Prescient Therapeutics, Australia \| \| Sirtex Medical, Australia \| \| Viralytics, Australia \| \| Apeiron Biologics, Austria \| \| Biomay AG, Austria \| \| ASIT biotech, Belgium \| \| Biocartis SA, Belgium \| \| MDxHealth, Belgium \| \| Oncurious, Belgium \| \| Trod Medical, Belgium \| \| AngioChem, Canada \| \| Cotinga Pharmaceuticals, Canada \| \| GenomeDx, Canada \| \| Helix BioPharma, Canada \| \| Immunovaccine, Canada \| \| Jubilant DraxImage, Canada \| \| Oncolytics Biotech, Inc., Canada \| \| OncoQuest, Canada \| \| Sierra Oncology, Canada \| \| Trillium Therapeutics, Canada \| \| Zymeworks, Canada \| \| Andes Biotechnologies, Chile \| \| 3D Medicines, China \| \| Alphamab, China \| \| BeiGene, China \| \| Birdie Biopharmaceuticals, China \| \| CSPC ZhongQi Pharmaceutical, China \| \| Hengrui Therapeutics, China \| \| Hutchison MediPharma, China \| \| Jiangsu Hansoh Pharmaceutical Co., Ltd., China \| \| Jiangsu Hengrui Medicine, China \| \| Livzon Pharmaceutical, China \| \| Luye Pharma, China \| \| MabSpace Biosciences, China \| \| Shanghai Junshi Biosciences, China \| \| Shanghai Zhongxi Pharmaceutical (Group) Co., Ltd., China \| \| Sichuan Huiyang, China \| \| Suzhou Kintor Pharmaceuticals, China \| \| Zhejiang DTRM Biopharma, China \| \| Sotio, Czech Republic \| \| ALK-Abelló, Denmark \| \| Bavarian Nordic, Denmark \| \| Fabrikant Einar Willumsens Mindelegat, Denmark \| \| Genmab, Denmark \| \| Leo Pharma, Denmark \| \| Lundbeck, Denmark \| \| Novo Nordisk, Denmark \| \| Symphogen, Denmark \| \| FKD Therapies Oy, Finland \| \| Orion Corporation, Finland \| \| TILT Biotherapeutics Ltd., Finland \| \| AB Science, France \| \| Advanced Accelerator Applications, France \| \| Ascenta Therapeutics, France \| \| DBV Technologies SA, France \| \| Electricité de France - EDF, France \| \| GamaMabs Pharma, France \| \| Guerbet, France \| \| Hybrigenics, France \| \| Innate Pharma, France \| \| Institut Mérieux, France \| \| Ipsen Group, France \| \| Mutuelle Generale de l'Education Nationale - Mgen, France \| \| Nanobiotix, France \| \| OSE Immunotherapeutics, France \| \| Pierre Fabre, France \| \| Sanofi, France \| \| Servier, France \| \| Société 3M, France \| \| Affimed, Germany \| \| Apogenix AG, Germany \| \| Bayer Healthcare Pharmaceuticals LLC, Germany \| \| Bencard allergy GmbH, Germany \| \| Biotest, Germany \| \| Boehringer Ingelheim, Germany \| \| Brainlab, Germany \| \| Carl Zeiss Meditec, Germany \| \| Cellgenix Gmbh, Germany \| \| Fresenius Biotech, Germany \| \| Grünenthal Group, Germany \| \| Heidelberg Pharma AG, Germany \| \| Immatics Biotechnologies GmbH, Germany \| \| iOMEDICO AG, Germany \| \| MEDAC, Germany \| \| Merck - Allergopharma, Germany \| \| Merck Group, Germany \| \| Miltenyi Biotec, Germany \| \| Mologen, Germany \| \| MorphoSys, Germany \| \| Neovii Biotech GmbH, Germany \| \| Novartis - Hexal AG, Germany \| \| Onkovis, Germany \| \| Qiagen, Germany \| \| ROXALL Medizin GmbH, Germany \| \| Sandoz, Germany \| \| Schering AG, Germany \| \| Schering-Plough, Germany \| \| Siemens Healthineers, Germany \| \| SymbioPharm, Germany \| \| Sysmex Inostics, Germany \| \| Glenmark Pharmaceuticals, India \| \| Intas Biopharmaceuticals, India \| \| Olympus Therapeutics, India \| \| Sun Pharma Advanced Research, India \| \| Allergan, Ireland \| \| Jazz Pharmaceuticals, Ireland \| \| Medtronic, Ireland \| \| Shire, Ireland \| \| UDG Healthcare, Ireland \| \| BioLineRx, Israel \| \| Can-Fite BioPharma, Israel \| \| Gamida Cell Ltd., Israel \| \| Immunovative Therapies, Israel \| \| Silenseed, Israel \| \| Teva Pharmaceutical Industries Ltd., Israel \| \| VBL Therapeutics, Israel \| \| Alfasigma, Italy \| \| Chiesi Farmaceutici S.p.A., Italy \| \| Gentium, Italy \| \| Menarini, Italy \| \| MolMed, Italy \| \| Philogen, Italy \| \| Anaeropharma Science, Japan \| \| Astellas Pharma, Japan \| \| BrightPath Biotherapeutics, Japan \| \| CanBas, Japan \| \| Chugai Pharmaceutical Co., Japan \| \| Daiichi Sankyo, Japan \| \| Delta-Fly Pharma, Japan \| \| Eisai Co., Japan \| \| FUJIFILM Holdings - Toyama Chemical Co., Ltd., Japan \| \| Hutchison Whampoa Limited, Japan \| \| Kyowa Hakko Kirin Co., Ltd., Japan \| \| Kyowa Kirin Pharmaceutical Research, Inc, Japan \| \| Meiji Seika Pharma Co., Ltd., Japan \| \| Mitsubishi Chemical Holdings Corporation - Mitsubishi Tanabe Pharma Corporation, Japan \| \| Mochida Pharmaceutical Co., Ltd, Japan \| \| Momotaro-Gene, Japan \| \| NanoCarrier Co., Ltd., Japan \| \| NICHIAS Corporation, Japan \| \| Nihon Medi-Physics Co.,Ltd., Japan \| \| Nippon Boehringer Ingelheim Co., Ltd., Japan \| \| Nippon Kayaku Co., Ltd., Japan \| \| Oncolys BioPharma, Japan \| \| OncoTherapy Science, Inc., Japan \| \| Ono Pharmaceutical Co., Ltd., Japan \| \| Otsuka Pharmaceutical, Japan \| \| Shionogi, Japan \| \| Solasia Pharma, Japan \| \| Sumitomo Dainippon Pharma, Japan \| \| Sysmex Corporation, Japan \| \| Taiho Pharmaceutical, Japan \| \| Takeda Pharmaceutical, Japan \| \| Toray, Japan \| \| Tsumura and Co., Japan \| \| Yakult Honsha Co., Ltd., Japan \| \| Steba Biotech, Luxembourg \| \| Agendia, Netherlands \| \| Artu Biologicals Europe, Netherlands \| \| HAL Allergy Group, Netherlands \| \| Isodose Control B.V., Netherlands \| \| Kiadis Pharma, Netherlands \| \| Merus, Netherlands \| \| Nucletron B.V., Netherlands \| \| Philips Healthcare, Netherlands \| \| BerGenBio, Norway \| \| Nordic Nanovector, Norway \| \| Nycomed, Norway \| \| Photocure, Norway \| \| Targovax, Norway \| \| Macropharma Corporation, Philippines \| \| Captor Therapeutics, Poland \| \| Selvita, Poland \| \| Incuron, LLC, Russia \| \| ASLAN Pharmaceuticals, Singapore \| \| Tessa Therapeutics, Singapore \| \| BioMedSyn, South Korea \| \| Celltrion Healthcare, South Korea \| \| Enzychem Lifesciences, South Korea \| \| Green Cross Pharma, South Korea \| \| Hamni Pharmaceuticals, South Korea \| \| JW Pharmaceutical, South Korea \| \| MedPacto, South Korea \| \| Samsung Bioepis, South Korea \| \| SillaJen Biotherapeutics, South Korea \| \| Stcube Pharmaceuticals, South Korea \| \| BIAL-Industrial Farmacéutica SA, Spain \| \| Grupo J Uriach S.L., Spain \| \| Hartington Pharmaceutical, Spain \| \| Palobiofarma, Spain \| \| PharmaMar, Spain \| \| AFA Insurance, Sweden \| \| BioInvent International, Sweden \| \| C-rad, Sweden \| \| Elekta, Sweden \| \| Immunicum, Sweden \| \| Lokon Pharma, Sweden \| \| Meda Pharmaceuticals Inc., Sweden \| \| Medivir, Sweden \| \| Oncopeptides, Sweden \| \| Pharmacia and Upjohn, Sweden \| \| RaySearch Laboratories, Sweden \| \| Swedish Orphan Biovitrum - SOBI, Sweden \| \| Vivolux, Sweden \| \| Actelion, Switzerland \| \| Anergis SA, Switzerland \| \| Basilea Pharmaceutica, Switzerland \| \| Debiopharm, Switzerland \| \| Ferring Pharmaceuticals, Switzerland \| \| Galderma, Switzerland \| \| Helsinn Healthcare SA, Switzerland \| \| Hoffman-la-Roche Inc. - ROCHE, Switzerland \| \| Kuros BioSciences, Switzerland \| \| Molecular Partners, Switzerland \| \| Nestlé Global, Switzerland \| \| Nestlé Health Science - Prometheus Laboratories Inc., Switzerland \| \| Novartis AG, Switzerland \| \| Polyphor, Switzerland \| \| Rhizen Pharmaceuticals, Switzerland \| \| Vifor Pharma, Switzerland \| \| Golden Biotechnology, Taiwan \| \| OBI Pharma, Taiwan \| \| PharmaEssentia, Taiwan \| \| Senhwa Biosciences, Taiwan \| \| Synermore Biologics, Taiwan \| \| TaiRx, Taiwan \| \| Taiwan Liposome Company, Taiwan \| \| Teclison, Taiwan \| \| Acacia Pharma Ltd., United Kingdom \| \| Acerta Pharma, United Kingdom \| \| Allergy Therapeutics, United Kingdom \| \| Archigen Biotech, United Kingdom \| \| Archimedes Pharma, United Kingdom \| \| Astex Pharmaceuticals, United Kingdom \| \| Biocare Ltd, United Kingdom \| \| Circassia Pharmaceuticals, United Kingdom \| \| F-star Biotechnology, United Kingdom \| \| GE Healthcare Worldwide, United Kingdom \| \| GlaxoSmithKline, United Kingdom \| \| Immunocore, United Kingdom \| \| Karus Therapeutics, United Kingdom \| \| Minimally Invasive Therapies, United Kingdom \| \| Mission Therapeutics Ltd., United Kingdom \| \| Norgine, United Kingdom \| \| Novocure, United Kingdom \| \| Scancell Ltd., United Kingdom \| \| Stallergenes Greer, United Kingdom \| \| Sterix Ltd, United Kingdom \| \| Thermo Fisher Scientific, United Kingdom \| \| 21st Century Oncology, USA \| \| 2X Oncology, USA \| \| 3-V Biosciences, USA \| \| Aadi Bioscience, Inc., USA \| \| Abbott Laboratories, USA \| \| AbbVie, Inc., USA \| \| AbGenomics, USA \| \| Abraxis Bioscience, USA \| \| Acceleron Pharma, USA \| \| Accord Healthcare, USA \| \| Accuray, Inc., USA \| \| ACEA Biosciences, USA \| \| Actinium Pharmaceuticals, USA \| \| Actuate Therapeutics, USA \| \| Adaptimmune Therapeutics, USA \| \| Adaptive Biotechnologies, USA \| \| ADC Therapeutics, USA \| \| Adgero Biopharmaceuticals, USA \| \| Adlai Nortye, USA \| \| Aduro Biotech, USA \| \| Advantagene, USA \| \| Advaxis, USA \| \| Advenchen Laboratories, USA \| \| Aeglea Biotherapeutics, USA \| \| Agenus, USA \| \| Agios Pharmaceuticals, USA \| \| Aileron Therapeutics, USA \| \| AiVita Biomedical, USA \| \| Akeso Biopharma, USA \| \| Alexion Pharmaceuticals, USA \| \| Alexo Therapeutics, USA \| \| Alkermes, USA \| \| Allogene Therapeutics, USA \| \| Allos Therapeutics Inc, USA \| \| Alnylam Pharmaceuticals, USA \| \| Alopexx Oncology, USA \| \| Altor BioScience, USA \| \| Ambrx, USA \| \| Ambry Genetics, USA \| \| Ameritox, USA \| \| Amgen Inc., USA \| \| Amphivena Therapeutics, USA \| \| Angimmune, USA \| \| Angioblast Systems Inc., USA \| \| Angiodynamics, USA \| \| Annias Immunotherapeutics, USA \| \| Antigen Express, USA \| \| Apexian Pharmaceuticals, USA \| \| Apexigen, USA \| \| Apobiologix, USA \| \| Apotex, USA \| \| Aptevo Therapeutics, USA \| \| Aquestive Therapeutics, USA \| \| Aravive Biologics, USA \| \| Arch Oncology, USA \| \| Arcus Biosciences, USA \| \| Argos Therapeutics, USA \| \| Ariad Pharmaceuticals, USA \| \| ARMO Biosciences, USA \| \| AROG Pharmaceuticals, USA \| \| Arqule, USA \| \| Array Biopharma, USA \| \| Asana BioSciences, USA \| \| Ascentage Pharma, USA \| \| Aspyrian Therapeutics, USA \| \| Asterias Biotherapeutics, USA \| \| Astrazeneca, USA \| \| Atara Biotherapeutics, USA \| \| Athenex, USA \| \| Atossa Genetics, USA \| \| Augmenix, USA \| \| Aura Biosciences, USA \| \| Aurora BioPharma, USA \| \| Autotelic, USA \| \| Aveo Oncology, USA \| \| Avid Bioservices, USA \| \| Baxter International, USA \| \| Baylor Scott and White Health, USA \| \| Beckman Coulter, USA \| \| Berg, USA \| \| Betta Pharmaceuticals Co., Ltd, USA \| \| Bexion Pharmaceuticals, USA \| \| BeyondSpring Pharmaceuticals, USA \| \| BHR Pharma, USA \| \| BioAtla, USA \| \| Biocancell Therapeutic, Inc., USA \| \| BioClin Therapeutics, Inc., USA \| \| Biogen, USA \| \| BioMarck Pharmaceuticals, USA \| \| Biomarin, USA \| \| BioMed Valley Discoveries, USA \| \| Biomedical Sciences Institute, USA \| \| BioMimetix, USA \| \| Bioniz, USA \| \| BioNumerik Pharmaceuticals, USA \| \| Bio-Path Holdings, USA \| \| Biostage, USA \| \| Biothera Pharmaceuticals, Inc., USA \| \| Biovex, USA \| \| BioXcel Therapeutics, USA \| \| Bluebird Bio, USA \| \| BlueLink Pharmaceuticals, USA \| \| Blueprint Medicines, USA \| \| Boston Biomedical, USA \| \| Boston Scientific, USA \| \| BriaCell Therapeutics, USA \| \| Bristol Myers Squibb, USA \| \| Calithera Biosciences, USA \| \| Cancer Advances, USA \| \| Cancer Prevention Pharmaceuticals, USA \| \| Cantex Pharmaceuticals, USA \| \| Cao Pharmaceuticals, USA \| \| CaridianBCT Biotechnologies, USA \| \| Caris Life Sciences, USA \| \| CASI Pharmaceuticals, USA \| \| Castle Biosciences, USA \| \| Caterpillar, Inc., USA \| \| CBA Pharma, USA \| \| CBT Pharmaceuticals, USA \| \| CDG Therapeutics, USA \| \| Celgene, USA \| \| Celgene - Juno Therapeutics, USA \| \| Cell Medica, USA \| \| Celldex Therapeutics Inc, USA \| \| Cellectar Biosciences, Inc., USA \| \| Cellectis, USA \| \| Cellular Biomedicine, USA \| \| Cellular Dynamics International, Inc., USA \| \| CEL-SCI, USA \| \| Celsion, USA \| \| Celularity, USA \| \| Celyad, USA \| \| Cephalon Inc., USA \| \| Cepheid Inc., USA \| \| CerRx, USA \| \| Cerulean Pharma Inc., USA \| \| Cerus Corporation, USA \| \| Chan Zuckerberg Initiative, USA \| \| Checkmate Pharmaceuticals, USA \| \| Checkpoint Therapeutics, USA \| \| ChemoCentryx, USA \| \| Chimerix Inc., USA \| \| Churchill Pharmaceuticals, USA \| \| CicloMed, USA \| \| Cleave Biosciences, USA \| \| Clinigen Healthcare, USA \| \| Clovis Oncology, USA \| \| Cold Genesys, Inc., USA \| \| Colgate-Palmolive, USA \| \| Constellation Pharmaceuticals, USA \| \| Context Therapeutics, USA \| \| Corcept Therapeutics, USA \| \| Cortice Biosciences, USA \| \| Cougar Biotechnology Inc., USA \| \| CSL Behring, USA \| \| CTI BioPharma, USA \| \| Curegenix, USA \| \| CureLab Oncology, USA \| \| Curis, USA \| \| Cyclacel Pharmaceuticals, USA \| \| CytImmune Sciences, USA \| \| Cytocom, USA \| \| CytomX Therapeutics, USA \| \| Cytori Therapeutics, USA \| \| Cytovia, USA \| \| CytRx, USA \| \| Dauntless Pharmaceuticals, USA \| \| Deciphera Pharmaceuticals, Inc., USA \| \| Dekk-Tec, USA \| \| Delcath Systems, USA \| \| DelMar Pharmaceuticals, USA \| \| Dendreon, USA \| \| Denovo Biopharma, USA \| \| Depuy Synthes Companies, USA \| \| DepYmed, USA \| \| Diffusion Pharmaceuticals, USA \| \| DNAtrix, USA \| \| Dynavax Technologies, USA \| \| Eagle Pharmaceuticals, USA \| \| eFFECTOR Therapeutics, USA \| \| Eleison Pharmaceuticals, USA \| \| Eleven Biotherapeutics, USA \| \| Eli Lilly and Company, USA \| \| Elios Therapeutics, USA \| \| EMD Serono Inc., USA \| \| Endo International plc, USA \| \| Endocyte, USA \| \| EnGeneIC, USA \| \| Enzon Pharmaceuticals, USA \| \| EpicentRx, Inc., USA \| \| EpiThany, USA \| \| Epitopoietic Research, USA \| \| Epizyme, USA \| \| Erytech Pharma, USA \| \| Esanex, USA \| \| Esperance Pharmaceuticals, USA \| \| Ethicon Inc., USA \| \| Etubics, USA \| \| Eureka Therapeutics, USA \| \| Exelixis, USA \| \| Exicure, USA \| \| Fate Therapeutics, USA \| \| FibroGen, USA \| \| Five Prime Therapeutics, USA \| \| FLX Bio, USA \| \| Foresee Pharmaceuticals, USA \| \| Forest Laboratories, USA \| \| FORMA Therapeutics, USA \| \| Formation Biologics, USA \| \| Formula Pharmaceuticals, USA \| \| Fortress Biotech, USA \| \| Forty Seven, USA \| \| Foundation Medicine, USA \| \| FUJIFILM Pharmaceuticals, USA \| \| Fujirebio Diagnostics, Inc., USA \| \| G1 Therapeutics, USA \| \| Galectin Therapeutics, USA \| \| Galera Therapeutics, USA \| \| Genelux Corporation, USA \| \| Genentech, USA \| \| General Electric, USA \| \| Genewiz, USA \| \| Genomic Health Inc., USA \| \| Genoptix, USA \| \| Genprex, USA \| \| Gen-Probe , USA \| \| Genus Oncology, USA \| \| Gilead Sciences Inc., USA \| \| GLG Pharma, USA \| \| Gliknik, USA \| \| GlycoMimetics, USA \| \| Gradalis, USA \| \| GT Biopharma, USA \| \| GTx, USA \| \| Guardant Health, USA \| \| H3 Biomedicine, USA \| \| Halozyme Therapeutics, USA \| \| Heat Biologics, USA \| \| HedgePath Pharmaceuticals, USA \| \| Hemispherx Biopharma, USA \| \| Heron Therapeutics, USA \| \| Histogenetics, USA \| \| Hologic Inc., USA \| \| Horizon Pharma, USA \| \| Hospira, USA \| \| HTG Molecular Diagnostics, Inc., USA \| \| HUYA Bioscience, USA \| \| IBM, USA \| \| Idera Pharmaceuticals, USA \| \| IGF Oncology, USA \| \| Ignyta, Inc., USA \| \| Illumina, USA \| \| Imago BioSciences, USA \| \| ImClone Systems, USA \| \| Immatics US, USA \| \| Immix Biopharma, USA \| \| Immune Design, USA \| \| ImmunoCellular Therapeutics, USA \| \| ImmunoGen Inc., USA \| \| Immunomedics Inc., USA \| \| ImmunoMet, USA \| \| Immunomic Therapeutics, USA \| \| ImmunoRestoration, USA \| \| Immunotope, USA \| \| Incyte Corporation, USA \| \| Infinity Pharmaceuticals Inc., USA \| \| Innocrin Pharmaceuticals, Inc., USA \| \| Inovio Pharmaceuticals, Inc., USA \| \| Intensity Therapeutics, USA \| \| Intezyne, USA \| \| Intrexon, USA \| \| Intuitive Surgical Inc., USA \| \| Invitae, USA \| \| Io Therapeutics, USA \| \| Ionis Pharmaceuticals, USA \| \| Iovance Biotherapeutics, USA \| \| IRX Therapeutics, USA \| \| Janssen Biotech Inc., USA \| \| Jina Pharmaceuticals, USA \| \| Johnson and Johnson, USA \| \| Jounce Therapeutics, USA \| \| Kadmon Pharmaceuticals, USA \| \| Kaleido Biosciences, USA \| \| Karyopharm Therapeutics, USA \| \| Keystone Nano, USA \| \| Kiromic, USA \| \| Kite Pharma, USA \| \| Kura Oncology, USA \| \| LAM Therapeutics, USA \| \| Leadiant Biosciences, USA \| \| Leap Therapeutics, USA \| \| Lilly Oncology, USA \| \| Lixte Biotechnology, USA \| \| Loxo Oncology, USA \| \| LSK Biopharma, USA \| \| Lycera, USA \| \| MabVax Therapeutics, USA \| \| MacroGenics, USA \| \| Madison Vaccines, USA \| \| Madrigal Pharmaceuticals, USA \| \| Mateon Therapeutics, USA \| \| Medicenna Therapeutics, USA \| \| MedImmune LLC, USA \| \| Medivation, USA \| \| MEI Pharma, USA \| \| Merck and Co. - MSD, USA \| \| Merrimack Pharmaceuticals, USA \| \| Mersana Therapeutics, USA \| \| Meso Scale Diagnostics LLC, USA \| \| MetronomX, USA \| \| Midatech Pharma US, USA \| \| MimiVax, USA \| \| Minneamrita Therapeutics, USA \| \| miRagen Therapeutics, USA \| \| Mirati Therapeutics Inc., USA \| \| Moderna Therapeutics, USA \| \| Molecular Templates, USA \| \| Moleculin Biotech, USA \| \| Monopar Therapeutics, USA \| \| Morphotek Inc., USA \| \| MultiVir, USA \| \| Mundipharma International Ltd., USA \| \| Mustang Bio, USA \| \| Myovant Sciences, USA \| \| Myriad Genetics, USA \| \| NanOlogy, USA \| \| NanoString Technologies, USA \| \| NantBioScience, USA \| \| NantKwest, USA \| \| NantPharma, USA \| \| Nascent Biotech, USA \| \| Natera Inc., USA \| \| Nektar Therapeutics, USA \| \| Neon Therapeutics, USA \| \| NEONC Technologies, USA \| \| Neovia Oncology, USA \| \| Neumedicines, USA \| \| New Approaches to Neuroblastoma, USA \| \| NewLink Genetics Corporation, USA \| \| NMT Pharmaceuticals, USA \| \| Northwest Biotherapeutics, USA \| \| Novita Pharmaceuticals, USA \| \| Novonco Therapeutics, USA \| \| NS Pharma, USA \| \| NuCana, USA \| \| NuvOx Pharma, USA \| \| Nymox Pharmaceutical, USA \| \| Oblato (GtreeBNT), USA \| \| Odonate Therapeutics, USA \| \| OncBioMune Pharmaceuticals, USA \| \| Oncoceutics, USA \| \| OncoGenex Pharmaceuticals, Inc., USA \| \| OncoImmune Inc., USA \| \| Oncolix, USA \| \| Oncologie, USA \| \| OncoMed Pharmaceuticals, USA \| \| Onconova Therapeutics, Inc., USA \| \| OncoPep, USA \| \| OncoSec Medical, USA \| \| Oncotelic, USA \| \| Oncovir, USA \| \| Oncternal Therapeutics, USA \| \| Onyx Pharmaceuticals, Inc., USA \| \| Optum Healthcare Solutions, USA \| \| Orbis Biosciences, USA \| \| Orbus Therapeutics, USA \| \| ORIC Pharmaceuticals, USA \| \| OSI Pharmaceuticals, Inc., USA \| \| Osiris Therapeutics, Inc., USA \| \| Oxford Immunotec Global, USA \| \| Panacea Pharmaceuticals, USA \| \| PellePharm, USA \| \| Pellficure Pharmaceuticals, USA \| \| Peloton Therapeutics, Inc., USA \| \| PerkinElmer, USA \| \| Pfizer, USA \| \| Pharmacyclics, USA \| \| PharmaCyte Biotech, USA \| \| Pharmion, USA \| \| Philip Morris International, USA \| \| Phoenix Biotechnology, USA \| \| Phosplatin Therapeutics, USA \| \| Pieris Pharmaceuticals, USA \| \| PIN Pharma, USA \| \| Pinnacle Biologics, USA \| \| Pique Therapeutics, USA \| \| Placon Therapeutics, USA \| \| Plexxikon, USA \| \| Polaris Pharmaceuticals, USA \| \| Polynoma, USA \| \| Portola Pharmaceuticals, USA \| \| Poseida Therapeutics, USA \| \| Precision Biologics, USA \| \| Principia Biopharma, USA \| \| Progenics Pharmaceuticals, USA \| \| Promedior, Inc., USA \| \| Prothena Corp, USA \| \| Provectus Biopharmaceuticals Inc., USA \| \| PsiOxus Therapeutics, USA \| \| PTC Therapeutics, USA \| \| Pulse Biosciences, USA \| \| Puma Biotechnology, USA \| \| Purdue Pharma, USA \| \| QED Therapeutics, USA \| \| Radio Medix, USA \| \| Radius Health, USA \| \| Rafael Pharmaceuticals, USA \| \| Reata Pharmaceuticals, Inc., USA \| \| RedHill Biopharma, USA \| \| Regeneron Pharmaceuticals, USA \| \| Replimmune, USA \| \| Research Institute, USA \| \| Rexahn Pharmaceuticals, USA \| \| Rgenix, USA \| \| Rich Pharmaceuticals, USA \| \| Samumed, USA \| \| Samus Therapeutics, USA \| \| Sanofi Genzyme, USA \| \| Seattle Genetics, USA \| \| Selecta Biosciences, USA \| \| SELLAS Life Sciences Group, Inc., USA \| \| Sequenom Laboratories, USA \| \| Sequenta Inc., USA \| \| Sermonix Pharmaceuticals, USA \| \| Shuttle Pharmaceuticals, USA \| \| SignalRx Pharmaceuticals, USA \| \| Soligenix, Inc., USA \| \| SonaCare Medical, LLC, USA \| \| Sophiris Bio, USA \| \| Soricimed Biopharma, USA \| \| Sorrento Therapeutics, USA \| \| Spectrum Pharmaceuticals, USA \| \| Splash Pharmaceuticals, USA \| \| SpringWorks Therapeutics, USA \| \| StemImmune, USA \| \| Stemline Therapeutics, USA \| \| StemMed, USA \| \| Sun BioPharma, USA \| \| Sunesis Pharmaceuticals, USA \| \| Sunovion Pharmaceuticals, USA \| \| Surface Oncology, USA \| \| Sutro Biopharma, USA \| \| Syndax Pharmaceuticals, USA \| \| SynDevRx, USA \| \| SynerGene Therapeutics, USA \| \| Synta Pharmaceuticals Corp., USA \| \| Synthon Pharmaceuticals, USA \| \| Syros Pharmaceuticals, USA \| \| Takara Bio USA, USA \| \| Takeda Pharmaceutical - Millennium Pharmaceuticals (Takeda Oncology), USA \| \| Tanvex BioPharma, USA \| \| TapImmune, USA \| \| Tara Immuno-Oncology, USA \| \| Targeted Diagnostics and Therapeutics, Inc., USA \| \| TARIS Biomedical, USA \| \| Tarix Orphan, USA \| \| Tarveda Therapeutics, USA \| \| Telomere Diagnostics, Inc., USA \| \| Terumo BCT, USA \| \| Tesaro, Inc., USA \| \| TG Therapeutics, Inc., USA \| \| Therakos, Inc., USA \| \| Therapy Consortium, USA \| \| Threshold Pharmaceuticals, USA \| \| Tocagen, USA \| \| Tokai Pharmaceuticals, Inc., USA \| \| Tolero Pharmaceuticals, USA \| \| TP Therapeutics, USA \| \| TRACON Pharmaceuticals, USA \| \| Tragara Pharmaceuticals, USA \| \| Transgene, USA \| \| Treos Bio, USA \| \| TrovaGene, USA \| \| TTC Oncology, USA \| \| TVAX Biomedical, USA \| \| Tyme Technologies, USA \| \| Tyrogenex, USA \| \| UbiVac, USA \| \| United Therapeutics, USA \| \| Unum Therapeutics, USA \| \| Upsher-Smith, USA \| \| UroGen Pharma, USA \| \| Vaccinex, USA \| \| Vaccinogen, USA \| \| Vanquish Oncology, USA \| \| Varian Medical Systems, USA \| \| VasGene Therapeutics, USA \| \| VBI Vaccines, USA \| \| Veana Therapeutics, USA \| \| Ventana Medical Systems, USA \| \| Veracyte, Inc., USA \| \| Verastem Oncology, USA \| \| Veridex LLC, USA \| \| Vertex Pharmaceuticals, USA \| \| Vicus Therapeutics, USA \| \| ViewRay, Inc., USA \| \| Vigeo Therapeutics, USA \| \| Viracta Therapeutics, USA \| \| ViroPharma, USA \| \| Vyriad, USA \| \| Wyeth Pharmaceuticals, USA \| \| X4 Pharmaceuticals, USA \| \| XBiotech, USA \| \| Xcovery, USA \| \| Xcovery LLC, USA \| \| XEME Biopharma, USA \| \| Xencor, USA \| \| Xenetic Biosciences, USA \| \| Xynomic Pharmaceuticals, USA \| \| Y-mAbs Therapeutics, USA \| \| Zenith Epigenetics, USA \| \| ZIOPHARM Oncology, Inc., USA  **Academic institutions, Research facilities/networks**   \| University of Buenos Aires, Argentina \| \| --- \| \| ANZAC Research Institute, Australia \| \| Asbestos Diseases Research Institute, Australia \| \| Australian National University, Australia \| \| Burnet Institute, Australia \| \| Cabrini Institute, Australia \| \| Calvary Mater Newcastle, Australia \| \| Cancer Therapeutics CRC, Australia \| \| Centenary Institute, Australia \| \| Centre for Children's Health Research, Australia \| \| Children’s’ Medical Research Institute, Australia \| \| Children's Cancer Institute, Australia \| \| Curtin University, Australia \| \| Deakin University, Australia \| \| Edith Cowan University, Australia \| \| Epworth Research Institute, Australia \| \| Flinders University, Australia \| \| Garvan Institute of Medical Research, Australia \| \| Griffith University, Australia \| \| Hunter Medical Research Institute, Australia \| \| Kolling Institute of Medical Research, Australia \| \| La Trobe University, Australia \| \| Macquarie University, Australia \| \| Melanoma Institute Australia, Australia \| \| Melbourne Melanoma Project, Australia \| \| Menzies Institute for Medical Research, Australia \| \| Menzies School of Health Research, Australia \| \| Monash University, Australia \| \| Murdoch Childrens Research Institute, Australia \| \| Primary Care Collaborative Cancer Clinical Trials Group, Australia \| \| Psycho-oncology Co-operative Research Group, Australia \| \| Queensland University of Technology, Australia \| \| Royal Alexandra Hospital for Children, Australia \| \| Royal Brisbane and Women's Hospital, Australia \| \| Sir Charles Gairdner Hospital, Australia \| \| South Australian Health and Medical Research Institute, Australia \| \| St John of God Subiaco Hospital, Australia \| \| St Vincent's Hospital Melbourne, Australia \| \| St. Vincent's Institute of Medical Research, Australia \| \| Sydney Catalyst, Australia \| \| The Atlantic Philanthropies - Queensland Head and Neck Cancer Centre, Australia \| \| The Harry Perkins Institute of Medical Research, Australia \| \| The John Curtin School of Medical Research, Australia \| \| The Kinghorn Cancer Centre, Australia \| \| The Lowitja Institute - Cooperative Research Centre for Aboriginal and Torres Strait Islander Health, Australia \| \| The University of Auckland, Australia \| \| The University of Newcastle, Australia \| \| The University of Queensland, Australia \| \| University Innovative Research Team, Australia \| \| University of Melbourne, Australia \| \| University of New South Wales - UNSW, Australia \| \| University of South Australia, Australia \| \| University of Southern Queensland, Australia \| \| University of Sydney, Australia \| \| University of Tasmania, Australia \| \| University of Western Australia, Australia \| \| University of Wollongong, Australia \| \| Victorian Breast Cancer Research Consortium, Australia \| \| Victorian Comprehensive Cancer Centre, Australia \| \| Walter and Eliza Hall Institute of Medical Research, Australia \| \| Medical University of Graz, Austria \| \| Medical University of Vienna, Austria \| \| Oncotyrol - Center for Personalized Cancer Medicine, Austria \| \| Paracelsus Medical University, Austria \| \| University of Innsbruck, Austria \| \| Southeast University, Bangladesh \| \| Centre Anticancereux Près De L'Université De Liège, Belgium \| \| Ghent University, Belgium \| \| Ghent University Hospital, Belgium \| \| KU Leuven, Belgium \| \| Université libre de Bruxelles, Belgium \| \| University of Antwerp, Belgium \| \| University of Abomey-Calavi - Faculty of Health Sciences, Benin \| \| Antonio Prudente Foundation - AC Camargo / CIPE, Brazil \| \| Barretos Cancer Hospital, Brazil \| \| Clinical Hospital of Porto Alegre, Brazil \| \| Federal University of Rio de Janeiro, Brazil \| \| Nove de Julho University, Brazil \| \| Beatrice Hunter Cancer Research Institute, Canada \| \| Centre hospitalier de l'Université de Montréal, Canada \| \| Concordia University, Canada \| \| Garron Family Foundation - Garron Family Cancer Centre, Canada \| \| Goodman Cancer Research Centre, Canada \| \| Hamilton Health Sciences, Canada \| \| Institut de recherche Robert-Sauvé en santé et en sécurité du travail - IRSST, Canada \| \| Institut du cancer de Montreal, Canada \| \| Institute for Clinical Evaluative Sciences, Canada \| \| Kidney Cancer Research Network of Canada, Canada \| \| Lawson Health Research Institute, Canada \| \| London Regional Cancer Program, Canada \| \| McGill University, Canada \| \| McGill University Health Centre, Canada \| \| Ministry of Research, Innovation and Science of Canada - Ontario Institute for Cancer Research, Canada \| \| Mount Sinai Hospital, Canada \| \| National Institute of Scientific Research - INRS, Canada \| \| Ottawa Hospital Research Institute, Canada \| \| Princess Margaret Cancer Centre, Canada \| \| Saskatchewan Cancer Agency, Canada \| \| Sick Kids Hospital, Canada \| \| Terry Fox Foundation - Terry Fox Research Institute, Canada \| \| The Campbell Family Cancer Research Institute, Canada \| \| The Cedars Cancer Institute at the McGill University Health Centre, Canada \| \| Université Laval - Cancer Research Center, Canada \| \| University Health Network - UHN, Canada \| \| University of British Columbia, Canada \| \| University of Calgary, Canada \| \| University of Manitoba, Canada \| \| University of Ottawa, Canada \| \| University of Toronto, Canada \| \| Vancouver Coastal Health Research Institute, Canada \| \| Women and Children's Health Research Institute, Canada \| \| Andrés Bello National University, Chile \| \| Anhui Medical University, China \| \| Army Medical University, China \| \| Beijing Institute For Brain Disorders, China \| \| Beijing Tongren Hospital , China \| \| Beijing University of Chemical Technology, China \| \| Bengbu Medical College, China \| \| Binzhou Medical College, China \| \| Capital Medical University Beijing, China \| \| Central South University, China \| \| Changhai Hospital, China \| \| Children's Hospital of Chongqing Medical University, China \| \| China Medical University, China \| \| China Pharmaceutical University, China \| \| Chinese University of Hong-Kong, China \| \| Chongqing Medical University, China \| \| Dalian Medical University, China \| \| Fudan University, China \| \| Fudan University Shanghai Cancer Center, China \| \| Fujian Medical University, China \| \| Fuzhou General Hospital, China \| \| Guangdong Medical College, China \| \| Guangxi Medical University, China \| \| Guangxi University of Science and Technology, China \| \| Guangzhou Medical University, China \| \| Hainan Cancer Hospital, China \| \| Harbin Medical University, China \| \| Harbin Medical University Cancer Hospital, China \| \| Hong-Kong Baptist University, China \| \| Hong-Kong Polytechnic University, China \| \| Huashan Hospital Affiliated to Fudan University, China \| \| Huazhong Agricultural University, China \| \| Huazhong University of Science and Technology, China \| \| Hubei University of Medicine, China \| \| Hunan Normal University, China \| \| Institut Pasteur of Shanghai, China \| \| Institute of Materia Medica, China \| \| Jiangsu University, China \| \| Jiangxi Cancer Hospital, China \| \| Jilin University, China \| \| Jinan University, China \| \| Jining Medical University, China \| \| Kunming Medical University, China \| \| Kunming University of Science and Technology, China \| \| Lanzhou University Second Hospital, China \| \| Liaoning Medical University, China \| \| Liaoning Provincial People's Hospital, China \| \| Linyi People's Hospital, China \| \| Luzhou Medical College, China \| \| Nanchang University, China \| \| Nanfang Hospital, China \| \| Nanjing Medical University, China \| \| Nantong University, China \| \| Ningbo University, China \| \| Ningbo University Medical School, China \| \| Ningxia Medical University, China \| \| North China University of Science and Technology, China \| \| Northeast Agricultural University, China \| \| Parkway Health, China \| \| Peking Union Medical College, China \| \| Peking University, China \| \| Peking University Cancer Hospital and Institute, China \| \| Peking University First Hospital, China \| \| Peking University People's Hospital, China \| \| Renji Hospital, China \| \| Ruijin Hospital, China \| \| Second Military Medical University, China \| \| Shandong Provincial Collaborative Innovation Center for Neurodegenerative Disorders, China \| \| Shandong University, China \| \| Shanghai Cancer Institute, China \| \| Shanghai Changzheng Hospital, China \| \| Shanghai Chest Hospital, China \| \| Shanghai Engineering Research Center, China \| \| Shanghai Jiao Tong University, China \| \| Shanghai Municipal Hospital of Traditional Chinese Medicine, China \| \| Shanghai Shenkang Hospital Development Center, China \| \| Shanghai Sixth People's Hospital, China \| \| Shanghai University of Traditional Chinese Medicine, China \| \| Shantou University Medical College, China \| \| Shanxi Medical University, China \| \| Sheng Jing Hospital, China \| \| Shenyang Pharmaceutical University, China \| \| Shihezi University, China \| \| Sichuan Agricultural University, China \| \| Sichuan University, China \| \| Soochow University, China \| \| Southern Medical University, China \| \| Southern Medical University Shenzhen Hengsheng Hospital, China \| \| Sun Yat-sen Memorial Hospital, China \| \| Sun Yat-sen University Cancer Center, China \| \| The First Affiliated Hospital of Guangzhou Medical University, China \| \| The First Affiliated Hospital of Henan University of Science and Technology, China \| \| The First Affiliated Hospital of Sun Yat-sen University, China \| \| The First Affiliated Hospital of Wenzhou Medical University, China \| \| The First Affiliated Hospital of Xiamen University, China \| \| The First Affiliated Hospital of Xi'an Jiaotong University, China \| \| The First Affiliated Hospital of Zhengzhou University, China \| \| The First Hospital of Jilin University, China \| \| The First People's Hospital of Kunshan, China \| \| The General Hospital of the Peoples Liberation Army - 301 Hospital, China \| \| The Second Affiliated Hospital of Harbin Medical University, China \| \| The Second Affiliated Hospital of Nanchang University, China \| \| The Second Affiliated Hospital of Shanghai Jiao Tong University, China \| \| The Second Affiliated Hospital of Soochow University, China \| \| The Second Hospital of Shandong University, China \| \| The Third Xiangya Hospital of Central South University, China \| \| The University of Hong-Kong, China \| \| Third Affiliated Hospital of Harbin Medical University, China \| \| Third Xiangya Hospital of Central South University, China \| \| Tianjin Medical University, China \| \| Tianjin Medical University Cancer Institute and Hospital, China \| \| Tianjin Medical University General Hospital, China \| \| Tochigi Cancer Center, China \| \| Tongji Medical College, China \| \| Tongji University, China \| \| Tsinghua University, China \| \| University of Hong-Kong Faculty of Medicine, China \| \| University of Macau, China \| \| University of South China, China \| \| Weifang Medical University, China \| \| Wenzhou Medical University, China \| \| Wuhan University of Science and Technology, China \| \| Xinhua Hospital, China \| \| Xinhua Translational Institute for Cancer Pain, China \| \| Xinjiang Medical University, China \| \| Xinxiang Medical University, China \| \| Yantai Yuhuangding Hospital, China \| \| Yunnan Institute of Digestive Disease, China \| \| Zhejiang Cancer Hospital, China \| \| Zhejiang Sci-Tech University, China \| \| Zhejiang University, China \| \| Zhengzhou University, China \| \| Zhongnan Hospital of Wuhan University, China \| \| Zhongshan Hospital, China \| \| Instituto de Cancerología SA, Colombia \| \| University of Valle, Colombia \| \| Cyprus Institute of Neurology And Genetics, Cyprus \| \| Central European Institute of Technology, Czech Republic \| \| Charles University in Prague, Czech Republic \| \| Masaryk University, Czech Republic \| \| Motol University Hospital, Czech Republic \| \| Palacký University, Czech Republic \| \| Regional Centre for Applied Molecular Oncology - RECAMO, Czech Republic \| \| St. Anne's University Hospital Brno, Czech Republic \| \| Aarhus University, Denmark \| \| Aarhus University Hospital, Denmark \| \| Copenhagen University Hospital, Denmark \| \| Danish Center For Interventional Research In Radiation Oncology - CIRRO, Denmark \| \| Danish Centre For Translational Breast Cancer Research - DCTB, Denmark \| \| Danish Lymphoma Group, Denmark \| \| Herlev Hospital, Denmark \| \| Odense University Hospital, Denmark \| \| Rigshospitalet, Denmark \| \| Sino-Danish Breast Cancer Research Centre, Denmark \| \| University of Copenhagen, Denmark \| \| University of Southern Denmark, Denmark \| \| Vejle Hospital, Denmark \| \| Alexandria University - Medical Research Institute, Egypt \| \| Fakkous Center for Cancer and Allied Diseases, Egypt \| \| Academy of Finland - Centre of Excellence in Translational Cancer Biology, Finland \| \| Biocenter Finland, Finland \| \| Biocentrum Helsinki, Finland \| \| Kuopio University Hospital, Finland \| \| National Graduate School of Clinical Investigation - VKTK, Finland \| \| Oulu University Hospital, Finland \| \| Tampere University Hospital, Finland \| \| Turku Graduate School of Biomedical Sciences, Finland \| \| Turku University Hospital, Finland \| \| University of Eastern Finland, Finland \| \| University of Helsinki, Finland \| \| University of Oulu, Finland \| \| Aix Marseille University, France \| \| Assistance Publique Hopitaux De Paris - AP-HP, France \| \| Centre Antoine Lacassagne, France \| \| Centre Eugène Marquis, France \| \| Centre Georges-François Leclerc, France \| \| Centre Henri Becquerel, France \| \| Centre Léon Bérard, France \| \| Centre Oscar Lambret, France \| \| Centre Paul Strauss, France \| \| Centre Régional François Baclesse, France \| \| Centre Régional Jean Perrin, France \| \| Grenoble Alpes University, France \| \| Hospices Civils de Lyon, France \| \| Institut Bergonié, France \| \| Institut Carnot CALYM, France \| \| Institut de Cancérologie de Lorraine, France \| \| Institut de Cancérologie de l'Ouest, France \| \| Institut du Cancer de Montpellier, France \| \| Institut Gustave Roussy, France \| \| Institut Jean Godinot, France \| \| Institut National du Cancer - SItes de Recherche Intégrée sur le Cancer - SIRIC, France \| \| Institut Paoli-Calmettes, France \| \| Institut Pasteur, France \| \| Institut Universitaire du Cancer Toulouse Oncopole, France \| \| Nantes University Hospital, France \| \| Paris 13 University, France \| \| Paris Descartes University, France \| \| Paris Diderot University, France \| \| Pierre-and-Marie-Curie University, France \| \| UNICANCER, France \| \| University of Lyon - Claude Bernard University Lyon 1, France \| \| University of Montpellier, France \| \| University of Nice Sophia Antipolis, France \| \| University of Paris-Sud, France \| \| University of Reims Champagne-Ardenne, France \| \| University of Rennes 1, France \| \| University of Strasbourg, France \| \| National Cancer Center of Georgia, Georgia \| \| Berlin Institute of Health, Germany \| \| Center for Integrated Protein Science Munich - CIPSM, Germany \| \| Charité University Medicine Berlin, Germany \| \| Christian-Albrechts-University Kiel, Germany \| \| Düsseldorf University, Germany \| \| Elite Network of Bavaria, Germany \| \| Functional Genomics Center Zurich, Germany \| \| Georg-Speyer-Haus Foundation, Germany \| \| German Cancer Consortium - DKTK, Germany \| \| German Cancer Research Center - DKFZ, Germany \| \| German Center for Lung Research - DZL, Germany \| \| Hannover Medical School, Germany \| \| Heidelberg University, Germany \| \| Heidelberg University Hospital, Germany \| \| Hospital of the University of Munich, Germany \| \| Institute for Prevention and Occupational Medicine - German Social Accident Insurance, Germany \| \| Johanniter-Krankenhaus Bonn, Germany \| \| LOEWE Centre for Cell and Gene Therapy, Germany \| \| Ludwig Maximilians University of Munich, Germany \| \| National Center for Tumor Diseases - NCT, Germany \| \| Ruhr University Bochum, Germany \| \| Saarland University, Germany \| \| Sino-German Center for Research Promotion, Germany \| \| St Anna Children's Cancer Research Institute, Germany \| \| Technical University of Munich, Germany \| \| University Hospital Bonn, Germany \| \| University Hospital of Giessen and Marburg, Germany \| \| University Medical Center of Johannes Gutenberg University Mainz, Germany \| \| University of Bonn - School of Medicine, Germany \| \| University of Cologne - Faculty of Medicine, Germany \| \| University of Cologne Center for Molecular Medicine, Germany \| \| University of Erlangen-Nuremberg, Germany \| \| University of Marburg, Germany \| \| University of Tübingen, Germany \| \| University of Ulm, Germany \| \| University of Würzburg, Germany \| \| International Institute of Anticancer Research, Greece \| \| Hungarian Brain Research Program, Hungary \| \| University of Pécs Medical School, Hungary \| \| University of Rijeka, Hungary \| \| Landspitali University Hospital, Iceland \| \| Apollo Cancer Institutes of Hyderabad, India \| \| Cancer Institute - WIA, India \| \| Delhi State Cancer Institute, India \| \| Dharamshila Cancer Hospital and Research Centre, India \| \| Dr Bhubaneswar Borooah Cancer Institute, India \| \| Gujarat Cancer and Research Institute, India \| \| Indonesian Center for Expertise in Retinoblastoma - ICER, India \| \| Kidwai Memorial Institute of Oncology, India \| \| Manipal Academy of Higher Education, India \| \| National Institute of Cancer Prevention and Research - NICPR, India \| \| Ruby Hall Clinic, India \| \| Sher-I-Kashmir Institute of Medical Sciences - SKIMS, India \| \| Tata Memorial Centre, India \| \| University of Delhi, India \| \| Rumah Sakit Kanker Dharmais - National Cancer Center, Indonesia \| \| Ahvaz Jundishapur University of Medical Sciences, Iran \| \| Avicenna Research Institute, Iran \| \| Ferdowsi University of Mashhad, Iran \| \| Golestan University of Medical Sciences, Iran \| \| Hamedan University of Medical Sciences, Iran \| \| Imam Khomeini Medical Center, Iran \| \| Iran University of Medical Sciences, Iran \| \| Isfahan University of Medical Sciences, Iran \| \| Kashan University of Medical Sciences, Iran \| \| Kurdistan University of Medical Sciences, Iran \| \| MAHAK Pediatric Cancer Treatment and Research Center, Iran \| \| Mashhad University of Medical Sciences, Iran \| \| Mazandaran University of Medical Sciences, Iran \| \| National Institute of Genetic Engineering and Biotechnology - NIGEB, Iran \| \| Pasteur Institute of Iran, Iran \| \| Sabzevar University of Medical Sciences, Iran \| \| Shahid Beheshti University of Medical Sciences and Health Services, Iran \| \| Shiraz Institute for Cancer Research, Iran \| \| Shiraz University of Medical Sciences, Iran \| \| Stem Cell Technology Research Center, Iran \| \| Tabriz University of Medical Sciences, Iran \| \| Tarbiat Modares University, Iran \| \| Tehran University of Medical Sciences, Iran \| \| University of Isfahan, Iran \| \| University of Tehran, Iran \| \| Urmia University, Iran \| \| Zahedan University of Medical Sciences, Iran \| \| Queen's University, Ireland \| \| UPMC Whitfield Cancer Centre, Ireland \| \| Ariel University, Israel \| \| Tel Aviv University, Israel \| \| Varda and Boaz Dotan Research Center in Hemato-Oncology, Israel \| \| Ghislieri College, Italy \| \| Interuniversity Consortium for the National Bio-Oncology - CINBO, Italy \| \| IRCCS Santa Lucia Foundation, Italy \| \| Marche Polytechnic University, Italy \| \| National Cancer Institute Regina Elena, Italy \| \| National Tumor Institute - IRCCS Foundation, Italy \| \| Reference Center for Oncology - Centro di Riferimento Oncologico, Italy \| \| Reference Centre for Epidemiology and Cancer Prevention in Piemonte - CPO, Italy \| \| Sapienza University of Rome, Italy \| \| The University of Milan, Italy \| \| Tuscan Tumor Institute - ITT, Italy \| \| University of Bologna, Italy \| \| University of Brescia, Italy \| \| University of Ferrara, Italy \| \| University of Florence, Italy \| \| University of Genoa, Italy \| \| University of Insubria, Italy \| \| University of Padua, Italy \| \| University of Palermo, Italy \| \| University of Pisa, Italy \| \| University of Salerno, Italy \| \| University of Turin, Italy \| \| University of Verona, Italy \| \| Aichi Cancer Center Research Institute, Japan \| \| Cancer Research Institute of Kanazawa University, Japan \| \| Chiba Cancer Center, Japan \| \| Fujita Health University, Japan \| \| Fukuoka University, Japan \| \| Fukushima Medical University, Japan \| \| Gunma University, Japan \| \| Hirosaki University, Japan \| \| Hiroshima University, Japan \| \| Hokkaido University Graduate School of Medicine, Japan \| \| Hyogo College of Medicine, Japan \| \| Japan Clinical Cancer Research Organization, Japan \| \| Japanese Foundation for Cancer Research, Japan \| \| Jichi Medical University, Japan \| \| Jikei University, Japan \| \| Kanagawa Cancer Center, Japan \| \| Kansai Medical University, Japan \| \| Kawasaki Medical School, Japan \| \| Keio University, Japan \| \| Kindai University, Japan \| \| Kochi University, Japan \| \| Kumamoto University, Japan \| \| Miyagi Cancer Center, Japan \| \| Nagasaki University, Japan \| \| Nagoya City University, Japan \| \| Nakayama Cancer Research Institute, Japan \| \| Nara Medical University, Japan \| \| National Cancer Center Hospital East, Japan \| \| National Cancer Center Japan, Japan \| \| National Center for Child Health and Development, Japan \| \| National Center for Global Health and Medicine, Japan \| \| National Institute of Biomedical Innovation - NIBIO, Japan \| \| National Institute of Radiological Sciences - NIRS, Japan \| \| Niigata Cancer Center, Japan \| \| Okayama University, Japan \| \| Okayama University Hospital, Japan \| \| Osaka Dental University, Japan \| \| Osaka Medical Center for Cancer and Cardiovascular Disease, Japan \| \| Osaka University, Japan \| \| Research Institute for Diseases of Old Age, Japan \| \| RIKEN, Japan \| \| Saitama Cancer Center, Japan \| \| Sendai Kousei Hospital, Japan \| \| Shizuoka Cancer Center, Japan \| \| The Radiation Effects Research Foundation - RERF, Japan \| \| Tokai University, Japan \| \| Tokyo Medical and Dental University, Japan \| \| Tokyo Medical University, Japan \| \| Tokyo Medical University Cancer Center, Japan \| \| Toranomon Hospital, Japan \| \| University of Occupational and Environmental Health - UOEH, Japan \| \| Jordan University of Science and Technology, Jordan \| \| King Hussein Cancer Foundation - King Hussein Cancer Center, Jordan \| \| University of Jordan, Jordan \| \| Kazakh Research Institute of Oncology and Radiology, Kazakhstan \| \| Kuwait University, Kuwait \| \| August Kirchenstein Institute of Microbiology and Virology, Latvia \| \| Lebanese University, Lebanon \| \| Naef K. Basile Cancer Institute, Lebanon \| \| Luxembourg Institute of Health, Luxembourg \| \| International Medical University, Malaysia \| \| Malaysian University of Technology, Malaysia \| \| National University of Malaysia, Malaysia \| \| Sains Malaysia University, Malaysia \| \| University of Malaya, Malaysia \| \| University Putra Malaysia, Malaysia \| \| Centro de Investigación de Cancer en Sonora - CICS, Mexico \| \| Children's Hospital Federico Gómez, Mexico \| \| National Autonomous University of Mexico - UNAM, Mexico \| \| Centre Scientifique de Monaco, Monaco \| \| Université Mohammed VI des sciences de la santé, Morocco \| \| Nepal Cancer Hospital and Research Center, Nepal \| \| Cancer Genomics Center, Netherlands \| \| Dutch Colorectal Cancer Group, Netherlands \| \| Erasmus University Medical Center, Netherlands \| \| Flanders Institute for Biotechnology - VIB, Netherlands \| \| Leiden University Medical Center, Netherlands \| \| Netherlands Cancer Institute - NKI, Netherlands \| \| Netherlands Comprehensive Cancer Organisation - IKNL, Netherlands \| \| Radboud University Medical Center, Netherlands \| \| Top Institute Food and Nutrition - TiFN, Netherlands \| \| University Medical Center Utrecht, Netherlands \| \| University of Amsterdam, Netherlands \| \| Vanderbilt University Medical Center - VUMC, Netherlands \| \| Maurice Wilkins Centre, New Zealand \| \| University of Otago, New Zealand \| \| Victoria University of Wellington, New Zealand \| \| University of Abuja, Nigeria \| \| Akershus University Hospital, Norway \| \| Cancer Registry of Norway, Norway \| \| Fonna Hospital Trust, Norway \| \| Haukeland University Hospital, Norway \| \| Helsinki University Central Hospital, Norway \| \| Norwegian University of Science and Technology, Norway \| \| Oslo University Hospital, Norway \| \| The Arctic University of Norway - University of Tromsø, Norway \| \| University of Bergen, Norway \| \| University of Oslo, Norway \| \| Aga Khan University Hospital, Pakistan \| \| Papua New Guinea National Cancer Centre, Papua New Guinea \| \| National University of Asuncion, Paraguay \| \| Gdańsk Medical University, Poland \| \| Greater Poland Cancer Center, Poland \| \| International Hereditary Cancer Center, Poland \| \| Jagiellonian University Medical College, Poland \| \| Maria Skłodowska-Curie Institute of Oncology, Poland \| \| Medical University of Bialystok, Poland \| \| Medical University of Lodz, Poland \| \| Medical University of Lublin, Poland \| \| Medical University of Silesia, Poland \| \| Medical University of Warsaw, Poland \| \| Pomeranian Medical University in Szczecin, Poland \| \| Poznan University of Medical Sciences, Poland \| \| University of Lodz, Poland \| \| University of Rzeszów, Poland \| \| Wrocław Medical University, Poland \| \| Portuguese Institute of Oncology - Francisco Gentil Institute, Portugal \| \| University of Porto, Portugal \| \| University of Medicine and Pharmacy of Tîrgu Mureş, Romania \| \| Victor Babes University of Medicine and Pharmacy, Romania \| \| N.N. Blokhin Russian Cancer Research Center, Russia \| \| Tomsk State University, Russia \| \| Imam Abdulrahman Bin Faisal University, Saudi Arabia \| \| King Abdulaziz University, Saudi Arabia \| \| King Abdullah Medical City - Oncology Center, Saudi Arabia \| \| King Abdullah University of Science and Technology, Saudi Arabia \| \| King Khalid University, Saudi Arabia \| \| King Saud University, Saudi Arabia \| \| Sheikh Mohammed Hussien Al-Amoudi Center of Excellence in Breast Cancer, Saudi Arabia \| \| Cancer Science Institute of Singapore, Singapore \| \| Nanyang Technological University, Singapore \| \| National Cancer Centre Singapore, Singapore \| \| National University Cancer Institute of Singapore, Singapore \| \| National University of Singapore, Singapore \| \| St Luke's Medical Centre, Singapore \| \| Ljubljana Institute of Oncology, Slovenia \| \| Cape Peninsula University of Technology, Faculty of Health and Wellness Sciences, South Africa \| \| Rhodes University, South Africa \| \| University of Cape Town, South Africa \| \| Ajou University, South Korea \| \| Asan Institute For Life Science, South Korea \| \| Catholic Kwandong University International St Mary's Hospital, South Korea \| \| Catholic University of Korea, South Korea \| \| Chonbuk National University, South Korea \| \| Chonbuk National University Hospital, South Korea \| \| Chonnam National University, South Korea \| \| Chonnam National University Hwasun Hospital, South Korea \| \| Chosun University, South Korea \| \| Chung Ang University, South Korea \| \| Chungbuk National University, South Korea \| \| Chungnam National University, South Korea \| \| Dankook University, South Korea \| \| Dong-A University, South Korea \| \| Ewha Womans University, South Korea \| \| Gachon University Gil Medical Center, South Korea \| \| Hallym University, South Korea \| \| Hanyang University, South Korea \| \| Inha University, South Korea \| \| Inje University, South Korea \| \| Innovative Research Institute for Cell Therapy, South Korea \| \| Institute for Basic Science, South Korea \| \| Jeju National University Hospital, South Korea \| \| Kangbuk Samsung Hospital, South Korea \| \| Kangwon National University, South Korea \| \| Keimyung University, South Korea \| \| Konkuk University, South Korea \| \| Korea Basic Science Institute, South Korea \| \| Korea Food Research Institute, South Korea \| \| Korea Institute of Oriental Medicine - KIOM, South Korea \| \| Korea Institute of Radiological and Medical Sciences - KIRAMS, South Korea \| \| Korea Institute of Science and Technology - KIST, South Korea \| \| Korea Research Institute of Bioscience and Biotechnology - KRIBB, South Korea \| \| Korea Research Institute of Chemical Technology, South Korea \| \| Korea University, South Korea \| \| Korean Cancer Study Group, South Korea \| \| Kosin University, South Korea \| \| Kwangwoon University, South Korea \| \| Kyung Hee University, South Korea \| \| Kyungpook National University, South Korea \| \| Kyungpook National University Hospital, South Korea \| \| National Cancer Center Korea, South Korea \| \| Pusan ​​National University Hospital, South Korea \| \| Pusan National University, South Korea \| \| Samsung Medical Center, South Korea \| \| Seoul National University, South Korea \| \| Seoul National University Bundang Hospital, South Korea \| \| Seoul National University Hospital, South Korea \| \| Sookmyung Women's University, South Korea \| \| Soonchunhyang University, South Korea \| \| Wonkwang University, South Korea \| \| Yeungnam University, South Korea \| \| Yonsei Cancer Center, South Korea \| \| Yonsei University, South Korea \| \| Catalan Institute of Oncology - ICO, Spain \| \| CIBER Bioengineering, Biomaterials and Nanomedicine, Spain \| \| CIBER Epidemiology And Public Health, Spain \| \| CIBER Hepatic And Digestive Diseases, Spain \| \| CIBER Physiopathology of Obesity and Nutrition, Spain \| \| Hospital Del Mar Research Institute IMIM, Spain \| \| Spanish Biomedical Research Centre in Cancer - CIBERONC, Spain \| \| University of Cordoba, Spain \| \| University of Girona, Spain \| \| University of Navarra - Center for Applied Medical Research - CIMA, Spain \| \| University of Oviedo, Spain \| \| University of the Basque Country, Spain \| \| Khartoum Oncology Specialized Center, Sudan \| \| Chalmers University of Technology, Sweden \| \| Create Health, Sweden \| \| Jönköping University, Sweden \| \| Karolinska Institute, Sweden \| \| Karolinska Institute - Breast cancer theme center, Sweden \| \| Karolinska Institute - Center for Immune Modulatory Therapies for Autoimmunity and Cancer, Sweden \| \| Linköping University Hospital, Sweden \| \| Lund University, Sweden \| \| Malmö University Hospital, Sweden \| \| Örebro University, Sweden \| \| Sahlgrenska University Hospital, Sweden \| \| Skåne University Hospital, Sweden \| \| Swedish e-Science Research Centre, Sweden \| \| Umeå University, Sweden \| \| University of Gothenburg, Sweden \| \| Uppsala University, Sweden \| \| Uppsala University Hospital, Sweden \| \| Balgrist University Hospital, Switzerland \| \| ETH Zurich, Switzerland \| \| Geneva University Hospital, Switzerland \| \| University of Basel, Switzerland \| \| University of Zurich, Switzerland \| \| Academia Sinica, Taiwan \| \| Academia Sinica - Institute of Biomedical Sciences, Taiwan \| \| Asia University, Taiwan \| \| Cardinal Tien Hospital, Taiwan \| \| Cathay General Hospital, Taiwan \| \| Chang Gung Memorial Hospital, Taiwan \| \| Chang Gung University, Taiwan \| \| Changhua Christian Hospital, Taiwan \| \| Cheng Hsin General Hospital, Taiwan \| \| Chi Mei Medical Center, Taiwan \| \| China Medical University Taiwan, Taiwan \| \| Chung Shan Medical University, Taiwan \| \| E-Da Hospital, Taiwan \| \| Far Eastern Memorial Hospital, Taiwan \| \| Fu Jen Catholic University, Taiwan \| \| International Center of Excellence in Cancer Research, Taiwan \| \| Kaohsiung Chang Gung Memorial Hospital, Taiwan \| \| Kaohsiung Datong Hospital, Taiwan \| \| Kaohsiung Medical University, Taiwan \| \| Kaohsiung Veterans General Hospital, Taiwan \| \| Mackay Memorial Hospital, Taiwan \| \| National Center of Excellence for Clinical Trials and Research of Taiwan, Taiwan \| \| National Cheng Kung University, Taiwan \| \| National Cheng Kung University Hospital, Taiwan \| \| National Chiao Tung University, Taiwan \| \| National Defense Medical Center, Taiwan \| \| National Sun Yat-sen University, Taiwan \| \| National Taiwan University, Taiwan \| \| National Taiwan University Hospital, Taiwan \| \| National Tsing Hua University, Taiwan \| \| National Yang Ming University Hospital, Taiwan \| \| National Yang-Ming University, Taiwan \| \| Shin Kong Wu Ho-Su Memorial Hospital, Taiwan \| \| Show Chwan Memorial Hospital, Taiwan \| \| Taichung Armed Forces General Hospital, Taiwan \| \| Taichung Veterans General Hospital, Taiwan \| \| Taipei City Hospital, Taiwan \| \| Taipei Medical University, Taiwan \| \| Taipei Medical University Hospital, Taiwan \| \| Taipei Veterans General Hospital, Taiwan \| \| Tri-Service General Hospital, Taiwan \| \| Tzu Chi General Hospital, Taiwan \| \| Wanfang Hospital, Taiwan \| \| Ocean Road Cancer Institute, Tanzania \| \| Chiang Mai University, Thailand \| \| Chulabhorn Research Institute, Thailand \| \| Chulalongkorn University, Thailand \| \| Khon Kaen University, Thailand \| \| King Chulalongkorn Memorial Hospital, Thailand \| \| Mahidol University, Thailand \| \| Prince of Songkla University, Thailand \| \| Ege University, Turkey \| \| Hacettepe University, Turkey \| \| Istanbul University, Turkey \| \| United Arab Emirates University, United Arab Emirates \| \| Cancer Research UK Imperial Centre, United Kingdom \| \| Cardiff University, United Kingdom \| \| Experimental Cancer Medicine Centres - ECMC, United Kingdom \| \| Francis Crick Institute, United Kingdom \| \| Guy's and St Thomas' NHS Foundation Trust, United Kingdom \| \| Imperial College London, United Kingdom \| \| King's College Hospital, United Kingdom \| \| King's College London, United Kingdom \| \| King's Health Partners Comprehensive Cancer Centre, United Kingdom \| \| Life Sciences Research Network Wales, United Kingdom \| \| London School of Hygiene and Tropical Medicine, United Kingdom \| \| Manchester Cancer Research Centre, United Kingdom \| \| Newcastle University, United Kingdom \| \| Newcastle University - Northern Institute for Cancer Research, United Kingdom \| \| Princess Alexandra Hospital NHS Trust, United Kingdom \| \| The Institute of Cancer Research - ICR, United Kingdom \| \| The National Cancer Research Institute UK - NCRI, United Kingdom \| \| The Royal Marsden NHS Foundation Trust, United Kingdom \| \| UCL Hospitals Charitable Foundation, United Kingdom \| \| UK National Institute for Health Research - Biomedical Research Centre at Guy's and St Thomas' NHS Foundation Trust and King College London, United Kingdom \| \| UK National Institute for Health Research - Cambridge Biomedical Research Centre, United Kingdom \| \| UK National Institute for Health Research - Great Ormond Street Hospital Biomedical Research Centre, United Kingdom \| \| UK National Institute for Health Research - Imperial Biomedical Research Centre, United Kingdom \| \| UK National Institute for Health Research - Manchester Biomedical Research Centre, United Kingdom \| \| UK National Institute for Health Research - Oxford Biomedical Research Centre, United Kingdom \| \| UK National Institute for Health Research - The Royal Marsden Biomedical Research Centre, United Kingdom \| \| UK National Institute for Health Research - UCLH Biomedical Research Centre, United Kingdom \| \| University College London - UCL, United Kingdom \| \| University of Aberdeen, United Kingdom \| \| University of Birmingham, United Kingdom \| \| University of Bristol, United Kingdom \| \| University of Cambridge, United Kingdom \| \| University of Edinburgh, United Kingdom \| \| University of Glasgow, United Kingdom \| \| University of Leeds, United Kingdom \| \| University of Manchester, United Kingdom \| \| University of Nottingham, United Kingdom \| \| University of Oxford, United Kingdom \| \| University of Salford, United Kingdom \| \| University of Sheffield, United Kingdom \| \| University of Southampton, United Kingdom \| \| Velindre NHS Trust, United Kingdom \| \| Wales Gene Park, United Kingdom \| \| Agilent Technologies Mass Spectrometry Center of Excellence, USA \| \| Albert Einstein Cancer Center, USA \| \| Albert Einstein College of Medicine, USA \| \| Alvin J. Siteman Cancer Center, USA \| \| Arkansas Biosciences Institute, USA \| \| Augusta University, USA \| \| Basser Center for BRCA, USA \| \| Baylor College of Medicine, USA \| \| Beth Israel Deaconess Medical Center, USA \| \| Boston University, USA \| \| Brigham And Women's Hospital, USA \| \| Burzynski Research Institute, USA \| \| Caltech Ucla Joint Center For Translational Medicine, USA \| \| Cancer Institute of New Jersey, USA \| \| Cancer Prevention And Research Institute of Texas, USA \| \| Cancer Prevention Institute of California, USA \| \| Cancer Treatment Centers of America, USA \| \| Cancer Vaccine Institute, USA \| \| Carole and Ray Neag Comprehensive Cancer Center, USA \| \| Case Comprehensive Cancer Center, USA \| \| Case Western Reserve University, USA \| \| Case Western Reserve University - Clinical and Translational Science Collaborative, USA \| \| Center For Inherited Disease Research, USA \| \| Center for Innovations in Quality, Effectiveness and Safety, USA \| \| Chicago Biomedical Consortium, USA \| \| Children's Healthcare of Atlanta, USA \| \| Children's Hospital Los Angeles, USA \| \| Children's Hospital of Wisconsin Research Institute, USA \| \| Cincinnati Cancer Center, USA \| \| City of Hope National Medical Center, USA \| \| Cleveland Clinic, USA \| \| Clinical And Translational Science Center, USA \| \| Colon Cancer Family Registry, USA \| \| Columbia University, USA \| \| Comprehensive Cancer Center - Wake Forest Baptist Health, USA \| \| Dan L. Duncan Comprehensive Cancer Center, USA \| \| Dana-Farber Cancer Institute, USA \| \| Dartmouth-Hitchcock's Norris Cotton Cancer Center, USA \| \| David Geffen School of Medicine At UCLA, USA \| \| David H. Koch Charitable Foundation - Koch Institute for Integrative Cancer Research at MIT, USA \| \| David M. Rubenstein Foundation - David M. Rubenstein Center for Pancreatic Cancer Research, USA \| \| DeBartolo Family Foundation - DeBartolo Family Personalized Medicine Institute, USA \| \| Des Moines University, USA \| \| Dialysis Clinic, Inc., USA \| \| Duke Cancer Institute, USA \| \| Duke University, USA \| \| Eastern Cooperative Oncology Group, USA \| \| Emory University, USA \| \| Feist-Weiller Cancer Center, USA \| \| Florida State University, USA \| \| Forbes Institute for Cancer Discovery, USA \| \| Fox Chase Cancer Center , USA \| \| Fred and Pamela Buffett Cancer Center, USA \| \| Fred Hutchinson Cancer Research Center, USA \| \| Geisinger Commonwealth School of Medicine, USA \| \| Geoffrey Beene Cancer Research Center, USA \| \| George Mason University, USA \| \| Georgetown Lombardi Comprehensive Cancer Center, USA \| \| Georgetown University, USA \| \| H. Lee Moffitt Cancer Center and Research Institute, USA \| \| Harvard Medical School, USA \| \| Harvard Stem Cell Institute, USA \| \| Harvard University, USA \| \| Henry Ford Health System, USA \| \| Huntsman Cancer Institute, USA \| \| Icahn School of Medicine at Mount Sinai, USA \| \| Indiana Clinical and Translational Sciences Institute, USA \| \| Indiana University, USA \| \| Indiana University Melvin and Bren Simon Cancer Center, USA \| \| Jess and Mildred Fisher Center for Hereditary Cancer and Clinical Genomics Research, USA \| \| Johns Hopkins University, USA \| \| Kent State University, USA \| \| Kentucky Lung Cancer Research Program, USA \| \| Louisiana Cancer Research Center, USA \| \| Louisiana State University Health Sciences Center New Orleans, USA \| \| Magee-Womens Research Institute, USA \| \| Marshfield Clinic Research Institute, USA \| \| Massachusetts General Hospital, USA \| \| Massachusetts General Hospital Cancer Center, USA \| \| Mayo Clinic, USA \| \| MD Anderson Cancer Center Sheikh Khalifa Bin Zayed Al Nahyan Institute for Personalized Cancer Therapy, USA \| \| Medical College of Wisconsin, USA \| \| Medical College of Wisconsin Cancer Center, USA \| \| Medical University of South Carolina, USA \| \| Medical University of South Carolina Hollings Cancer Center, USA \| \| Memorial Sloan Kettering Cancer Center, USA \| \| Murtha Cancer Center - Walter Reed National Military Medical Center, USA \| \| Nationwide Children's Hospital, USA \| \| Nevada Cancer Institute, USA \| \| New Mexico Tumor Registry, USA \| \| Norma J. Vinger Center for Breast Care, USA \| \| Northwestern University, USA \| \| Nurses' Health Studies, USA \| \| NYU Langone Health - Laura and Isaac Perlmutter Cancer Center, USA \| \| Ohio State University, USA \| \| Ohio State University - Mathematical Biosciences Institute, USA \| \| Ohio State University - The James Cancer Hospital, USA \| \| Ohio State University Wexner Medical Center, USA \| \| Oklahoma Medical Research Foundation, USA \| \| Oregon Clinical and Translational Research Institute, USA \| \| Ovarian Cancer Institute, USA \| \| Pacific Health Research and Education Institute, USA \| \| Patient-Centered Outcomes Research Institute - PCORI, USA \| \| Penn State Cancer Institute, USA \| \| Pharmacogenomics Research Network, USA \| \| Providence John Wayne Cancer Institute, USA \| \| Purdue University Center for Cancer Research, USA \| \| Radiation Therapy Oncology Group, USA \| \| Robert H. Lurie Comprehensive Cancer Center, USA \| \| Roswell Park Comprehensive Cancer Center, USA \| \| RTI International, USA \| \| Rutgers University, USA \| \| Rutgers University - Rutgers Cancer Institute of New Jersey, USA \| \| Saint Louis University Cancer Center, USA \| \| Sbarro Health Research Organization - SHRO, USA \| \| Seattle Children's Hospital, USA \| \| Shriners Hospitals For Children, USA \| \| Sol Goldman Charitable Trust - Sol Goldman Pancreatic Cancer Research Center, USA \| \| St. John's University, USA \| \| St. Jude Children's Research Hospital, USA \| \| St. Luke's Radiation Oncology Network, USA \| \| Stanford University, USA \| \| Stanford University - Stanford Cancer Institute, USA \| \| Stony Brook Cancer Center, USA \| \| Sylvester Comprehensive Cancer Center, USA \| \| Temple University, USA \| \| The Bloomberg-Kimmel Institute for Cancer Immunotherapy, USA \| \| The Delaware CTR ACCEL program, USA \| \| The Duncan Family Institute for Cancer Prevention and Risk Assessment, USA \| \| The Eshelman Foundation - The Eshelman Institute for Innovation, USA \| \| The George Washington University Cancer Institute, USA \| \| The Harvard Clinical and Translational Science Center, USA \| \| The McCombs Foundation - Red and Charline McCombs Institute for the Early Detection and Treatment of Cancer, USA \| \| The Rebecca Susan Buffett Foundation - Fred and Pamela Buffett Cancer Center, USA \| \| The Research Institute at Nationwide Children’s Hospital, USA \| \| The Rockefeller University, USA \| \| The Skip Viragh Foundation - Skip Viragh Center for Pancreas Cancer Clinical Research and Patient Care, USA \| \| The Starr Foundation - Starr Cancer Consortium, USA \| \| The University of Adelaide, USA \| \| The University of Alabama at Birmingham, USA \| \| The University of Alabama at Birmingham Comprehensive Cancer Center, USA \| \| The University of Arizona Cancer Center, USA \| \| The University of Chicago, USA \| \| The University of Kansas Cancer Center, USA \| \| The University of Kansas Medical Center, USA \| \| The University of North Carolina at Chapel Hill, USA \| \| The University of North Carolina at Chapel Hill - Lineberger Comprehensive Cancer Center, USA \| \| The University of Oklahoma - Stephenson Cancer Center, USA \| \| The University of Texas at Austin, USA \| \| Thomas Jefferson University, USA \| \| Tobacco Settlement Edowment Trust - Oklahoma Center for Adult Stem Cell Research, USA \| \| Tulane University, USA \| \| Tulane University - Tulane Cancer Center, USA \| \| UCSF Helen Diller Family Comprehensive Cancer Center, USA \| \| Uniformed Services University, USA \| \| University of Alabama, USA \| \| University of California, USA \| \| University of California Davis, USA \| \| University of California Irvine - UCI, USA \| \| University of California Los Angeles - AIDS Institute, USA \| \| University of California Los Angeles - Jonsson Comprehensive Cancer Center, USA \| \| University of California Los Angeles - UCLA, USA \| \| University of California San Diego - UCSD, USA \| \| University of California San Francisco - UCSF, USA \| \| University of Chicago Comprehensive Cancer Center, USA \| \| University of Cincinnati, USA \| \| University of Colorado, USA \| \| University of Colorado Cancer Center, USA \| \| University of Florida, USA \| \| University of Florida James Graham Brown Cancer Center, USA \| \| University of Hawaii Cancer Center, USA \| \| University of Houston, USA \| \| University of Illinois at Urbana–Champaign, USA \| \| University of Illinois Cancer Center, USA \| \| University of Iowa, USA \| \| University of Iowa Hospitals and Clinics - Holden Comprehensive Cancer Center, USA \| \| University of Johannesburg, USA \| \| University of Kentucky, USA \| \| University of Kentucky - Markey Cancer Center, USA \| \| University of Liverpool, USA \| \| University of Louisville School of Medicine, USA \| \| University of Maryland, USA \| \| University of Maryland - Marlene and Stewart Greenebaum Comprehensive Cancer Center, USA \| \| University of Massachusetts Center for Clinical and Translational Science, USA \| \| University of Miami, USA \| \| University of Michigan, USA \| \| University of Michigan Comprehensive Cancer Center, USA \| \| University of Minnesota - Masonic Cancer Center, USA \| \| University of Minnesota Twin Cities, USA \| \| University of Mississippi Medical Center, USA \| \| University of Missouri, USA \| \| University of Nebraska Medical Center, USA \| \| University of Nebraska–Lincoln, USA \| \| University of New Mexico, USA \| \| University of New Mexico Comprehensive Cancer Center, USA \| \| University of Notre Dame, USA \| \| University of Oklahoma Health Sciences Center, USA \| \| University of Pennsylvania, USA \| \| University of Pennsylvania - Center of Excellence in Envirnomental Toxicology, USA \| \| University of Pennsylvania Perelman School of Medicine, USA \| \| University of Pennsylvania Perelman School of Medicine - Center for Molecular Studies in Digestive and Liver Diseases, USA \| \| University of Pittsburgh, USA \| \| University of Pittsburgh Cancer Institute, USA \| \| University of Pittsburgh Medical Center, USA \| \| University of South Alabama - Mitchell Cancer Institute, USA \| \| University of South Carolina, USA \| \| University of South Florida, USA \| \| University of Southern California, USA \| \| University of Texas - MD Anderson Cancer Center, USA \| \| University of Texas Health - Science Center at Houston, USA \| \| University of Texas Southwestern Medical Center, USA \| \| University of Utah, USA \| \| University of Virginia Cancer Center, USA \| \| University of Washington, USA \| \| University of Wisconsin Carbone Cancer Center, USA \| \| University of Wisconsin-Madison, USA \| \| US Military Cancer Institute, USA \| \| Vanderbilt University, USA \| \| Vanderbilt-Ingram Cancer Center, USA \| \| Vattikuti Urology Institute, USA \| \| Vincent Memorial Hospital, USA \| \| Virginia Commonwealth University - Massey Cancer Center, USA \| \| Wake Forest University, USA \| \| Washington University in St. Louis, USA \| \| Washington University in St. Louis - Institute of Clinical and Translational Sciences, USA \| \| Wayne State University, USA \| \| Wayne State University - Barbara Ann Karmanos Cancer Institute, USA \| \| West Cancer Center, USA \| \| West Virginia University, USA \| \| West Virginia University Cancer Institute, USA \| \| Winship Cancer Institute, USA \| \| Yale University, USA \| \| Yale University - Yale Cancer Center, USA \| \| Cancer Diseases Hospital, Zambia \| \| |

**Appendix B.**

**List of the 45 selected cancer centers and institutions drawn from http://www.cancerindex.org.**

| Aarhus University Hospital - Department of Oncology, Denmark |
| --- |
| Aichi Cancer Center , Japan |
| Blokhin Russian Cancer Research Centre, Russian Federation |
| Buenos Aires Cancer Center - Centro Oncológico Buenos Aires, Argentina |
| Cancer Care Ontario, Canada |
| Cancer Institute and Hospital, Chinese Academy of Medical Sciences, China |
| Cancer Research Institute CRI, USA |
| Catalan Institute of Oncology, Spain |
| Centre Léon Bérard, France |
| Comprehensive Cancer Centre the Netherlands, The Netherlands |
| Dana-Farber Cancer Institute, USA |
| Deutsches Krebforschungszentrum DKFZ, Germany |
| Dokuz Eylül University, Institute of Oncology, Turkey |
| Duke Cancer Institute, USA |
| Erasmus MC Daniel den Hoed Cancer Center, The Netherlands |
| European Institute of Oncology, Italy |
| Fondazione IRCCS Istituto Nazionale dei Tumori, Italy |
| Fred Hutchinson Cancer Research Center, USA |
| Institut Gustave Roussy, France |
| Istituto Tumori Bari, Italy |
| King Hussein Cancer Center, Saudi Arabia |
| Ljubljana Institute of Oncology, Poland |
| Ludwig Cancer Research, USA |
| Mayo Clinic Cancer Center, USA |
| MD Anderson Cancer Center, USA |
| Memorial Sloan-Kettering Cancer Center, USA |
| Moffitt Cancer Center, USA |
| National University Cancer Institute, Singapore, Singapore |
| Netherlands Cancer Institute - Antoni van Leeuwenhoek Hospital, The Netherlands |
| NKI Amsterdam, The Netherlands |
| NYU Cancer Institute, USA |
| Princess Margaret Cancer Centre, Canada |
| Purdue University Center for Cancer Research, India |
| Stanford Cancer Institute, USA |
| The Institute of Cancer Research, UK |
| The Ohio State University Comprehensive Cancer Center, USA |
| University Hospital Linköping - Regional Cancer Center SouthEast, Sweden |
| University Hospital of Umeå - Regional Cancer Center North, Sweden |
| University of Chicago Comprehensive Cancer Center, USA |
| University of Hawaii Cancer Center, USA |
| University of Pittsburgh Cancer Institute, USA |
| Uppsala University Hospital - Regional Cancer Centre Uppsala-Örebro, Sweden |
| Vancouver Cancer Centre, Canada  Yale Cancer Center, USA |
