# Supplementary material for: Mapping the Global Cancer Research Funding Landscape
Source: JNCI Cancer Spectr. 2019 Oct 7;3(4):pkz069. doi: 10.1093/jncics/pkz069 (PMC7049992; doi:10.1093/jncics/pkz069)
Supplement: pkz069_Supplementary_Data [file pkz069_supplementary_data.zip › SCHMUTZ_Cancer_Research_Funding_Mapping_Manuscript_Revised.docx]

**Mapping of the Global Cancer Research Funding Landscape**

**Anna Schmutz^1,^^[[1]](#footnote-2)^*; Claire Salignat^1^; Daria Plotkina^1^, Amandine Devouassoux^1^, Teresa Lee^1^;  Melina Arnold^1^ ; Morten Ervik^1^; Olaf Kelm^1^**

1. *International Agency for Research on Cancer, 150 cours Albert Thomas, 69008 Lyon, France, +33 6 11 20 31 87*

**Keywords**: [CANCER](http://apps.webofknowledge.com/OneClickSearch.do?product=WOS&search_mode=OneClickSearch&excludeEventConfig=ExcludeIfFromFullRecPage&colName=WOS&SID=E1fnyiMpPF1VTKCKoGm&field=TS&value=CANCER&uncondQuotes=true); [RESEARCH](http://apps.webofknowledge.com/OneClickSearch.do?product=WOS&search_mode=OneClickSearch&excludeEventConfig=ExcludeIfFromFullRecPage&colName=WOS&SID=E1fnyiMpPF1VTKCKoGm&field=TS&value=RESEARCH&uncondQuotes=true); [FUNDING](http://apps.webofknowledge.com/OneClickSearch.do?product=WOS&search_mode=OneClickSearch&excludeEventConfig=ExcludeIfFromFullRecPage&colName=WOS&SID=E1fnyiMpPF1VTKCKoGm&field=TS&value=FUNDING&uncondQuotes=true); [BIBLIOMETRIC APPROACH](http://apps.webofknowledge.com/OneClickSearch.do?product=WOS&search_mode=OneClickSearch&excludeEventConfig=ExcludeIfFromFullRecPage&colName=WOS&SID=E1fnyiMpPF1VTKCKoGm&field=TS&value=BIBLIOMETRIC+APPROACH&uncondQuotes=true); [BOTTOM-UP APPROACH](http://apps.webofknowledge.com/OneClickSearch.do?product=WOS&search_mode=OneClickSearch&excludeEventConfig=ExcludeIfFromFullRecPage&colName=WOS&SID=E1fnyiMpPF1VTKCKoGm&field=TS&value=TOP-DOWN+APPROACH&uncondQuotes=true)

**Abstract**

**PURPOSE:** Global investment in research on non-communicable diseases (NCDs) is on the rise. Cancer as primus inter pares draws particular interest from a wide spectrum of research funders. Next to the private, governmental and academic sectors, philanthropy has carved out a sizeable area in the funding landscape over the last 25 years.

Previous reports describing cancer research funding have looked at the volume of investment in cancer research, but have paid little attention to building strategic intelligence on funders. Moreover, these efforts have focused primarily on well-resourced organizations, neglecting a large number of players with less developed finances.

**METHODS:** In this paper, we combined gnostic data acquisition with agnostic bibliometrics to establish a comprehensive map of the global cancer research funding landscape. The analysis of funding acknowledgments from cancer research papers used in this exercise is a “bottom-up” method which provides a broader perspective on the variety of actors involved. It does not rely on a priori knowledge, nor does it require funders’ support for access to the data.

**RESULTS:** Using this approach, we have identified a total of 4693 organizations from 107 countries engaged in funding cancer research today.

**CONCLUSION:** This is the largest mapping exercise performed to date, and should serve as a knowledge base for future analyses and comparisons aimed at understanding the dynamics and priorities of global cancer research funding.

1. **Introduction**

Non-communicable diseases (NCDs) constitute a major challenge for global health (1; 2) and the global economy (3*;* 4). As part of the United Nations (UN) Sustainable Development Goals, specifically under target 3.4, NCDs have been identified as priority area in need of urgent attention (5). The past years have seen a marked shift in the general perception of this group of diseases, including cardiovascular diseases, cancers, chronic respiratory diseases, and diabetes, being primarily a burden of the rich. However, more recent data clearly show that across this disease spectrum, low socioeconomic status is a major risk factor and NCDs are posing an increasing burden in LMICs (6; 5).

In a recent report, the WHO Independent High-Level Commission on NCDs has laid out six recommendations to move the NCD agenda forward (5). Although research is mentioned only in passing, the recommendation to prioritise and scale up cost-effective, affordable, and evidence-based interventions for NCDs and mental disorders is heavily dependent on past and future research efforts. Hence global efforts to address NCDs not only need to align public health efforts with commensurate funding, they also require targeted, coordinated scientific research, financed at the right level.

Initiatives do exist to coordinate research efforts, such as the National Cancer Policy Board (7) at the national level, as well as the Global Alliance for Chronic Diseases (GACD)^[[2]](#footnote-3)^ and the International Cancer Research Partnership (ICRP)^[[3]](#footnote-4)^ at the international level. The GACD and the ICRP are membership driven efforts that aim to coordinate and prioritise finances in the area of research in NCDs and cancer respectively. Notably, since the early 2000s, the ICRP has devised, refined and promulgated the Common Scientific Outline (CSO), a coding system cataloguing cancer research along a spectrum of six types of research fields. This standard is a powerful means to study priorities of research funding in cancer research, with the potential to allow redirecting of those investments for higher impact.

Clearly membership driven initiatives, while important coordination vehicles, will have an incomplete coverage of the funder landscape. Bottom-up mapping, in contrast, gives a much more reliable picture of all players involved. While a sizeable number of studies have looked at the volume of the investment in cancer research (8; 9; 10), so far no studies have attempted to fully describe all organizations contributing to this investment globally. This study aims to establish a comprehensive database on all cancer research funding entities.

1. **Methodology**

Our investigation, combining established with novel methodological approaches, focused broadly on funding for cancer research, along the axes of support to research projects, research infrastructures and long-term research-based training such as PhD or post-doctoral fellowships. We included all types of research, namely biomedical research, clinical research, population-based research, health services research, and social and behavioral research. On the other hand, funding for advocacy, medical training, outreach activities, and cancer service delivery was excluded.

- 1. **Data extraction**

Since 2013, as part of our core work as the central grants office in the International Agency for Cancer, we have built an initial repository of 480 funding institutions for cancer research by combining incidental discovery based on Google queries with targeted Google news scanning based on 58 funder-specific keywords. This list was complemented by a bibliometric approach, using Web of Science™ (WoS™, Clarivate Analytics) as the reference database. The criteria for inclusion of publications into our analysis were: 1) all publications from specialist journals in the WoS category = “oncology”, 2) all publications containing 13 cancer-specific title keywords^[[4]](#footnote-5)^, and 3) all papers by authors affiliated with 45 selected cancer centers and institutions in the world^[[5]](#footnote-6)^ that were drawn from CancerIndex^[[6]](#footnote-7)^ in such a way as to ensure a balanced geographical representation. We excluded centers focused on treatment, such as “Hospitals”, unless they were clearly indicated as “Research Hospitals”, and using only those centers that have a standardized entry for their organization in WoS. This led us to a geographical coverage of 19 of the 52 countries catalogued in CancerIndex.

Searches based on each of these three criteria were run independently twice, once restricting them to eleven consecutive years from 2008 to 2018 and once setting the parameters to four consecutive years from 2015 to 2018, to ensure inclusion of more recent, and hence less cited funding organizations. The results were pooled into a study set of over 775,000 cancer research papers from 12,000 different journals.

Funding institutions were extracted using the WoS results analysis function from the funding acknowledgments. Not applying any frequency threshold, the first query yielded a crude results table with 100,000 items, the ceiling for WoS data extraction. As the raw data gives a wide array of names for any given individual funding entity, a significant manual investment was required to bring the list down to true unique values. The unrestricted interrogation of the WoS database was clearly unfeasible, and we decided to limit the positive results to those organizations that were either cited as funding sources in at least ten research papers over a period of ten years, or at least in three research papers between 2015 and 2018. On these parameters, the query resulted in 12,872 data points for funding sources that were subsequently manually standardized to remove variants of organizations’ names, bringing the total to 3,514 unique values.

We speculate that funding from private for-profit entities financing cancer research is underrepresented in publications due to intellectual property rights and strategic market positioning, and hence would be less well covered by the bibliometric approach. In order to correct for this, we drew on a report of medicines in development for cancer in 2018 from the Pharmaceutical Research and Manufacturers Association (PhRMA)^[[7]](#footnote-8)^. This publication lists drugs in all phases of development and testing and their corresponding sponsors (11). Data extracted for over 1,600 cancer drugs allowed us to identify 412 new funding organizations from both pharmaceutical and biotechnology industries.

The Union for International Cancer Control (UICC)^[[8]](#footnote-9)^ membership list, which includes 1,071 institutions that aim to reduce the global cancer burden, was used as a fourth source of data. After removing support groups from the list and closely reviewing the remaining organizations’ websites, 200 institutions could be classified as bona fide funding sources.

The fifth source of data to inform our final, comprehensive map, was the list of organizations eligible to receive tax-deductible charitable contributions, available on the US Internal Revenue Service (IRS) website^[[9]](#footnote-10)^. Charitable organizations devoted to cancer research were extracted from more than one million entries, using the same set of cancer-specific keywords as defined for WoS data extraction. 469 additional entities were added to the list.

As a final step the list was completed with an additional 92 funders from the information contained in the annexes of previous surveys on cancer research funding (9; 10).

- 1. **Data classification**

Organizations have been classified according to 18 different types of legal status, and then grouped into 5 broader categories for a simplified overview: governmental organization, international organization, not-for-profit, private sector entity, and research facility.

Institutions and programs that are part of larger legal entities, but that manage their own research programs and are endowed with their own budgets have been treated as separate sources of funding. This includes, for instance, the 13 funding mechanisms of the European Commission involved in cancer research funding. –Regional or municipal governments and their divisions are considered as a single entity, in order to reduce differences between patterns of regional governance.

Although they are not funding sources in the strict sense, research facilities - which include academic institutions, research institutes, hospitals, and research networks - represent 30% of the funding organizations acknowledged in the publications. At first sight one may be tempted to dismiss this category, but on closer inspection, it becomes clear that such institutions do fuel the global research engine through the direct funding that derives from their respective regular budgets. The situation is somewhat more complex than a simple dichotomy between a regular budget funding stream that is entirely absorbed *intra-muros* on the one hand and the *bona fide* funder that distributes all programs funding to the research community. Of course, research facilities themselves are at the receiving end of such external funds, but we would expect acknowledgements to reflect the sources of what effectively would be pass-through funding.

In line with this reasoning, we have disregarded implicit funding references, namely the affiliations of authors, which have been suggested for complete mapping in some publications (12; 13). As the focus of our work was to provide a map of available funding, we surmised that including too many organizations that do not run formal funding programs would distort our results. While one could argue that the very fact that individuals appear on the author list means that they have received or contributed to funding of the published research, we would posit that such contribution would be largely in-kind and anchored. It is precisely the conscious decision to highlight the particular nature of the contribution that for us indicates a player truly contributing to trans-organizational funding.

In order to disambiguate funding sources according to which view a reader might adhere on this controversial aspect of research funding, we ran all analyses once including and once excluding research institutions. The latter represents a very conservative, purist approach to the funding market.

1. **Results and discussion**
   1. **Results**

Using the method described above, we identified 4693 organizations of cancer research funding in the period between 2008 and 2018 (see Appendix A). Almost half of them are not-for-profit while governmental organizations only represent 12% (Fig. 1). Excluding research facilities, not-profit entities represent more than 60% of the total.

Cancer research funders are present in 107 countries (Fig. 2) – 102 if research facilities are excluded (Fig.3) - and 44% of them are located in the USA, compared with 21% in Europe (Fig. 3) and 16% in Asia.

One could hypothesize that the capacity of a country to provide funding for research, and in our case research for cancer, is closely linked to its economic prowess and educational status. We wanted therefore to compare geographically the Human Development Index (HDI) of countries, the Gross Domestic Product (GDP) nominal and the number of funders identified by our method, taking into account the population of a country. An important discrepancy could be indicative of underrepresentation of funders in identified countries.

As can be seen on Fig. 4, our results seem consistent with the different indicators used for quality checking. Only few exceptions are noted, and most countries start to show funder activities as from an HDI above 0.8.

While the objective of this study was to depict the current cancer research funding landscape, including all the entities participating in that effort, these players are not necessarily dedicated to cancer, in that funding is not specifically earmarked - but is used for - cancer research. As a result, 84% of the private for-profit companies have a focus on cancer while only 6% of the public institutions are cancer-specific (in 19 countries out of 107). It is particularly interesting to examine the case of not-for-profit organizations (Fig. 5), as 57% are cancer-specific while 27% represent organizations that fund broader medical research (e.g. brain research, aging process, pediatric research), vulnerable or local communities health, scholarships or research on specific diseases whose understanding can be improved by research on cancer (e.g. Alzheimer’s disease, diabetes).

Finally, we observed that the total number of funding sources has more than doubled since 2008. The number of not-for-profit organizations acknowledged has doubled while the number of private for-profit companies has quadrupled. As can be seen in Fig.6, this has proportionally impacted the number of manuscripts published.

**3.2. Discussion**

This study is the first step in a series of analyses on the global cancer research funding landscape and only examines the numbers of funders without taking into account the size of their funding. Its primary purpose is to list entities funding cancer research and as such, it is not designed to compare countries’ involvement in cancer research.

The examination of the Funding Acknowledgments, which is central to the study, is an indirect way to identify the cancer research funding actors. This approach has equally been used to track research output, manage funding portfolios, and evaluate the impact of grants (14; 15).

However, this exercise has certain limitations and the main issues with the purely bibliometric approach have been highlighted previously (13). Foremost among them is that referencing is based on self-reporting, which although required by funding agencies, is largely unenforced, and on manual data entry, which lists those acknowledged in a non-standardized manner.

As highlighted elsewhere (e.g. 13), the lack of standardization of the funding organizations is a particular bottleneck; some names are generic (e.g. “Ministry of Health”), are mistranslated, or only refer to the institution acronym and cannot be found on the Web. Errors are also found with the example of service providers (e.g. consulting companies) being listed as funding organizations.

In addition 37% of the research papers examined did not cite funding support. Possible explanations include:

- Language issue: WoS™ details Funding Acknowledgments almost exclusively for papers in English and for those in Chinese with English data (16), disregarding other languages, the funding sources that find their impact covered in non-English Journals would not be itemized. For this reason it is impossible to capture the full extent of the private sector activity, especially smaller, local philanthropists that fund researchers publishing in their native languages.

- Cultural differences: Countries with different funding systems might be expected to have markedly different acknowledgment rates. China, for instance, possesses the largest share of publications acknowledging research funding (17).

- Institutional funding: authors’ employers that supported publications are indirectly acknowledged through the authors’ aﬃliations (18),

- Limits of WoS™ precision: Grassano has demonstrated that the the set of funders acknowledged in publications were not correctly listed in about 32% of the cases (18).

These caveats are particularly important when using bibliometric analysis to make comparisons across different countries.

Finally, it is important to mention that the organizations in the IRS list that are used as an additional source of data are all based in the US, which could cause a slight imbalance in the results with an overrepresentation of US charitable organizations.

Since WoS™ began routinely indexing Funding Acknowledgments data only in 2008, our dataset for the part on bibliometrics is limited to the last decade. It could therefore underrepresent some funders that have been more active in supporting research prior to this period and have been less involved of late. However, our combined approach of using bibliometrics as the core approach, supplemented with various other gnostic additions would most likely correct for this factor. Also a period of 11 years is a significant period for funders not to be active, and it is improbable, that funding organizations only active before 2008 would be truly active research supporters today. Furthermore, as there is a significant time lag between the investment and the first outcomes in form of publications, we would estimate the window of active years captured by the bibliometric approach to be larger than 11 years, possibly as much as 15 years.

Many efforts exist to standardize research data, including standardization of research institutions. Our study underlines once again the importance of such efforts, as data scientists work towards integrated metadata on the global research endeavor. As authors before us have done, we would call upon funding organizations and scientists alike to support initiatives such as the Global Research Identifier Database (GRID)^[[10]](#footnote-11)^ by using standardized references. We also hope that WoS™ and others will soon change the entry mode for funding organizations from free text to selection of standardized listing of organizations. This would make mapping exercises such as ours a much easier task in the future.

In summary, despite the data and standardization issues, we present in this paper the largest listing of global cancer research funders to date. We hope it serves as the basis of further efforts to be even more comprehensive.

**Conflict of interest statement**

The authors certify that they have NO affiliations with or involvement in any organization or entity with any financial interest (such as honoraria; educational grants; participation in speakers’ bureaus; membership, employment, consultancies, stock ownership, or other equity interest; and expert testimony or patent-licensing arrangements), or non-financial interest (such as personal or professional relationships, affiliations, knowledge or beliefs) in the subject matter or materials discussed in this manuscript.

**Author contributions**

A.S. and O.K. contributed equally to the design of the study, literature search, data collection, data analysis and production of figures.

C.S., D.P. and A.D. contributed to data collection.

M.E and M.A. designed the maps and scatter plots.

T. L contributed to the initial idea, refined the WoS searches and proofread the manuscript.

A.S. and O.K. wrote and edited the final version of the paper and O.K. had overall responsibility for the direction of the project.

**Acknowledgements**

The work reported in this paper was undertaken during a PhD studentship at the International Agency for Research on Cancer.

The authors are grateful to Lynne Davis from the International Cancer Research Partnership (ICRP) who helped with the provision of some important references.

**Figure Legends**

Figure 1. Pie chart depicting the types of entities identified with the combined bibliometric, gnostic discovery approach. Note the high proportion of not-for-profit organizations.

Figure 2. Geographical representation of the number of funders identified (research facilities included). The majority is located in the USA, followed by Europe, and Asia.

Figure 3. Geographical representation of the number of funders identified (research facilities excluded). Private entities funding cancer research are almost non-existent in low and middle-income countries.

Figure 4. Comparison between HDI, GDP nominal and rate funders/population. Only countries with a small population are standing out.

Figure 5. Funding priorities for not-for-profit organizations.

Figure 6. Number of funders and number of publications between 2008 and 2018.

**References**

(1) World Health Organization. (2018). *Noncommunicable diseases.* Available from: <http://www.who.int/news-room/fact-sheets/detail/noncommunicable-diseases> (accessed: June 28, 2018).

(2) T.A. Ghebreyesus. (2018). Acting on NCDs: counting the cost. Lancet, 391(10134), 1973-1974. doi: https://doi.org/10.1016/S0140-6736(18)30675-5

(3) Pearce A., Sharp L., Hanly P., Barchuk A., Bray F., De Camargo Cancela M., Gupta P., Meheus P., Qiao Y., Sitas F., Wang S., Soerjomataram I. (2018). Productivity losses due to premature mortality from cancer in Brazil, Russia, India, China, and South Africa (BRICS): A population-based comparison. *Cancer Epidemiology*, 16, 27-34. doi: 10.1016/j.canep.2017.12.013

(4) Bloom, D.E., Cafiero, E.T., Jané-Llopis, E., Abrahams-Gessel, S., Bloom, L.R., Fathima, S., Feigl, A.B., Gaziano, T., Mowafi, M., Pandya, A., Prettner, K., Rosenberg, L., Seligman, B., Stein, A.Z., Weinstein, C. (2011). *The Global Economic Burden of Noncommunicable Diseases*. Geneva: World Economic Forum.

(5) Nishtar S., Niinisto S., Sirisena M., *et al.*, Commissioners of the WHO Independent High-Level, Commission on NCDs, (2018). Time to deliver: report of the WHO Independent high-level commission on noncommunicable diseases. *Lancet*, 392(10143), 245-252. doi: <https://doi.org/10.1016/S0140-6736(18)31258-3>

(6) Nugent R., Bertram M., Jan S., Niessen L., Sassi F., Jamison D., González Pier E., Beaglehole R. (2018). Investing in non-communicable disease prevention and management to advance the Sustainable Development Goals. *Lancet.* 391(10134), 2071-2078. doi: 10.1016/S0140-6736(18)30667-6

(7) McGeary M., Burstein M. (1999). *Sources of Cancer Research Funding in the United States.* Prepared for National Cancer Policy Board, Institute of Medicine.

(8) Begum M., Lewison G., Lawler M., Sullivan R. (2018). Mapping the European cancer research landscape. An evidence base for national and Pan-European research and funding. *European Journal of Cancer*; 100, 75-84. doi: 10.1016/j.ejca.2018.04.017

(9) Cancer Australia. (2014). *Cancer Research in Australia: An Overview of Funding to Cancer Research Projects and Research Programs in Australia 2006 to 2011*. Canberra: Cancer Australia.

(10) Eckhouse S., Lewison G., Sullivan R. (2008). Trends in the global funding and activity of cancer research. *Molecular Oncology*, 2(1), 20-32. doi: 10.1016/j.molonc.2008.03.007

(11) Pharmaceutical Research and Manufacturers of America (PhRMA). (2018). *2018 Medicines in Development for Cancer*. Available from: <http://phrma-docs.phrma.org/files/dmfile/2018-Cancer-Drug-List.pdf> (accessed: June 28, 2018).

(12) Sullivan R., Eckhouse S., Lewison G. (2007). Using bibliometrics to inform cancer research policy and spending. *Monitoring financial flows for health research 2007: Behind the Global Numbers*. Geneva: Global Forum for health Research.

(13) Begum M., Lewison G. (2017). Web of Science Research Funding Information: Methodology for its use in Analysis and Evaluation. *Journal of Scientometric Research*, 6(2), 65-73. doi: 10.5530/jscires.6.2.12

(14) Lewison G., Roe P. (2012). The Evaluation of Indian Cancer Research, 1990-2010. *Scientometrics*, 93(1), 167-181. doi: 10.1007/s11192-012-0633-9

(15) Wang J., Shapira P. (2011). Funding acknowledgement analysis: an enhanced tool to investigate research sponsorship impacts: the case of nanotechnology. *Scientometrics*, 87(3), 563-586. doi: 10.1007/s11192-017-2453-4

(16) Tang L., Hu G., Liu W. (2017). Funding acknowledgment analysis: Queries and caveats. *Journal of the Association for Information Science and Technology*, 68(3), 790-794. doi: 10.1002/asi.23713

(17) Costas R., van Leeuwen T. (2012). Approaching the “reward triangle”: General analysis of the presence of funding acknowledgments and “peer interactive communication” in scientific publications. *Journal of the American Society for Information Science and Technology*, 63(8), 1647-1661. doi: https://doi.org/10.1002/asi.22692

(18) Grassano N., Rotolo D., Huttin J, Lang F., Hopkins M. (2016). Funding Data from Publication Acknowledgements: Coverage, Uses and Limitations. *Journal of the Association for Information Science and Technology*, 68(4), 999-1017. doi: 10.1002/jasist.23737

1. * *Corresponding author.*

   *Address:150 cours Albert Thomas, 69008, Lyon*

   *Tel: +33 4 72 73 84 85*

   *Email address:* [schmutza@students.iarc.fr](mailto:schmutza@students.iarc.fr) [↑](#footnote-ref-2)
2. <https://www.gacd.org/> [↑](#footnote-ref-3)
3. <https://www.icrpartnership.org/> [↑](#footnote-ref-4)
4. “Cancer”, “carcinoma”, “chemotherapy”, “glioma”, “immunotherapy”, “lymphoma”, “melanoma”, “metastasis”, “neuroblastoma”, “oncology”, “radiotherapy”, “sarcoma”, and “tumour/tumor”. [↑](#footnote-ref-5)
5. List in Annex B. [↑](#footnote-ref-6)
6. <http://www.cancerindex.org> [↑](#footnote-ref-7)
7. <https://www.phrma.org/> [↑](#footnote-ref-8)
8. <https://www.uicc.org/> [↑](#footnote-ref-9)
9. <https://www.irs.gov/charities-non-profits/tax-exempt-organization-search-bulk-data-downloads> [↑](#footnote-ref-10)
10. https://www.grid.ac/ [↑](#footnote-ref-11)
