# Supplementary figures and images for: Mapping the Global Cancer Research Funding Landscape
Source: JNCI Cancer Spectr. 2019 Oct 7;3(4):pkz069. doi: 10.1093/jncics/pkz069 (PMC7049992; doi:10.1093/jncics/pkz069)

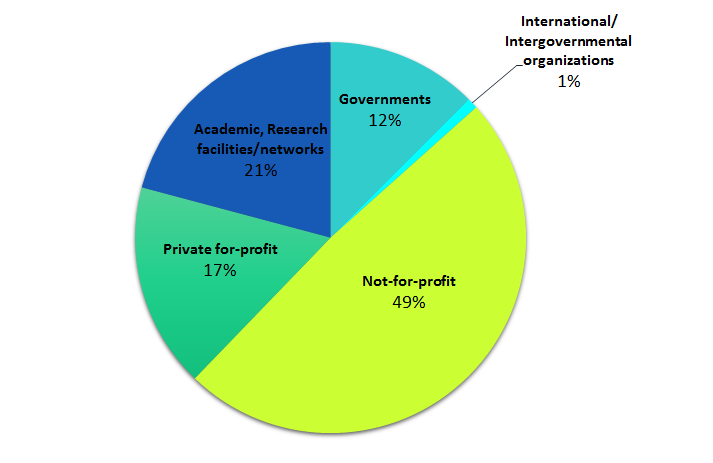

Supplement: pkz069_Supplementary_Data [file pkz069_supplementary_data.zip › SCHMUTZ_Cancer_Research_Funding_Mapping_Figure1.docx]

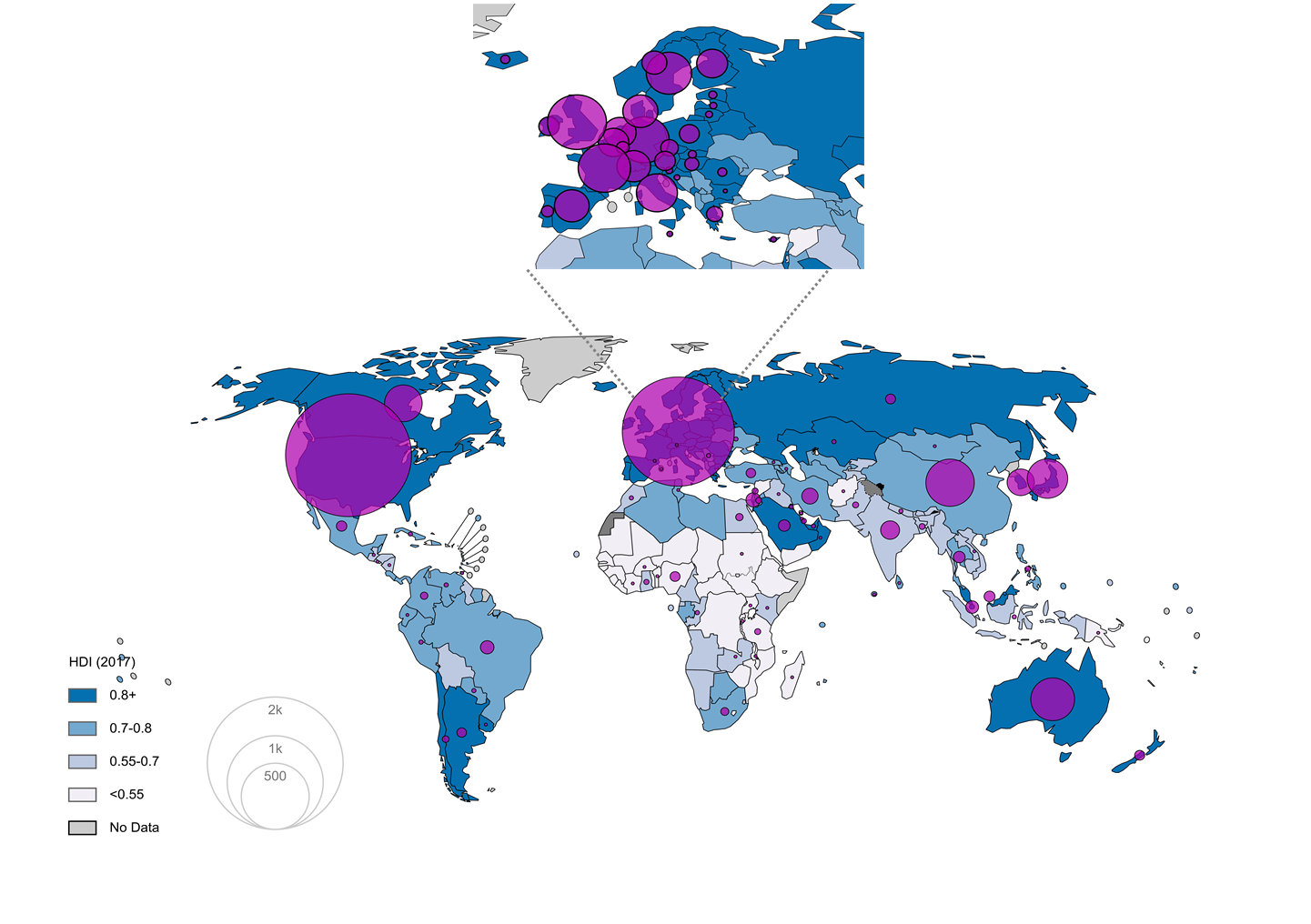

Supplement: pkz069_Supplementary_Data [file pkz069_supplementary_data.zip › SCHMUTZ_Cancer_Research_Funding_Mapping_Figure2.docx]

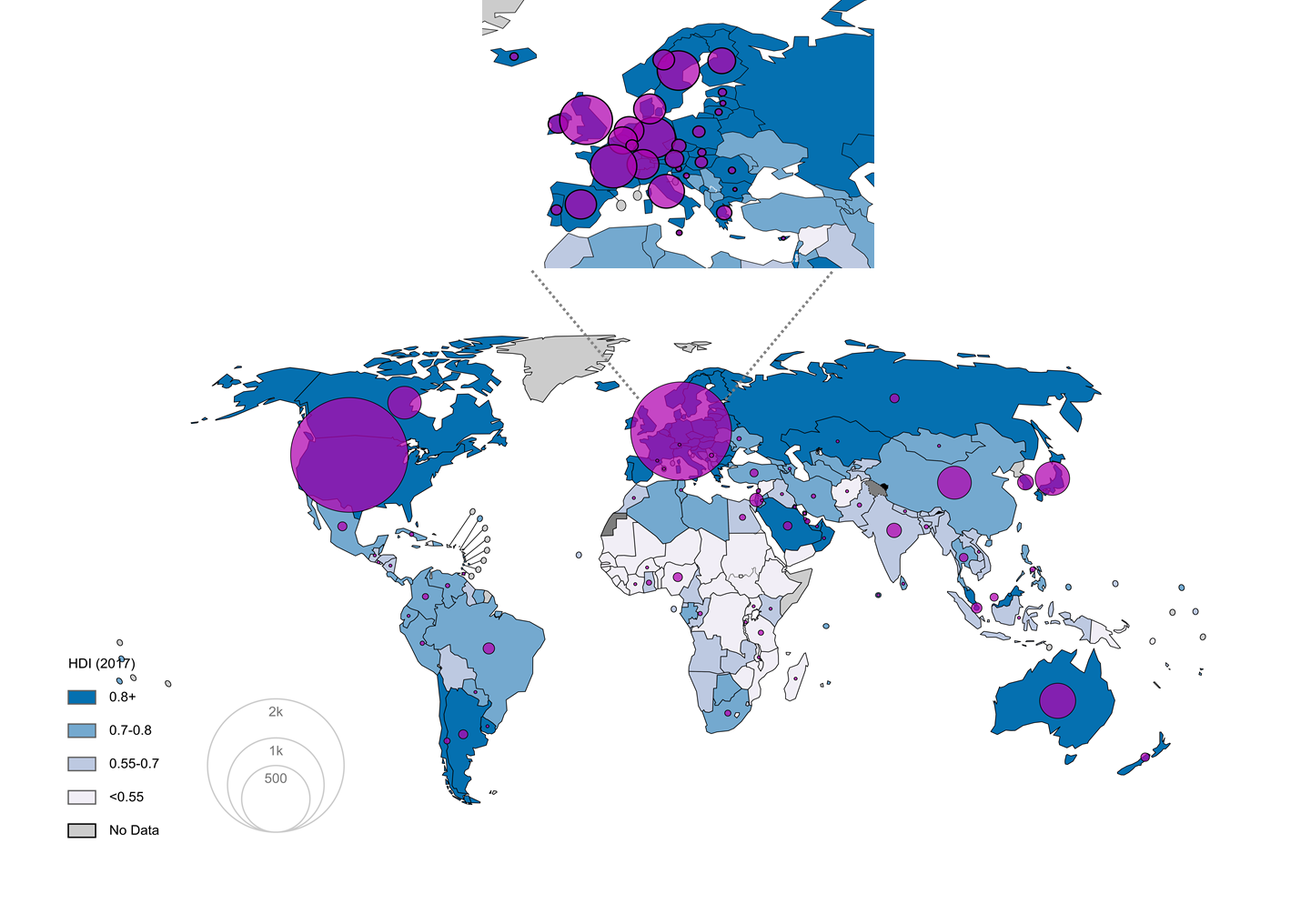

Supplement: pkz069_Supplementary_Data [file pkz069_supplementary_data.zip › SCHMUTZ_Cancer_Research_Funding_Mapping_Figure3.docx]

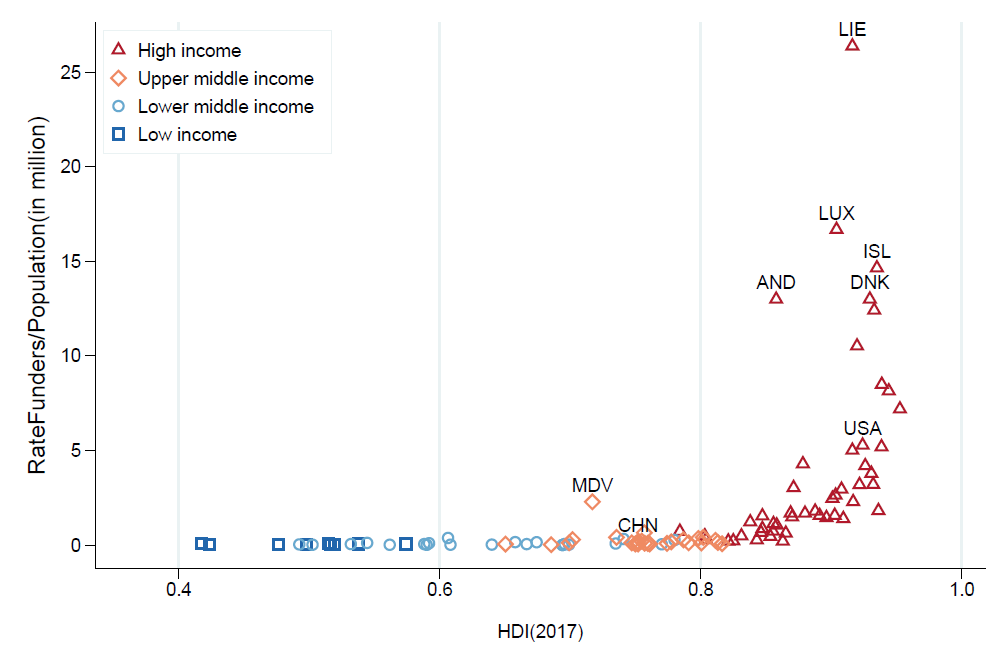

Supplement: pkz069_Supplementary_Data [file pkz069_supplementary_data.zip › SCHMUTZ_Cancer_Research_Funding_Mapping_Figure4.docx]

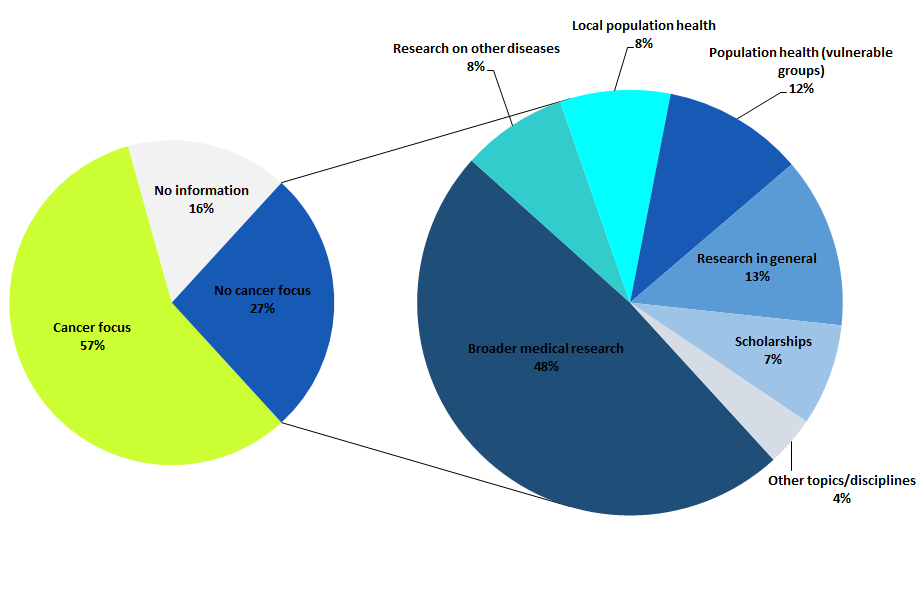

Supplement: pkz069_Supplementary_Data [file pkz069_supplementary_data.zip › SCHMUTZ_Cancer_Research_Funding_Mapping_Figure5.docx]
